# Supplementary material for: Novel Approaches for the Solid-Phase Synthesis of Dihydroquinazoline-2(1H)-One Derivatives and Biological Evaluation as Potential Anticancer Agents
Source: Molecules. 2022 Dec 5;27(23):8577. doi: 10.3390/molecules27238577 (PMC9737648; doi:10.3390/molecules27238577)
Supplement: Supplementary file 1 [file molecules-27-08577-s001.zip › Supporting Information.pdf]

Supporting Information

Novel Approaches for the Solid-Phase Synthesis of Dihydroquinazoline-2(1H)-one Derivatives and Biological Evaluation as Potential Anticancer Agents

Qiong Wang[1,4], Ying Pan[1], Hongjun Luo[2], Yanmei Zhang[3], Fenfei Gao[3], Jinzhi Wang[1,*], Jinhong Zheng[1,*]

1 Department of Chemistry, Shantou University Medical College, 22 Xinling Road, Shantou 515041, PR China

2 Department of Bio-analytical, Shantou University Medical College, 22 Xinling Road, Shantou 515041, PR China

3 Department of Pharmacology, Shantou University Medical College, Shantou 515041, PR China

4 National Cancer Center/National Clinical Research Center for Cancer/Cancer Hospital & Shenzhen Hospital, Chinese Academy of Medical Sciences and Peking Union Medical College, Shenzhen, 518116, PR China

*Corresponding author:

Jinzhi Wang, Ph.D.

Department of Chemistry, Shantou University Medical College, 22 Xinling Road, Shantou 515041, PR China, Email: jzhwang@stu.edu.cn

Jinhong Zheng, Ph.D.

Department of Chemistry, Shantou University Medical College, 22 Xinling Road, Shantou 515041, PR China, Email: jhzheng@stu.edu.cn

Table of Contents

Analytical data of synthetic compounds 3

^{1}H NMR and ^{13}C NMR Spectrometry 14

LC/MS Spectrometry 35

Analytical data of synthetic compounds

N-Fmoc-3-amino-3-(2-fluoro-5-nitrophenyl)propionic acid 3':

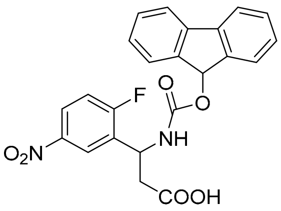

White solid; yield: 68.2%; mp 200.8-201.2℃; ^{1}H NMR (600 MHz, DMSO) δ 8.36 (dd, *J* = 6.1, 2.8 Hz, 1H), 8.22 (dd, *J* = 8.7, 3.9 Hz, 2H), 7.87 (t, *J* = 7.2 Hz, 2H), 7.65 (dd, *J* = 15.3, 7.5 Hz, 2H), 7.49 (t, *J* = 9.1 Hz, 1H), 7.39 (dt, *J* = 11.9, 7.5 Hz, 2H), 7.27 (dt, *J* = 7.2, 3.6 Hz, 2H), 5.27 (td, *J* = 8.6, 6.1 Hz, 1H), 4.34 – 4.28 (m, 2H), 4.20 (t, *J* = 6.8 Hz, 1H), 2.74 (ddd, *J* = 22.1, 16.2, 7.5 Hz, 2H); ^{13}C NMR (151 MHz, DMSO) δ 171.60, 164.11, 162.41(C-F, d, $^1J_{C\text{-}F}$ = 255.86 Hz), 155.75, 144.63, 144.30, 143.98, 141.19, 141.16, 132.24, 132.13(C-F, d, $^2J_{C\text{-}F}$ = 16.5 Hz), 128.12, 128.06 (C-F, d, $^3J_{C\text{-}F}$ = 8.68 Hz) ,127.48, 127.45, 125.71， 125.64 (C-F, d, $^3J_{C\text{-}F}$ = 10.61 Hz), 125.54, 125.52, 124.36, 124.32, 120.63, 120.57, 117.61, 117.44(C-F, d, $^2J_{C\text{-}F}$ = 24.60 Hz), 65.94, 47.09, 45.6; MS (ESI) (m/z): 450.12, [M+H$^+$]: 451.10.

3-{4-{2-[(1-amino-3-hydroxy-1-oxopropan-2-yl)amino]-2-oxoethyl}-6-nitro-2-oxo-3,4-dihydroquinazolin-1(2H)-yl}propan-1-aminium 2,2,2-trifluoroacetate (CA1-6)·TFA:

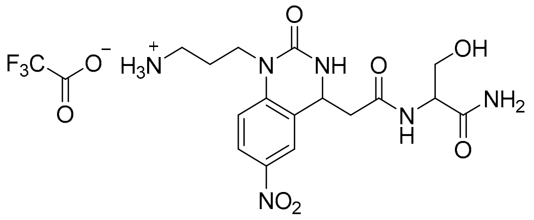

Yellow solid; yield 93%; ^{1}H NMR (600 MHz, DMSO) δ 8.15 – 8.10 (m, 1H), 8.07 (dd, *J* = 25.3, 2.5 Hz, 1H), 7.96 (d, *J* = 8.0 Hz, 1H), 7.86 (s, 3H), 7.52 (dd, *J* = 53.4, 2.7 Hz, 1H), 7.34 – 7.19 (m, 2H), 7.08 (d, *J* = 9.3 Hz, 1H), 4.89 (dtd, *J* = 25.9, 6.5, 2.8 Hz, 1H), 4.19 (td, *J* = 10.0, 5.1 Hz, 1H), 3.97 – 3.90 (m, 2H), 3.56 – 3.39 (m, 2H), 2.90 (d, *J* = 5.8 Hz, 2H), 2.70 – 2.53 (m, 2H), 1.97 – 1.75 (m, 2H); ^{13}C NMR (151 MHz, DMSO) δ 172.39 (d, *J* = 6.2 Hz), 169.27, 158.74 (C-F, q, $^2J_{C\text{-}F}$ = 31.6 Hz), 153.15, 143.77, 141.54, 124.69 , 124.22, 122.70, 117.46 (C-F, q, $^1J_{C\text{-}F}$ = 299.3 Hz), 114.01, 62.08, 55.25, 50.07, 44.45, 37.07, 25.46; MS (ESI) (m/z): 394. 16, [M+H$^+$]:

395.15.

4-{2-[(1-amino-3-hydroxy-1-oxopropan-2-yl)amino]-2-oxoethyl}-1-(3-ammoniopropyl)-2-oxo-1,2,3,4-tetrahydroquinazolin-6-aminium 2,2,2-trifluoroacetate (CA1-7)·TFA:

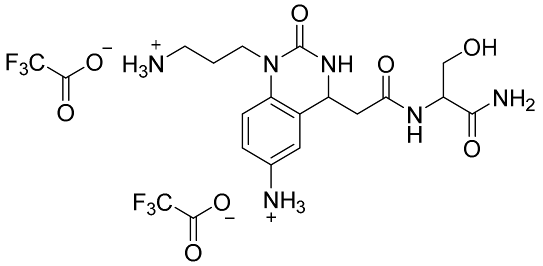

Brown solid; yield 76%; ^{1}H NMR (600 MHz, DMSO) δ 7.99 (dd, *J* = 20.8, 7.9 Hz, 1H), 7.88 (s, 1H), 7.33 (d, *J* = 14.9 Hz, 1H), 7.13 (ddd, *J* = 30.6, 18.0, 5.7 Hz, 2H), 7.05 (d, *J* = 1.8 Hz, 1H), 4.76 (dtd, *J* = 19.5, 6.6, 2.7 Hz, 1H), 4.23 (dd, *J* = 12.3, 5.5 Hz, 1H), 3.93 – 3.74 (m, 1H), 3.56 (dddd, *J* = 31.8, 26.5, 10.2, 5.4 Hz, 1H), 2.94 – 2.83 (m, 1H), 2.55 (td, *J* = 14.8, 7.5 Hz, 1H), 1.93 – 1.75 (m, 1H); ^{13}C NMR (151 MHz, DMSO) δ 172.52, 169.60, 159.08, 158.86 (C-F, q, $^2J_{C\text{-}F}$ = 32.4 Hz), 154.02, 135.78, 125.03, 121.84, 120.15, 119.92,.03, 118.23, 116.25 (C-F, q, $^1J_{C\text{-}F}$ = 299.3 Hz), 114.55, 62.02, 55.46, 50.23, 44.42, 39.05, 37.13, 25.48; MS (ESI) (m/z): 364.19, [M+H$^+$]: 365.20.

3-{4-{2-[(1-amino-3-hydroxy-1-oxopropan-2-yl)amino]-2-oxoethyl}-6-(isonicotinamido)-2-oxo-3,4-dihydroquinazolin-1(2H)-yl}propan-1-aminium 2,2,2-trifluoroacetate (CA1-a)·TFA:

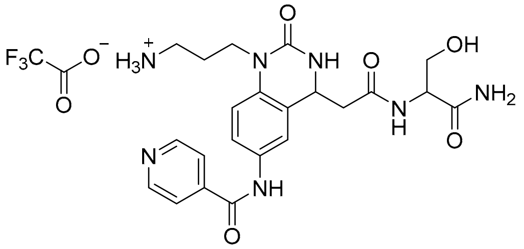

Orange solid; yield 86%; ^{1}H NMR (600 MHz, DMSO) δ 10.53 (d, *J* = 19.5 Hz, 1H), 8.85 (s, 2H), 8.06 – 7.93 (m, 3H), 7.79 (s, 3H), 7.68 (ddd, *J* = 18.6, 8.8, 2.3 Hz, 1H), 7.62 (s, 1H), 7.32 (d, *J* = 14.5 Hz, 1H), 7.18 – 7.11 (m, 1H), 7.11 – 7.00 (m, 2H), 4.80 (dd, *J* = 9.3, 3.8 Hz, 1H), 4.26 (dt, *J* = 7.8, 5.4 Hz, 1H), 3.94 – 3.84 (m, 2H), 3.54 (ddd, *J* = 15.9, 10.9, 5.5 Hz, 2H), 2.89 (dd, *J* = 12.5, 6.2 Hz, 2H), 2.73 – 2.52 (m, 2H), 1.88 (ddt, *J* = 19.1, 13.6, 6.7 Hz, 2H); ^{13}C NMR (151 MHz, DMSO) δ 172.58, 169.80, 163.55, 158.94 (C-F, q, $^2J_{C\text{-}F}$ = 32.4 Hz), 154.15, 149.58, 143.49,

134.05, 133.18, 124.04, 122.58, 120.97, 119.13 (C-F, q, ${}^{1}J_{C\text{-}F}$ = 247.39 Hz), 113.79, 62.07, 55.54, 50.69, 44.73, 38.89, 37.19, 25.65; MS (ESI) (m/z): 469.21, [M+H^{+}]: 470.15.

3-{4-{2-[(1-amino-3-hydroxy-1-oxopropan-2-yl)amino]-2-oxoethyl}-6-benzamido-2-oxo-3,4-dihydroquinazolin-1(2H)-yl}propan-1-aminium 2,2,2-trifluoroacetate (CA1-b)·TFA:

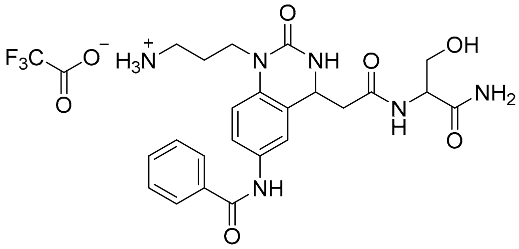

Yellow solid; yield 85%; ^{1}H NMR (600 MHz, DMSO) δ 10.19 (d, *J* = 12.5 Hz, 1H), 8.04 – 7.97 (m, 1H), 7.97 – 7.92 (m, 2H), 7.74 (s, 3H), 7.70 – 7.65 (m, 1H), 7.65 – 7.62 (m, 1H), 7.60 (t, *J* = 7.3 Hz, 1H), 7.54 (td, *J* = 7.6, 1.7 Hz, 2H), 7.32 (d, *J* = 21.9 Hz, 1H), 7.10 (d, *J* = 10.7 Hz, 1H), 7.08 – 6.96 (m, 2H), 4.81 – 4.73 (m, 1H), 4.26 (dq, *J* = 7.7, 5.4 Hz, 1H), 3.88 (dtt, *J* = 21.2, 14.3, 7.0 Hz, 2H), 2.89 (dd, *J* = 12.6, 6.5 Hz, 2H), 2.65 – 2.52 (m, 2H), 1.94 – 1.82 (m, 2H); ^{13}C NMR (151 MHz, DMSO) δ 172.52, 169.79, 165.64, 158.62, 158.39 (C-F, q, ${}^{2}J_{C\text{-}F}$ = 34.82 Hz), 154.18, 135.25, 133.90, 133.51, 132.00, 128.88, 128.02, 123.94, 120.83, 119.01, 113.69 (C-F, q, ${}^{1}J_{C\text{-}F}$ = 275.78 Hz), 62.11, 55.48, 50.67, 44.71, 38.87, 37.22, 25.67; MS (ESI) (m/z): 468.21, [M+H^{+}]: 469.20.

3-{4-{2-[(1-amino-3-hydroxy-1-oxopropan-2-yl)amino]-2-oxoethyl}-6-(4-methylbenzamido)-2-oxo-3,4-dihydroquinazolin-1(2H)-yl}propan-1-aminium 2,2,2-trifluoroacetate (CA1-c)·TFA:

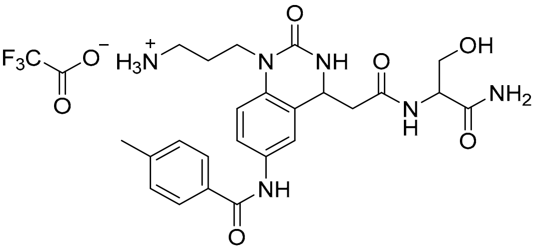

White solid; yield 82%; ^{1}H NMR (600 MHz, DMSO) δ 10.10 (d, *J* = 11.8 Hz, 1H), 7.99 (dd, *J* = 40.7, 7.9 Hz, 1H), 7.88 (dd, *J* = 10.2, 8.2 Hz, 2H), 7.73 (s, 3H), 7.67 (ddd, *J* = 14.4, 8.8, 2.1 Hz, 1H), 7.62 (s, 1H), 7.37 – 7.28 (m, 3H), 7.10 (d, *J* = 9.6 Hz, 1H), 7.07 – 6.95 (m, 2H), 4.81 – 4.72 (m, 1H), 4.26 (dq, *J* = 7.7, 5.4 Hz, 1H), 3.88 (pd, *J* = 14.2, 6.7 Hz, 2H), 3.59 (dd, *J* = 10.6,

5.5 Hz, 2H), 2.88 (dd, J = 12.6, 6.3 Hz, 2H), 2.63 – 2.52 (m, 2H), 2.39 (s, 3H), 1.93 – 1.83 (m, 2H); ^{13}C NMR (151 MHz, DMSO) δ 172.56, 169.88, 169.73 (C-F, q, $^{2}J_{C\text{-}F}$ = 22.39 Hz), 165.43, 154.17, 142.01, 133.98, 133.41, 132.40, 132.34, 129.40, 128.05, 120.81, 119.00 (C-F, q, $^{1}J_{C\text{-}F}$ = 279.55 Hz), 118.32, 113.66, 62.09, 55.49, 50.67, 44.70, 38.82, 37.22, 25.67, 21.47; MS (ESI) (m/z): 482.23, [M+H$^+$]: 483.20.

3-{4-{2-[(1-amino-3-hydroxy-1-oxopropan-2-yl)amino]-2-oxoethyl}-6-(4-fluorobenzamido)-2-oxo-3,4-dihydroquinazolin-1(2H)-yl}propan-1-aminium 2,2,2-trifluoroacetate (CA1-d)·TFA:

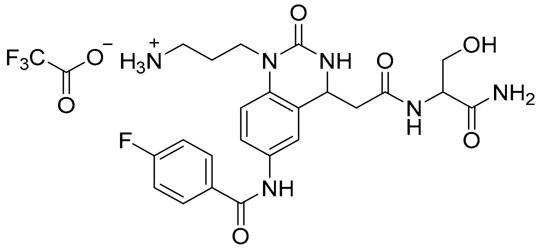

Yellow solid; yield 79%; ^{1}H NMR (600 MHz, DMSO) δ 10.20 (d, J = 16.2 Hz, 1H), 8.11 – 7.89 (m, 3H), 7.73 (s, 3H), 7.66 (ddd, J = 17.0, 8.9, 2.2 Hz, 1H), 7.61 (s, 1H), 7.43 – 7.35 (m, 2H), 7.32 (d, J = 25.3 Hz, 1H), 7.10 (d, J = 13.3 Hz, 1H), 7.08 – 6.96 (m, 2H), 4.83 – 4.71 (m, 1H), 4.29 – 4.22 (m, 1H), 3.95 – 3.83 (m, 2H), 2.89 (dd, J = 12.5, 6.3 Hz, 2H), 2.64 – 2.52 (m, 2H), 1.88 (ddd, J = 21.5, 10.8, 5.5 Hz, 2H); ^{13}C NMR (151 MHz, DMSO) δ 172.57, 169.77, 164.55, 163.69 (C-F, q, $^{1}J_{C\text{-}F}$ = 249.32 Hz), 158.38 (C-F, q, $^{2}J_{C\text{-}F}$ = 31.83 Hz), 154.15, 133.78, 133.56, 131.69, 130.75, 123.95, 120.83, 119.02 (C-F, q, $^{1}J_{C\text{-}F}$ = 274.60 Hz), 115.88, 115.74, 113.70, 62.10, 55.49, 50.66, 44.65, 38.84, 37.19, 25.66; MS (ESI) (m/z): 486.20, [M+H$^+$]: 487.20.

3-{4-{2-[(1-amino-3-hydroxy-1-oxopropan-2-yl)amino]-2-oxoethyl}-6-(nicotinamido)-2-oxo-3,4-dihydroquinazolin-1(2H)-yl}propan-1-aminium 2,2,2-trifluoroacetate (CA1-e)·TFA:

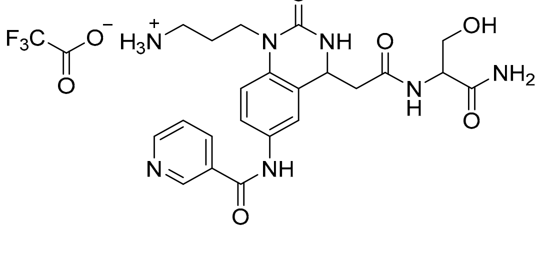

Yellow solid; yield 84%; ^{1}H NMR (600 MHz, DMSO) δ 10.45 (d, *J* = 15.6 Hz, 1H), 9.15 (d, *J* = 8.4 Hz, 1H), 8.80 (d, *J* = 4.6 Hz, 1H), 8.38 (ddd, *J* = 9.7, 8.0, 4.0 Hz, 1H), 7.99 (dd, *J* = 39.8, 7.9 Hz, 1H), 7.79 (s, 3H), 7.67 (dd, *J* = 11.7, 3.1 Hz, 1H), 7.65 – 7.63 (m, 1H), 7.62 (dd, *J* = 6.4, 2.2 Hz, 1H), 7.31 (s, 1H), 7.10 (dd, *J* = 12.0, 9.7 Hz, 2H), 7.07 – 6.99 (m, 1H), 4.80 – 4.74 (m, 1H), 4.27 – 4.25 (m, 1H), 3.93 – 3.82 (m, 2H), 3.62 – 3.48 (m, 2H), 2.89 (dd, *J* = 12.6, 6.3 Hz, 2H), 2.64 – 2.52 (m, 2H), 1.94 – 1.83 (m, 2H); ^{13}C NMR (151 MHz, DMSO) δ 172.58, 169.75, 163.82, 158.67 (C-F, q, $^2J_{C\text{-}F}$ = 35.9 Hz), 154.17, 151.69, 148.35, 136.83, 133.83, 133.47, 131.23, 124.40, 124.02, 120.84, 119.02, 117.32, 115.38 (C-F, q, $^1J_{C\text{-}F}$ = 283.7 Hz), 113.77, 62.06, 55.47, 50.65, 44.65, 38.88, 37.20, 25.66; MS (ESI) (m/z): 469.21, [M+H$^+$]: 470.20.

3-{4-{2-[(1-amino-3-hydroxy-1-oxopropan-2-yl)amino]-2-oxoethyl}-2-oxo-6-(picolinamido)-3,4-dihydroquinazolin-1(2H)-yl}propan-1-aminium 2,2,2-trifluoroacetate (CA1-f)·TFA:

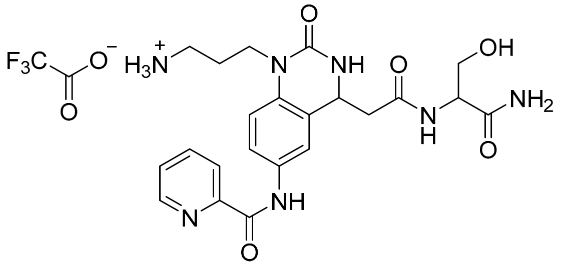

Orange solid; yield 76%; ^{1}H NMR (600 MHz, DMSO) δ 10.67 – 10.53 (m, 1H), 8.79 – 8.69 (m, 1H), 8.15 (d, *J* = 7.8 Hz, 1H), 8.07 (t, *J* = 7.7 Hz, 1H), 7.99 (dd, *J* = 17.4, 7.9 Hz, 1H), 7.78 (dd, *J* = 7.1, 4.4 Hz, 5H), 7.68 (dd, *J* = 6.6, 4.9 Hz, 1H), 7.29 (s, 1H), 7.08 (d, *J* = 7.3 Hz, 1H), 7.01 (dd, *J* = 52.7, 2.0 Hz, 2H), 4.84 – 4.74 (m, 1H), 4.33 – 4.20 (m, 1H), 3.97 – 3.80 (m, 2H), 3.65 – 3.49 (m, 2H), 2.89 (dt, *J* = 12.7, 6.3 Hz, 2H), 2.65 – 2.51 (m, 2H), 1.97 – 1.78 (m, 2H); ^{13}C NMR (151 MHz, DMSO) δ 172.54, 169.82, 162.62, 158.88 (C-F, q, $^2J_{C\text{-}F}$ = 34.93 Hz), 154.11, 150.32, 148.88, 138.61, 133.77, 133.07, 127.33, 123.94, 122.71, 120.74, 118.90, 117.17 (C-F, q, $^1J_{C\text{-}F}$ = 71.71 Hz), 113.78, 62.08, 55.48, 50.68 (d, *J* = 12.5 Hz), 44.58, 38.88, 37.20, 25.66; MS (ESI) (m/z): 469.21, [M+H$^+$]: 470.15.

3-{4-{2-[(1-amino-3-hydroxy-1-oxopropan-2-yl)amino]-2-oxoethyl}-2-oxo-6-(2-phenoxyacetamido)-3,4-dihydroquinazolin-1(2H)-yl}propan-1-aminium 2,2,2-trifluoroacetate (CA1-g)·TFA:

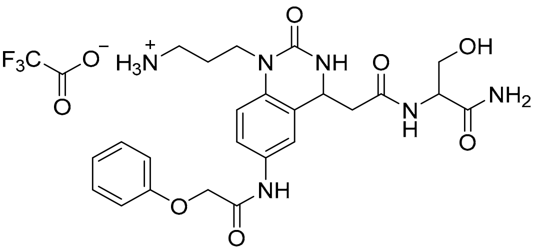

White solid; yield 85%; ^{1}H NMR (600 MHz, DMSO) δ 10.03 (d, J = 14.1 Hz, 1H), 7.98 (dd, J = 37.5, 7.9 Hz, 1H), 7.77 (s, 3H), 7.58 – 7.51 (m, 1H), 7.4 (dd, J = 14.2, 2.0 Hz, 1H), 7.32 (t, J = 7.8 Hz, 2H), 7.30 (d, J = 3.8 Hz, 1H), 7.10 (s,1H), 7.07 – 6.95 (m, 5H), 4.87 (d, J = 34.2 Hz, 1H), 4.78 – 4.69 (m, 1H), 4.60 (d, J = 4.2 Hz, 2H), 4.24 (dq, J = 10.4, 5.2 Hz, 1H), 3.93 – 3.79 (m, 2H), 3.55 (d, J = 42.7 Hz, 2H), 2.87 (d, J = 5.4 Hz, 2H), 2.62 – 2.51 (m, 2H), 1.86 (dq, J = 12.8, 6.9 Hz, 2H); ^{13}C NMR (151 MHz, DMSO) δ 172.63, 172.54, 169.73, 163.55, 158.83 (C-F, q, $^2J_{C\text{-}F}$ = 30.59 Hz), 154.12, 149.83, 149.58, 143.49, 134.05, 133.18, 124.04, 122.58, 120.97, 119.13, 115.48 (C-F, q, $^1J_{C\text{-}F}$ = 255.65 Hz), 113.79, 62.07, 55.48, 51.80, 50.63, 44.73, 38.92, 37.19, 25.65; MS (ESI) (m/z): 498.22, [M+H$^+$]: 499.20.

3-{4-{2-[(1-amino-3-hydroxy-1-oxopropan-2-yl)amino]-2-oxoethyl}-2-oxo-6-[3-(pyridin -3-yl)propanamido]-3,4-dihydroquinazolin-1(2H)-yl}propan-1-aminium 2,2,2-trifluoroacetate (CA1-h)·TFA:

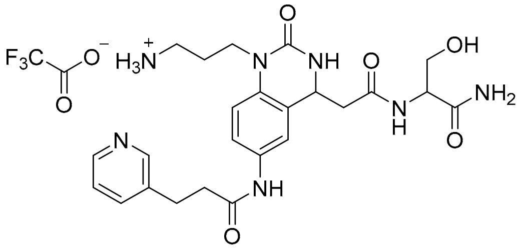

Yellow solid; yield 92%; ^{1}H NMR (600 MHz, DMSO) δ 9.96 (d, J = 15.4 Hz, 1H), 8.78 (s, 1H), 8.70 (s, 1H), 8.27 (d, J = 7.6 Hz, 1H), 7.98 (dd, J = 38.9, 7.9 Hz, 1H), 7.85 (s, 3H), 7.83 – 7.80 (m, 1H), 7.42 (s, 1H), 7.40 (dd, J = 5.0, 2.6 Hz, 1H), 7.30 (d, J = 6.5 Hz, 1H), 7.11 (d, J = 7.3 Hz, 1H), 7.06 – 6.92 (m, 2H), 4.83 – 4.65 (m, 1H), 4.24 (dtd, J = 7.8, 5.3, 2.4 Hz, 1H), 3.89 – 3.79 (m, 2H), 3.64 – 3.44 (m, 2H), 3.06 (t, J = 7.1 Hz, 2H), 2.87 (dd, J = 12.3, 6.2 Hz, 2H), 2.74 (d, J = 7.2 Hz, 2H), 2.65 – 2.51 (m, 2H), 1.93 – 1.78 (m, 2H); ^{13}C NMR (151 MHz, DMSO) δ 172.55, 169.86, 169.68, 159.02, 158.80 (C-F, q, $^2J_{C\text{-}F}$ = 33.5 Hz), 154.18, 144.44, 143.33, 142.52, 140.36, 133.79, 133.09, 130.88, 126.24, 124.05, 119.56, 118.02, 117.71, 116.06 (C-F, q, $^1J_{C\text{-}F}$ =

294.9 Hz), 113.78, 62.06, 55.47, 50.58, 44.56, 38.84, 37.14, 36.86, 28.04, 25.63; MS (ESI) (m/z): 497.24, [M+H$^+$]: 498.25.

3-{4-{2-[(1-amino-3-hydroxy-1-oxopropan-2-yl)amino]-2-oxoethyl}-2-oxo-6-[2-(thiophen-2-yl)acetamido]-3,4-dihydroquinazolin-1(2H)-yl}propan-1-aminium 2,2,2-trifluoroacetate (CA1-i)·TFA:

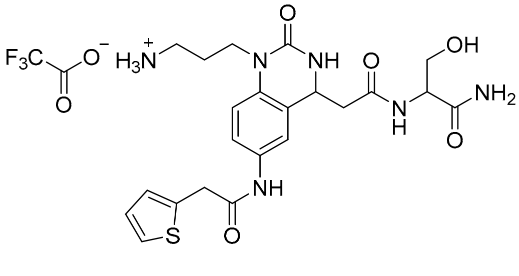

Pale brown solid; yield 90%; ^{1}H NMR (600 MHz, DMSO) δ 10.18 (d, J = 15.2 Hz, 1H), 7.99 (dd, J = 38.4, 7.9 Hz, 1H), 7.8 (s, 3H), 7.45 (dd, J = 23.6, 5.1 Hz, 2H), 7.41 – 7.35 (m, 1H), 7.30 (d, J = 4.2 Hz, 1H), 7.10 (d, J = 9.6 Hz, 1H), 7.08 – 6.90 (m, 4H), 4.77 – 4.67 (m, 1H), 4.28 – 4.21 (m, 1H), 3.86 (t, J = 9.1 Hz, 4H), 3.61 – 3.48 (m, 2H), 2.93 – 2.82 (m, 2H), 2.62 – 2.51 (m, 2H), 1.86 (ddd, J = 22.5, 10.5, 4.8 Hz, 2H); ^{13}C NMR (151 MHz, DMSO) δ 172.56, 169.79, 168.19, 158.70 (C-F, q, $^2J_{C\text{-}F}$ = 32.3 Hz), 154.15, 137.64, 133.80, 133.27, 129.82, 127.11, 126.77, 125.49, 124.12, 119.62, 117.82, 115.69 (C-F, q, $^1J_{C\text{-}F}$ = 321.4 Hz), 113.82, 62.06, 55.46, 50.57, 44.53, 38.83, 37.86, 37.16, 25.63; MS (ESI) (m/z): 488.18, [M+H$^+$]: 489.20.

3-{4-{2-[(1-amino-3-hydroxy-1-oxopropan-2-yl)amino]-2-oxoethyl}-6-[(2-methoxyphenyl)sulfonamido]-2-oxo-3,4-dihydroquinazolin-1(2H)-yl}propan-1-aminium 2,2,2-trifluoroacetate (CA1-j)·TFA:

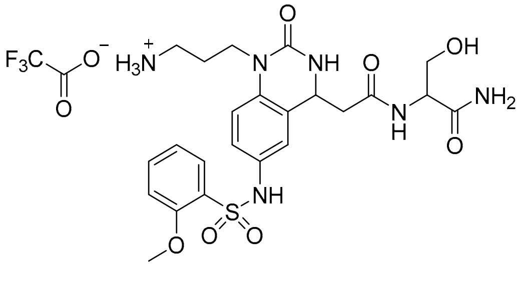

Yellow solid; yield 71%; ^{1}H NMR (600 MHz, DMSO) δ 9.73 (d, J = 35.7 Hz, 1H), 7.96 (dd, J = 10.1, 8.1 Hz, 1H), 7.75 (s, 3H), 7.72 – 7.68 (m, 1H), 7.59 – 7.54 (m, 1H), 7.33 (d, J = 24.0 Hz, 1H), 7.18 (d, J = 8.4 Hz, 1H), 7.12 (d, J = 22.3 Hz, 1H), 7.02 (td, J = 7.5, 2.7 Hz, 1H), 6.99 – 6.90 (m, 3H), 6.87 (d, J = 8.1 Hz, 1H), 4.67 – 4.59 (m, 1H), 4.24 (dt, J = 7.7, 5.3 Hz, 1H), 3.90 (s,

3H), 3.81 – 3.71 (m, 2H), 3.63 – 3.53 (m, 2H), 2.82 (d, J = 6.0 Hz, 2H), 2.41 (tt, J = 14.6, 7.2 Hz, 2H), 1.78 (ddd, J = 19.9, 11.7, 4.8 Hz, 2H); ^{13}C NMR (151 MHz, DMSO) δ 172.53, 169.57, 158.81 (C-F, q, $^{2}J_{C\text{-}F}$ = 33.8 Hz), 154.15, 153.99, 135.47, 134.17, 132.17, 130.62, 126.85, 124.46, 120.98, 120.55, 119.43, 119.62, 116.85 (C-F, q, $^{1}J_{C\text{-}F}$ = 294.6 Hz), 113.24, 62.11, 56.56, 55.45, 50.29, 44.42, 38.88, 37.14, 25.53; MS (ESI) (m/z): 534.19, [M+H^{+}]: 535.20.

5-amino-4-{2-{1-isopentyl-2-oxo-6-[3-(pyridin-3-yl)propanamido]-1,2,3,4-tetrahydroquinazolin-4-yl}acetamido}-5-oxopentan-1-aminium 2,2,2-trifluoroacetate (CA3)·TFA:

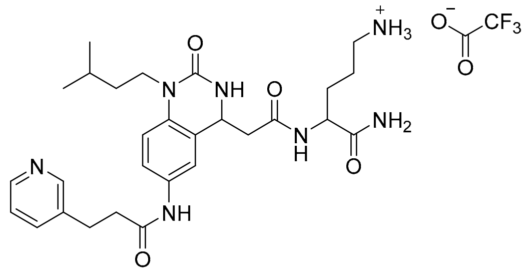

Light brown solid; yield 74%; ^{1}H NMR (600 MHz, DMSO) δ 9.95 (d, J = 18.9 Hz, 1H), 8.73 (d, J = 45.9 Hz, 1H), 8.25 (dd, J = 12.5, 8.1 Hz, 1H), 8.13 (dd, J = 34.3, 8.2 Hz, 1H), 7.80 (dd, J = 13.1, 6.9 Hz, 1H), 7.47 – 7.36 (m, 1H), 7.12 (d, J = 4.7 Hz, 1H), 6.92 – 6.85 (m, 1H), 4.70 – 4.59 (m, 1H), 4.30 – 4.18 (m, 1H), 3.91 – 3.67 (m, 1H), 3.10 – 3.02 (m, 1H), 2.82 – 2.69 (m, 2H), 2.45 (ddd, J = 14.5, 7.7, 4.1 Hz, 1H), 1.75 – 1.41 (m, 4H), 0.93 (d, J = 6.6 Hz, 3H); ^{13}C NMR (151 MHz, DMSO) δ 173.69, 169.78, 158.86 (C-F, q, $^{2}J_{C\text{-}F}$ = 31.9 Hz), 153.95, 144.62, 143.24, 142.62, 133.65, 133.47, 133.29, 126.19, 124.50, 124.30, 119.64, 117.59, 116.05, (C-F, q, $^{1}J_{C\text{-}F}$ = 292.8 Hz), 115.69, 113.79, 52.16, 51.82, 50.60, 44.28, 38.96, 36.87, 36.04, 29.27, 28.04, 26.16, 23.89, 23.04, 22.88; MS (ESI) (m/z): 537.31, [M+H^{+}]: 538.30.

2-{2-[1-benzyl-2-oxo-6-(2-phenoxyacetamido)-1,2,3,4-tetrahydroquinazolin-4-yl]acetamido}-2-methylpropanamide (CA4):

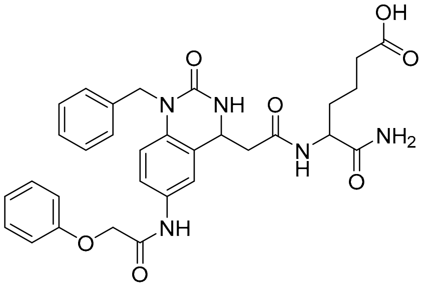

White solid; yield 75%; ^{1}H NMR (600 MHz, DMSO) δ 10.07 (d, J = 112.5 Hz, 1H), 7.95 (d, J = 13.3 Hz, 1H), 7.48 (d, J = 2.0 Hz, 1H), 7.39 – 7.26 (m, 5H), 7.23 (dd, J = 15.1, 7.5 Hz, 2H),

7.18 – 7.04 (m, 2H), 7.02 – 6.68 (m, 6H), 5.05 (d, *J* = 11.9 Hz, 1H), 4.80 – 4.62 (m, 3H), 4.52 – 4.45 (m, 1H), 2.53 (d, *J* = 6.6 Hz, 2H), 1.34 – 1.24 (m, 6H); ^{13}C NMR (151 MHz, DMSO) δ 176.74, 169.17, 166.70, 158.24, 154.24, 138.43, 133.85, 132.90, 129.96, 128.94, 127.15, 126.81, 124.02, 121.63, 120.05, 118.36, 115.08, 114.41, 67.45, 56.41, 50.70, 45.09, 25.92, 25.40; MS (ESI) (m/z): 529.23, [M+H$^+$]: 530.25.

4-{2-{1-(3-ammoniopropyl)-2-oxo-6-[2-(thiophen-2-yl)acetamido]-1,2,3,4-tetrahydroquinazolin-4-yl}acetamido}-4-carbamoylpiperidin-1-ium 2,2,2-trifluoroacetate (CA5)·TFA:

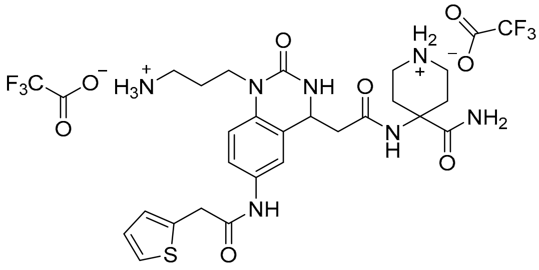

Yellowish white solid; yield 84%; ^{1}H NMR (600 MHz, DMSO) δ 10.18 (s, 1H), 8.57 (d, *J* = 8.9 Hz, 1H), 8.35 (d, *J* = 9.0 Hz, 1H), 8.14 (s, 1H), 7.79 (s, 3H), 7.49 – 7.42 (m, 2H), 7.39 (dd, *J* = 4.4, 2.0 Hz, 1H), 7.23 (s, 1H), 7.06 (d, *J* = 2.6 Hz, 1H), 7.02 – 6.97 (m, 4H), 4.69 (td, *J* = 6.6, 2.7 Hz, 1H), 3.94 – 3.88 (m, 2H), 3.85 (s, 2H), 3.13 – 2.84 (m, 6H), 2.64 – 2.54 (m, 2H), 2.13 – 2.06 (m, 2H), 1.97 (d, *J* = 10.6 Hz, 2H), 1.87 (ddd, *J* = 23.3, 14.5, 7.2 Hz, 2H); ^{13}C NMR (151 MHz, DMSO) δ 174.92, 169.92, 168.24, 158.58 (C-F, q, $^2J_{C\text{-}F}$ = 32.3Hz), 154.21, 137.63, 136.59, 133.79, 133.33, 127.15, 126.78, 125.54, 124.00, 119.58, 117.55 (C-F, q, $^1J_{C\text{-}F}$ = 305.48 Hz), 113.91, 55.89, 50.19, 44.39, 39.11, 37.87, 37.17, 29.17, 28.02, 25.61; MS (ESI) (m/z): 527.23, [M+H$^+$]: 528.20.

***N*-[4-(2-amino-2-oxoethyl)-1-methyl-2-oxo-1,2,3,4-tetrahydroquinazolin-6-yl]nicotinamide (CA6-a):**

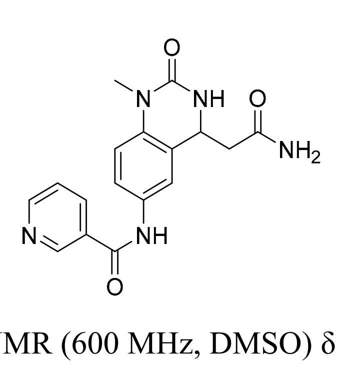

Yellow solid; yield 74%; ^{1}H NMR (600 MHz, DMSO) δ 10.48 (s, 1H), 9.17 (s, 1H), 8.82 (s, 1H), 8.42 (d, *J* = 7.9 Hz, 1H), 7.68 (dd, *J* = 8.5, 1.8 Hz, 2H), 7.59 (s, 1H), 7.35 (s, 1H), 7.03 (s,

1H), 7.00 – 6.90 (m, 2H), 4.68 (t, J = 5.4 Hz, 1H), 3.19 (s, 3H), 2.44 (dd, J = 6.8, 2.1 Hz, 2H); ^{13}C NMR (151 MHz, DMSO) δ 171.53, 163.58, 154.49, 151.18, 148.20, 147.99, 137.31, 135.56, 133.25, 124.59, 124.42, 120.77, 118.68, 113.56, 50.20, 43.85, 29.64; MS (ESI) (m/z): 339.13, [M+H$^+$]: 340.15.

***N*-[4-(2-amino-2-oxoethyl)-1-methyl-2-oxo-1,2,3,4-tetrahydroquinazolin-6-yl]-2-phenoxyacetamide (CA6-b):**

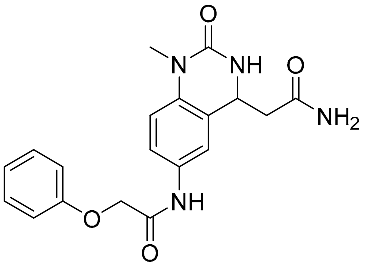

White solid; yield 78%; ^{1}H NMR (600 MHz, DMSO) δ 10.04 (s, 1H), 7.57 (dd, J = 8.8, 2.2 Hz, 1H), 7.43 (d, J = 2.1 Hz, 1H), 7.34 – 7.30 (m, 3H), 7.02 – 6.97 (m, 4H), 6.95 – 6.90 (m, 2H), 4.68 – 4.63 (m, 3H), 3.16 (s, 3H), 2.41 (dd, J = 6.8, 3.8 Hz, 2H); ^{13}C NMR (151 MHz, DMSO) δ 171.50, 166.66, 158.30, 154.47, 135.24, 132.93, 129.98, 124.43, 121.63, 120.10, 118.04, 115.14, 113.55, 67.54, 50.15, 43.77, 29.61; MS (ESI) (m/z): 368.15, [M+H$^+$]: 369.15.

2-{6-[(2-methoxyphenyl)sulfonamido]-1-methyl-2-oxo-1,2,3,4-tetrahydroquinazolin-4-yl}acetamide (CA7-a):

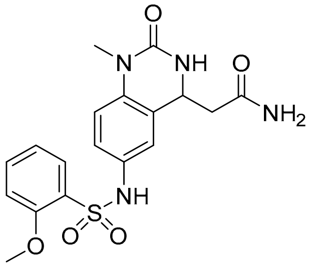

White solid; yield 84%; ^{1}H NMR (600 MHz, DMSO) δ 9.71 (s, 1H), 7.68 (dd, J = 7.8, 1.6 Hz, 1H), 7.56 – 7.52 (m, 1H), 7.27 (s, 1H), 7.17 (d, J = 8.3 Hz, 1H), 6.99 (t, J = 7.6 Hz, 1H), 6.95 (dd, J = 8.7, 2.4 Hz, 2H), 6.92 (d, J = 2.3 Hz, 1H), 6.84 (s, 1H), 6.78 (d, J = 8.8 Hz, 1H), 4.54 (td, J = 6.8, 2.9 Hz, 1H), 3.92 (s, 3H), 3.07 (s, 3H), 2.28 (ddd, J = 50.0, 14.7, 6.9 Hz, 2H); ^{13}C NMR (151 MHz, DMSO) δ 171.24, 156.76, 154.36, 135.83, 135.38, 131.95, 130.74, 126.64, 124.74, 121.05, 120.49, 119.14, 113.80, 113.17, 56.52, 49.87, 43.61, 29.55; MS (ESI) (m/z):404.12, [M+H$^+$]: 405.10.

2-{6-[(2-fluorophenyl)sulfonamido]-3-[(2-fluorophenyl)sulfonyl]-1-methyl-2-oxo-1,2,3,4-tetrahydroquinazolin-4-yl}acetamide (CA7-b'):

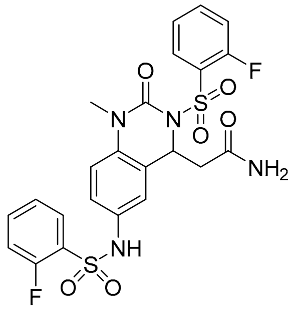

White solid; yield 76%; ^{1}H NMR (600 MHz, DMSO) δ 7.93 – 7.78 (m, 4H), 7.54 (t, J = 8.2 Hz, 2H), 7.47 (dt, J = 12.2, 7.7 Hz, 2H), 7.35 (s, 1H), 7.20 (d, J = 3.0 Hz, 1H), 7.10 (dd, J = 8.7, 2.1 Hz, 1H), 7.01 (dd, J = 7.6, 5.5 Hz, 2H), 6.94 (s, 1H), 4.65 (td, J = 6.8, 3.2 Hz, 1H), 3.18 (s, 3H), 2.32 (ddd, J = 21.4, 15.0, 6.9 Hz, 2H); ^{13}C NMR (151 MHz, DMSO) δ 171.11, 159.59, 157.89 (C-F, q, $^1J_{C\text{-}F}$ = 254.62 Hz), 153.93, 141.03, 138.46, 132.04, 131.89, 129.12, 125.74, 125.64, 124.99, 118.18, 114.20, 49.48, 43.65, 29.86; MS (ESI) (m/z): 550.08, [M+H$^+$]: 551.10.

^{1}H NMR and ^{13}C NMR Spectrometry

^{1}H NMR spectra (*d6*-DMSO) of *N*-Alloc-3-amino-3-(2-fluoro-5-nitrophenyl)propionic acid 3

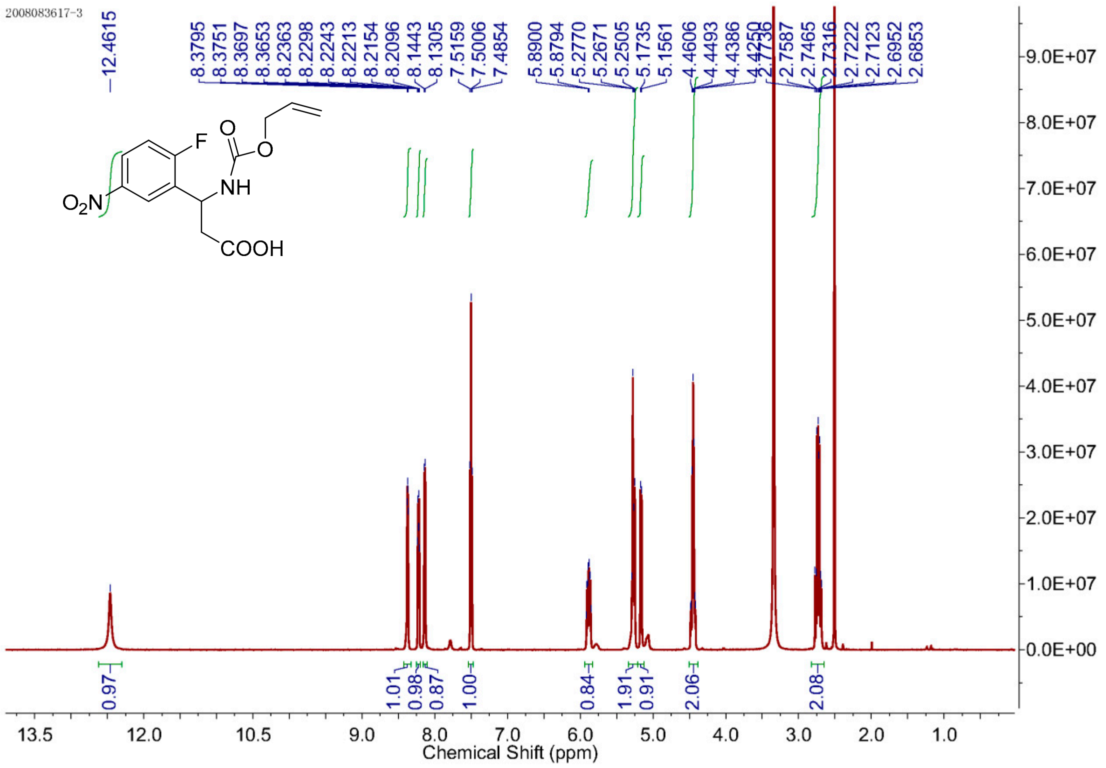

^{1}H NMR spectra (D_2O) of (CA1-g)·TFA

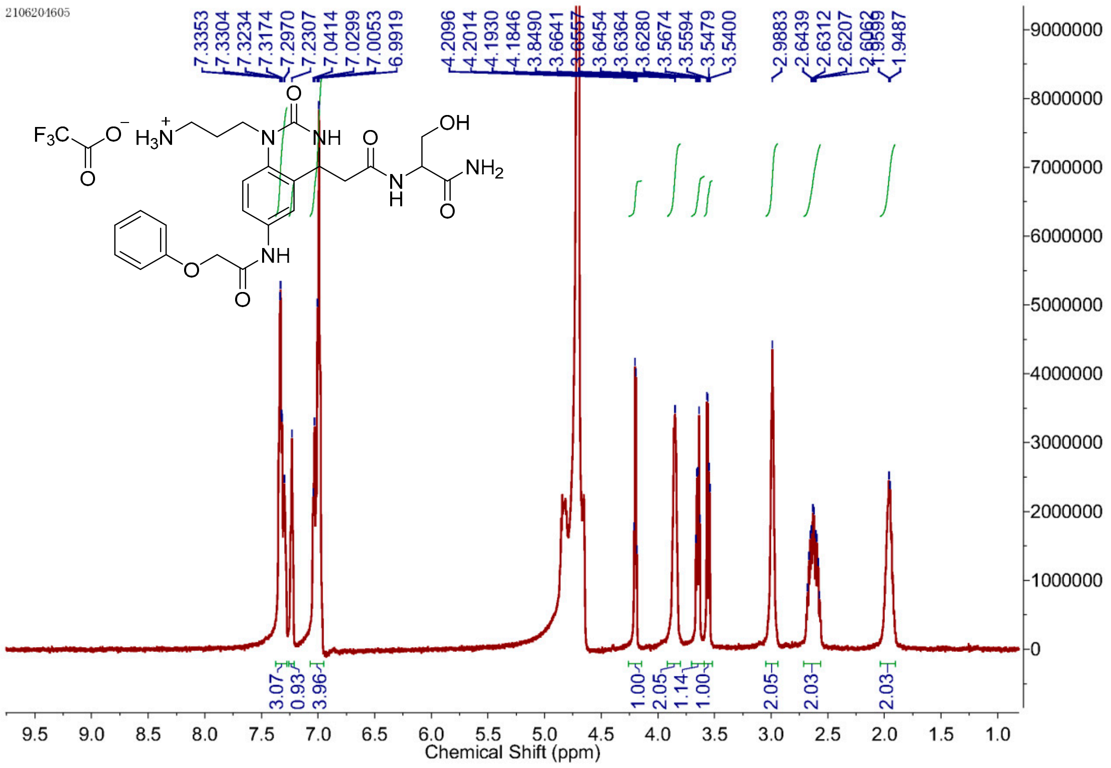

^{1}H NMR and ^{13}C NMR spectra (*d6*-DMSO) of *N*-Alloc-3-amino-3-(2-fluoro-5-nitrophenyl) propionic acid 3′

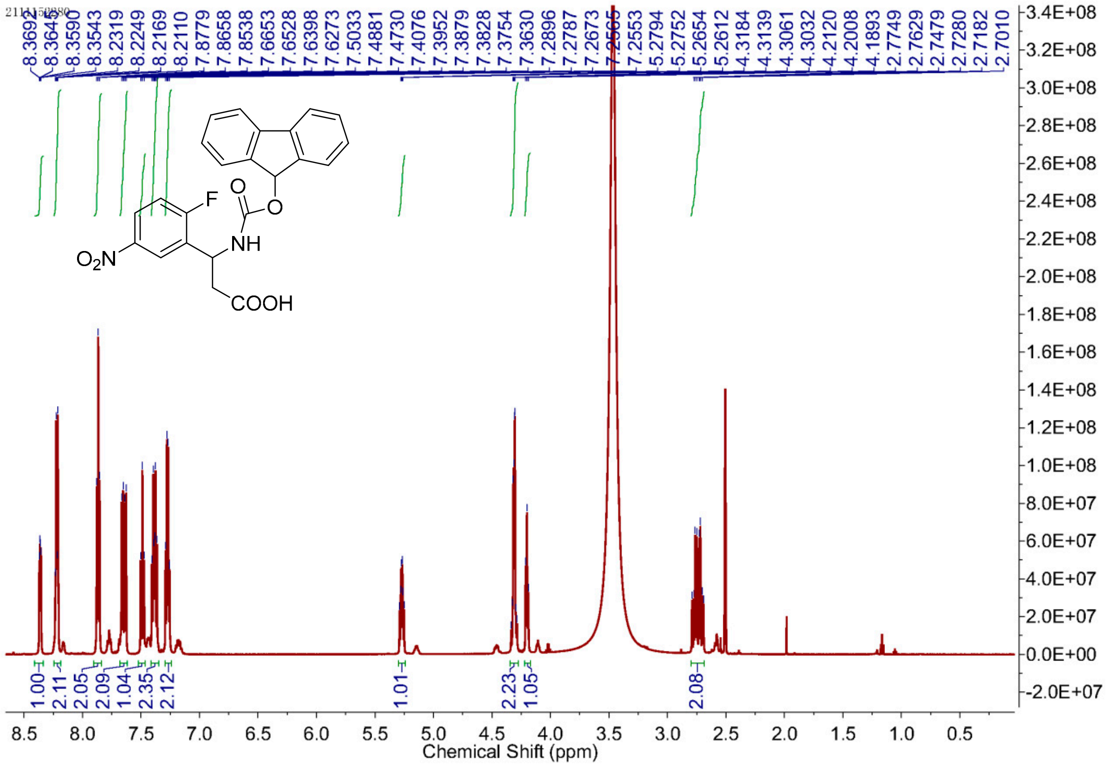

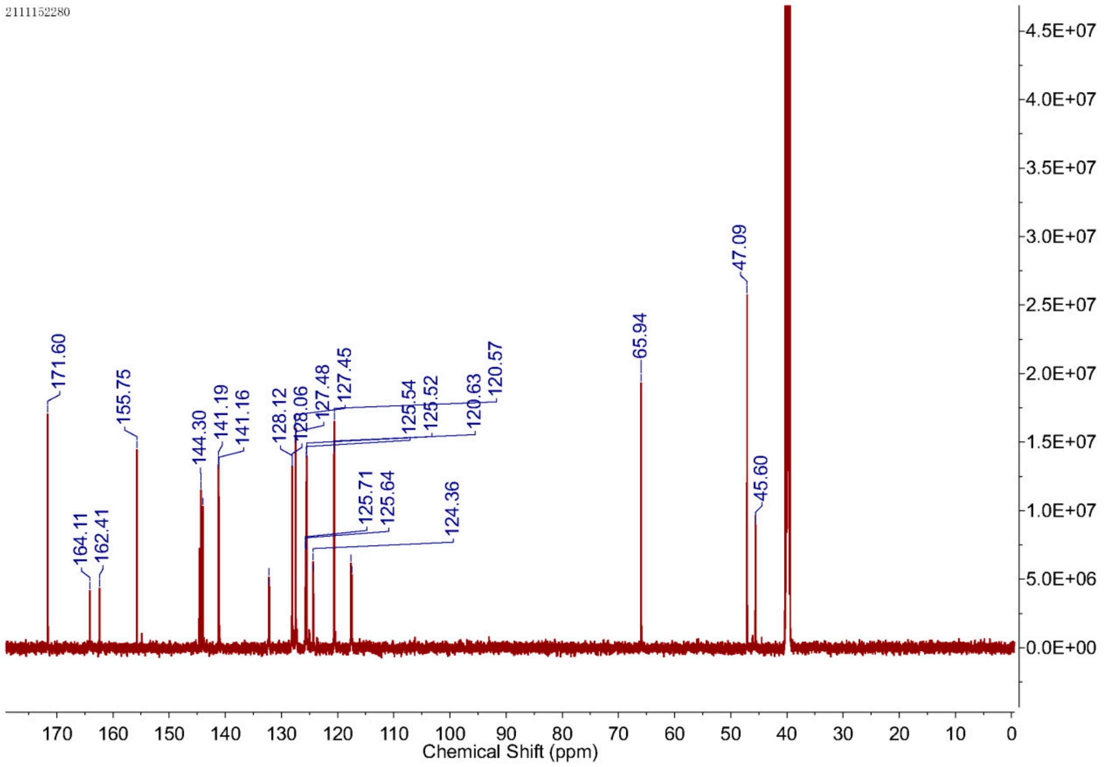

^{1}H and ^{13}C NMR (*d6*-DMSO) spectra of dihydroquinazoline-2(1H)-ones (CA1-6)·TFA

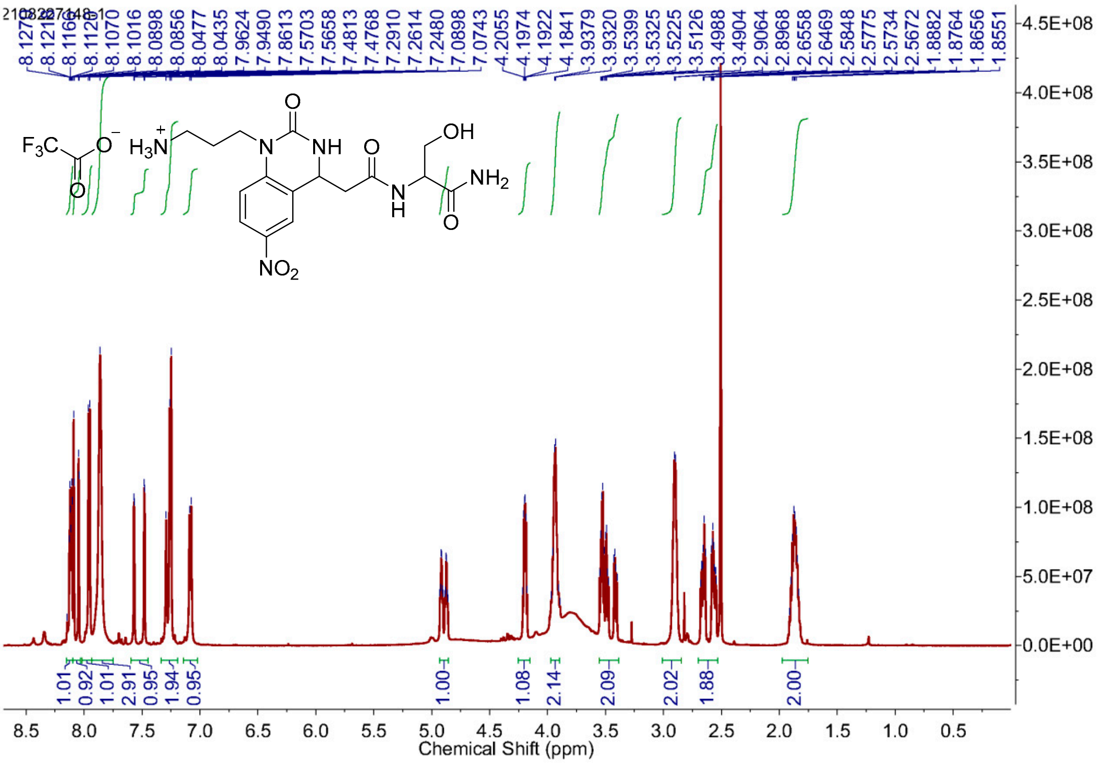

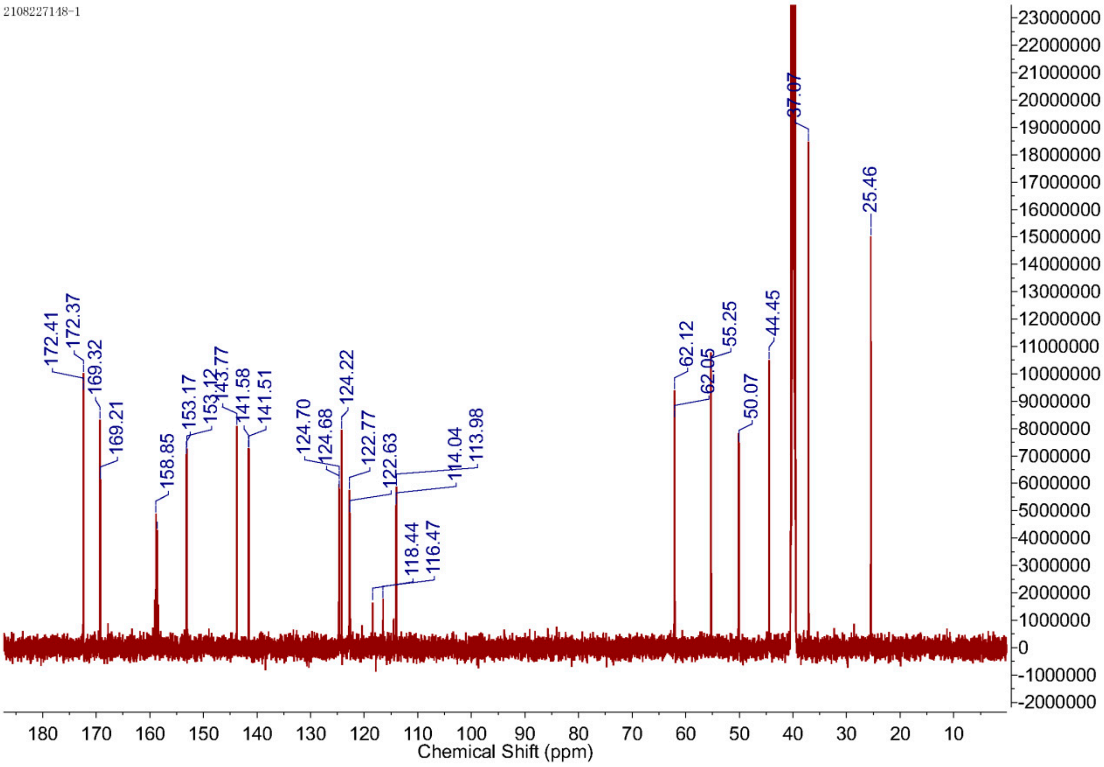

(CA1-7)·TFA

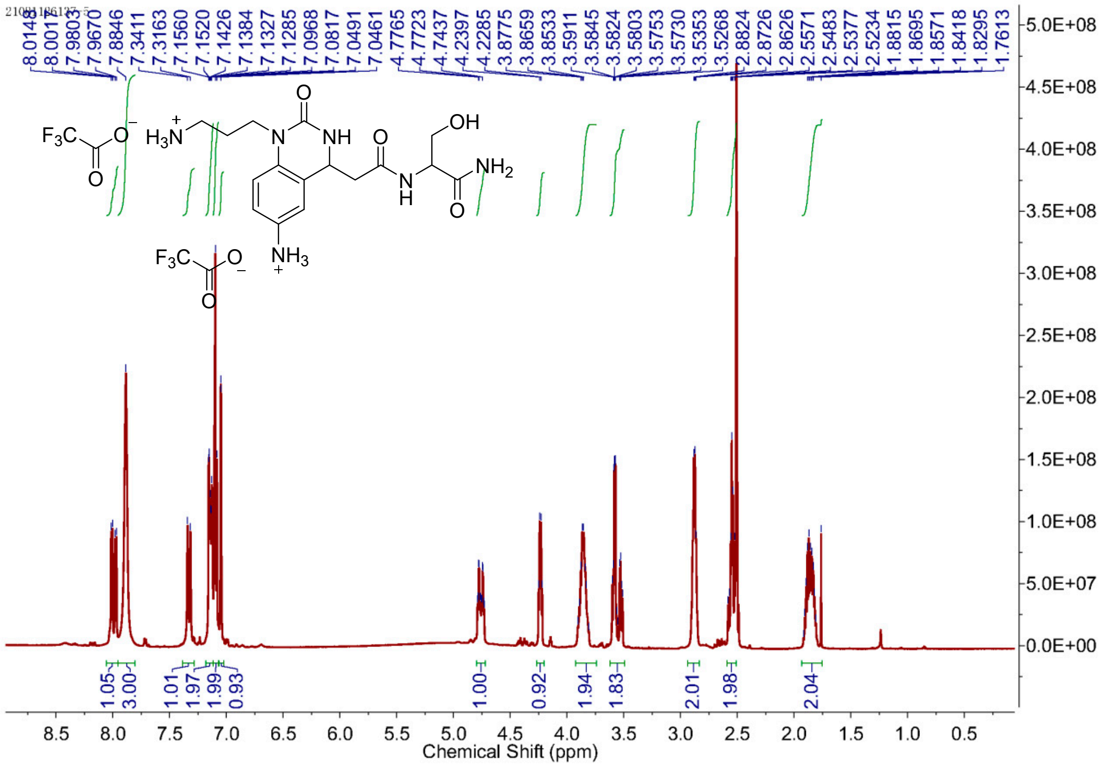
21081126137-5
8.0148
8.0011
7.9803
7.9670
7.8846
7.3411
7.3163
7.1560
7.1520
7.1426
7.1384
7.1327
7.1285
7.0968
7.0817
7.0491
7.0461
4.7765
4.7723
4.7437
4.2397
4.2285
3.8775
3.8659
3.8533
3.5911
3.5845
3.5824
3.5803
3.5753
3.5730
3.5353
3.5268
2.8824
2.8726
2.8626
2.5571
2.5483
2.5377
2.5234
1.8815
1.8695
1.8571
1.8418
1.8295
1.7613
1.05
3.00
1.01
1.97
1.99
0.93
1.00
0.92
1.94
1.83
2.01
1.98
2.04
Chemical Shift (ppm)

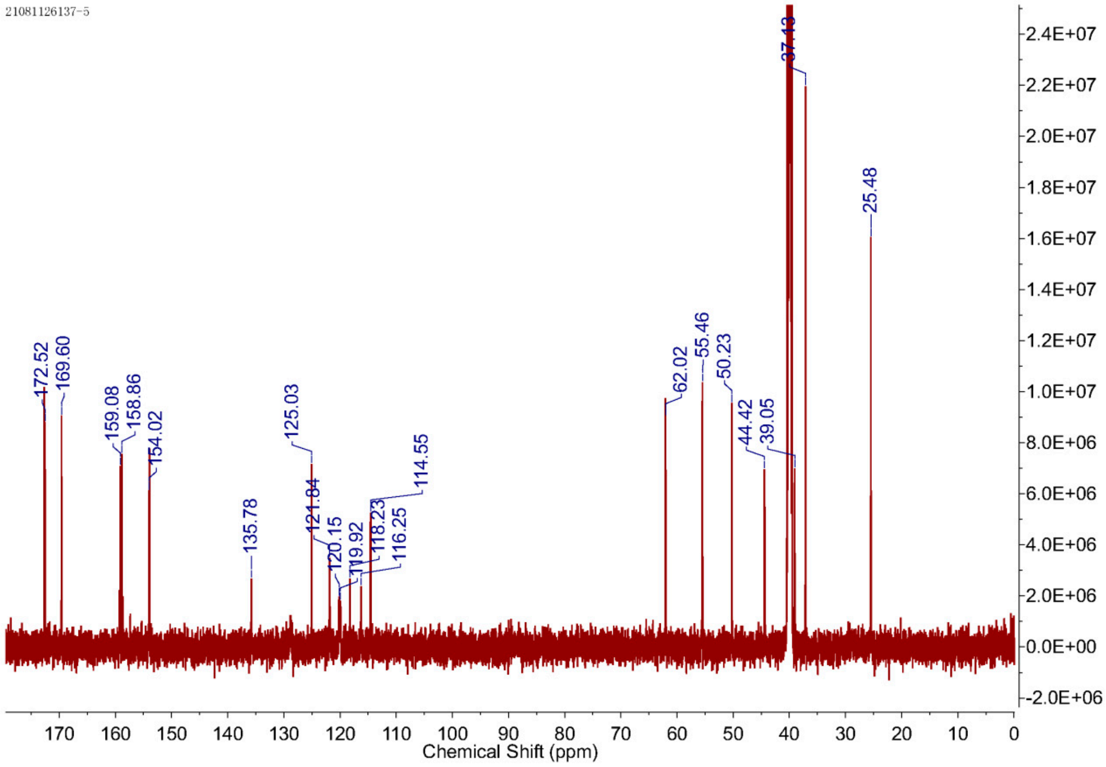
21081126137-5
172.52
169.60
159.08
158.86
154.02
135.78
125.03
121.84
120.15
119.92
118.23
116.25
114.55
62.02
55.46
50.23
44.42
39.05
25.48
Chemical Shift (ppm)

(CA1-a)·TFA

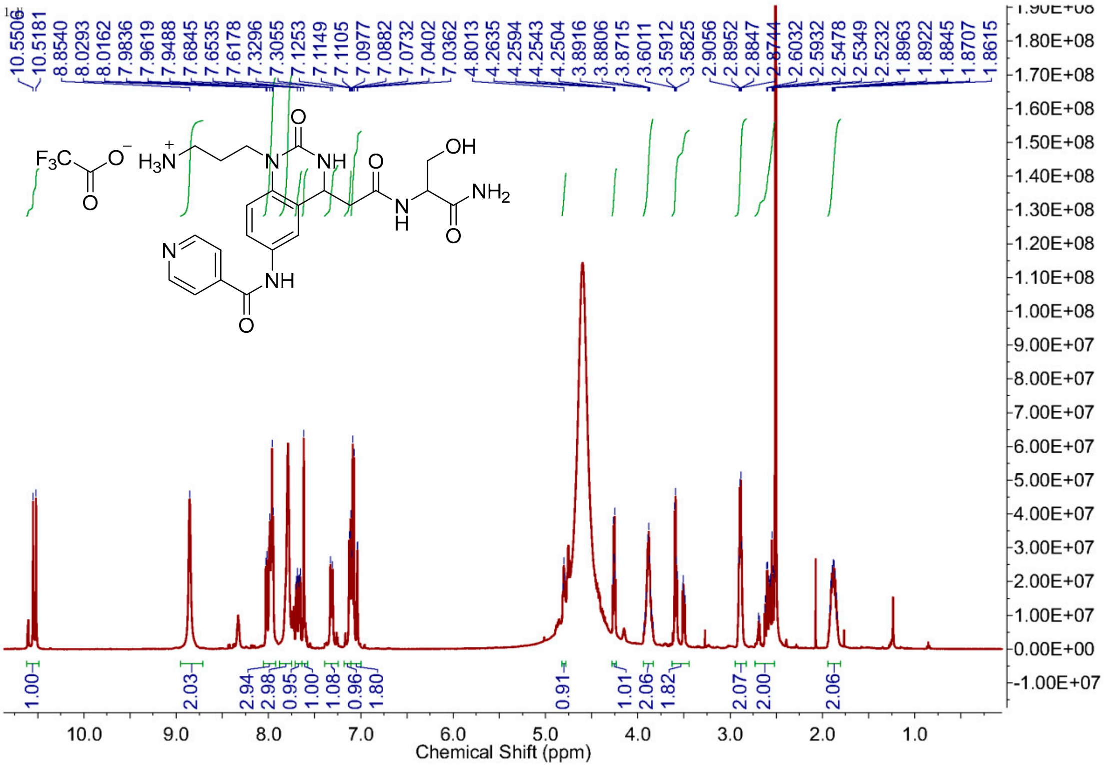
1-H
10.5506
10.5181
8.8540
8.0293
8.0162
7.9836
7.9619
7.9488
7.6845
7.6535
7.6178
7.3296
7.3055
7.1253
7.1149
7.1105
7.0977
7.0882
7.0732
7.0402
7.0362
4.8013
4.2635
4.2594
4.2543
4.2504
3.8916
3.8806
3.8715
3.6011
3.5912
3.5825
2.9056
2.8952
2.8847
2.8744
2.6032
2.5932
2.5478
2.5349
2.5232
1.8963
1.8922
1.8845
1.8707
1.8615
1.00
2.03
2.94
2.98
0.95
1.00
1.08
0.96
1.80
0.91
1.01
2.06
1.82
2.07
2.00
2.06
Chemical Shift (ppm)

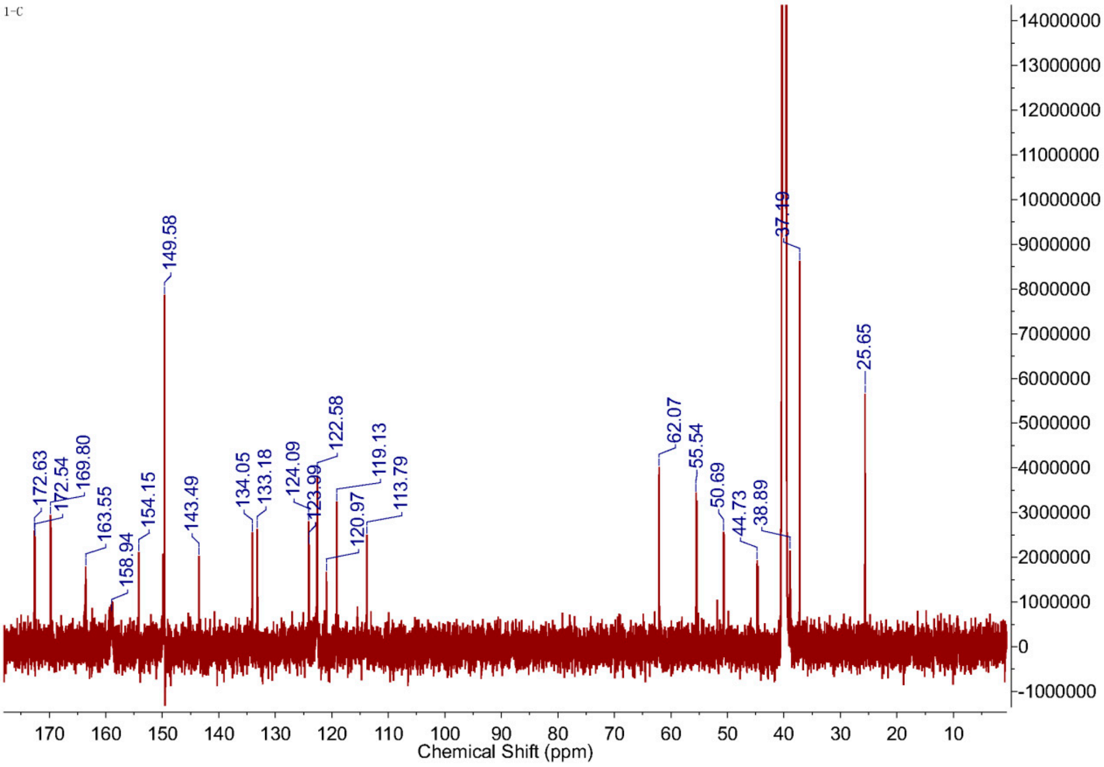
1-C
172.63
172.54
169.80
163.55
158.94
154.15
149.58
143.49
134.05
133.18
124.09
123.99
122.58
120.97
119.13
113.79
62.07
55.54
50.69
44.73
38.89
37.19
25.65
Chemical Shift (ppm)

(CA1-b)·TFA

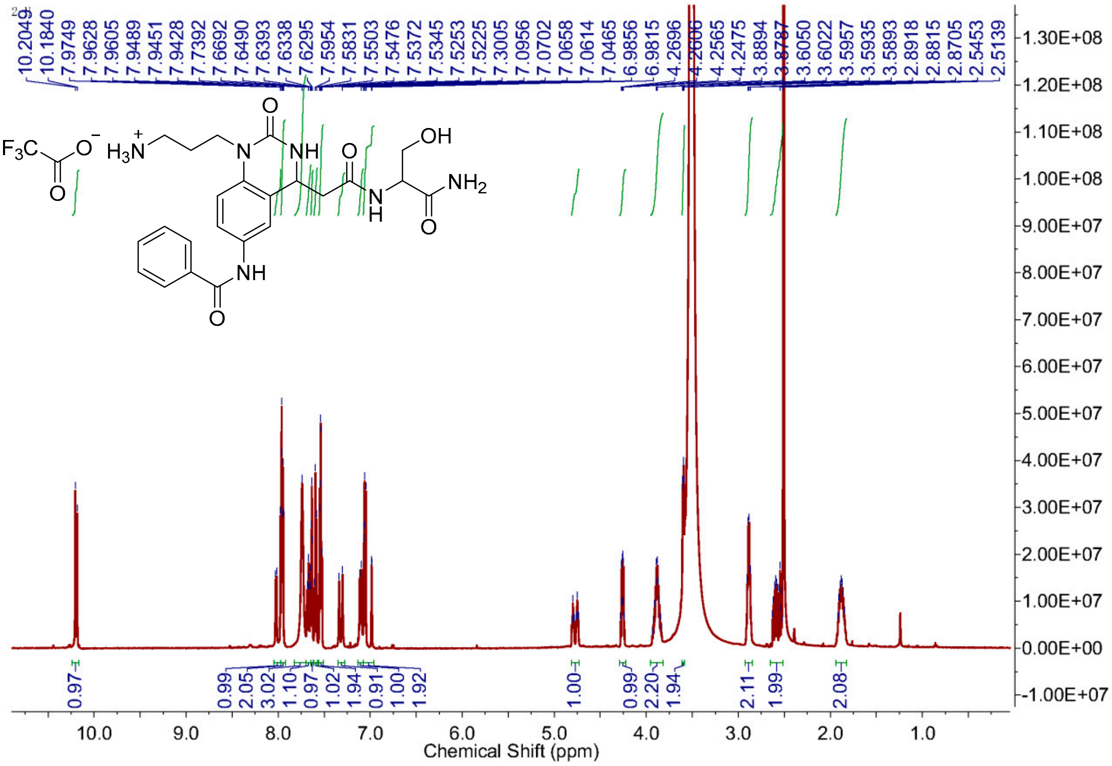
2-H
10.2049 10.1840 7.9749 7.9628 7.9605 7.9489 7.9451 7.9428 7.7392 7.6692 7.6490 7.6393 7.6338 7.6295 7.5954 7.5831 7.5503 7.5476 7.5372 7.5345 7.5253 7.5225 7.3005 7.0956 7.0702 7.0658 7.0614 7.0465 6.9856 6.9815 4.2696 4.2606 4.2565 4.2475 3.8894 3.8787 3.6050 3.6022 3.5957 3.5935 3.5893 2.8918 2.8815 2.8705 2.5453 2.5139
0.97 0.99 2.05 3.02 1.10 0.97 1.02 1.94 0.91 1.00 1.92 1.00 0.99 2.20 1.94 2.11 1.99 2.08
Chemical Shift (ppm)

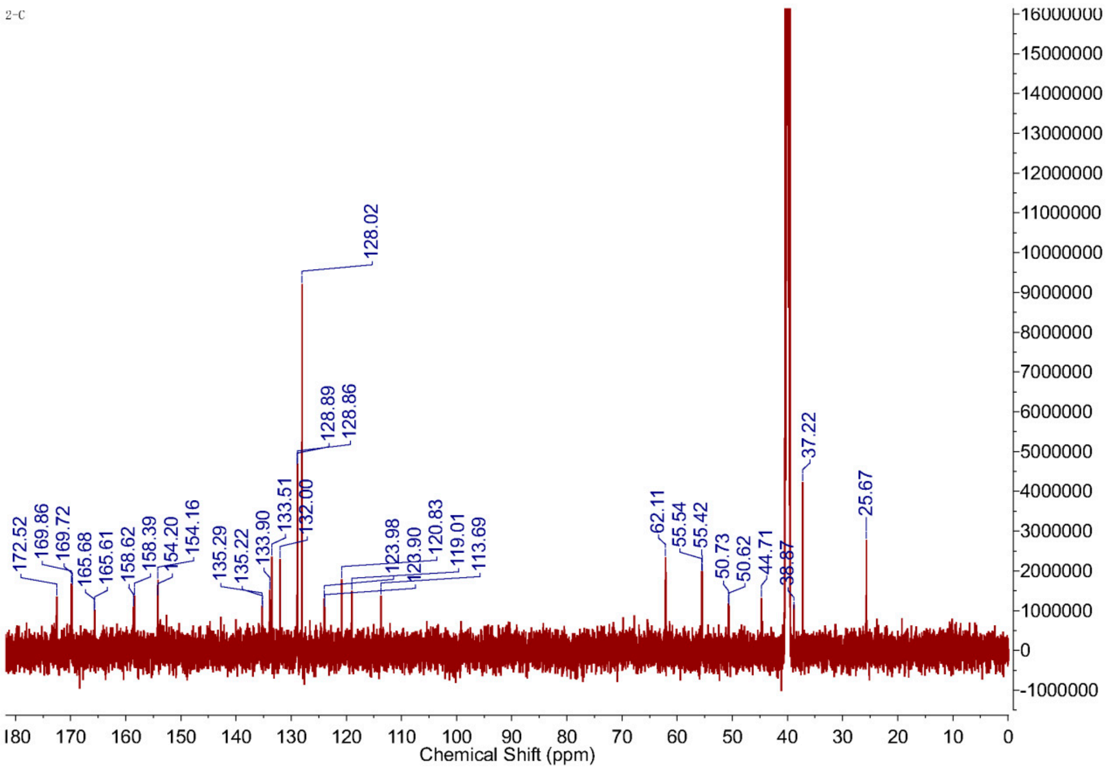
2-C
172.52 169.86 169.72 165.68 165.61 158.62 158.39 154.20 154.16 135.29 135.22 133.90 133.51 132.00 128.89 128.86 128.02 123.98 123.90 120.83 119.01 113.69 62.11 55.54 55.42 50.73 50.62 44.71 38.87 37.22 25.67
Chemical Shift (ppm)

(CA1-c)·TFA

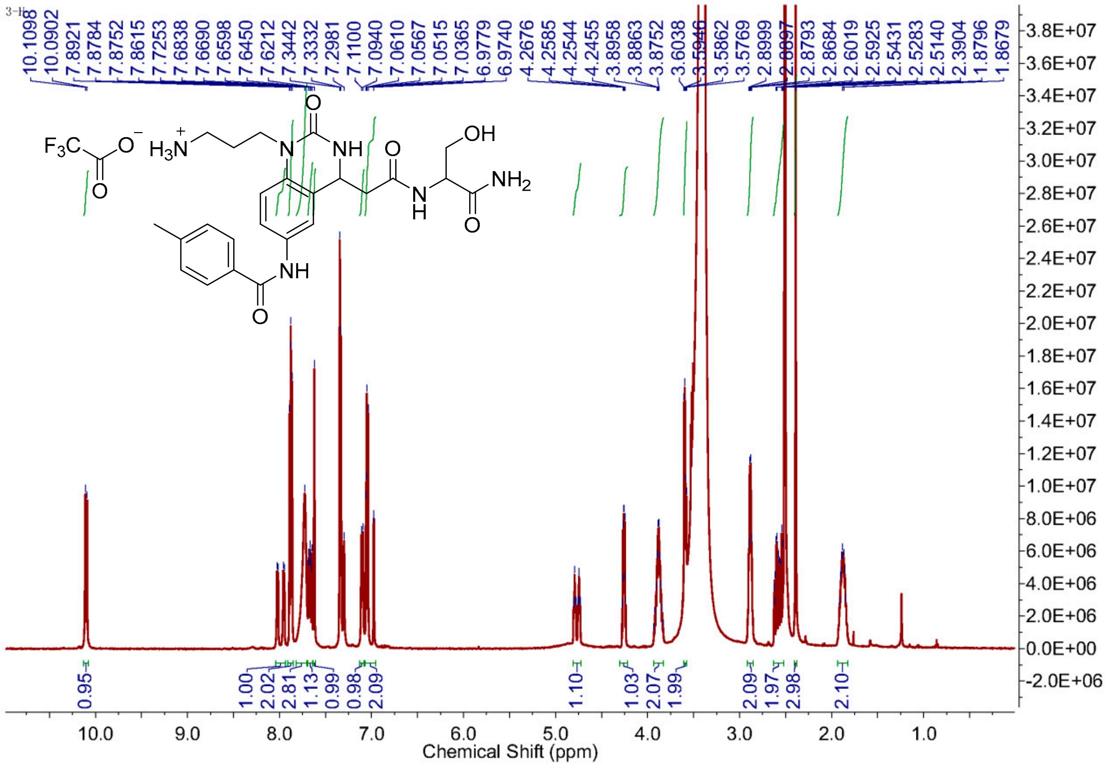

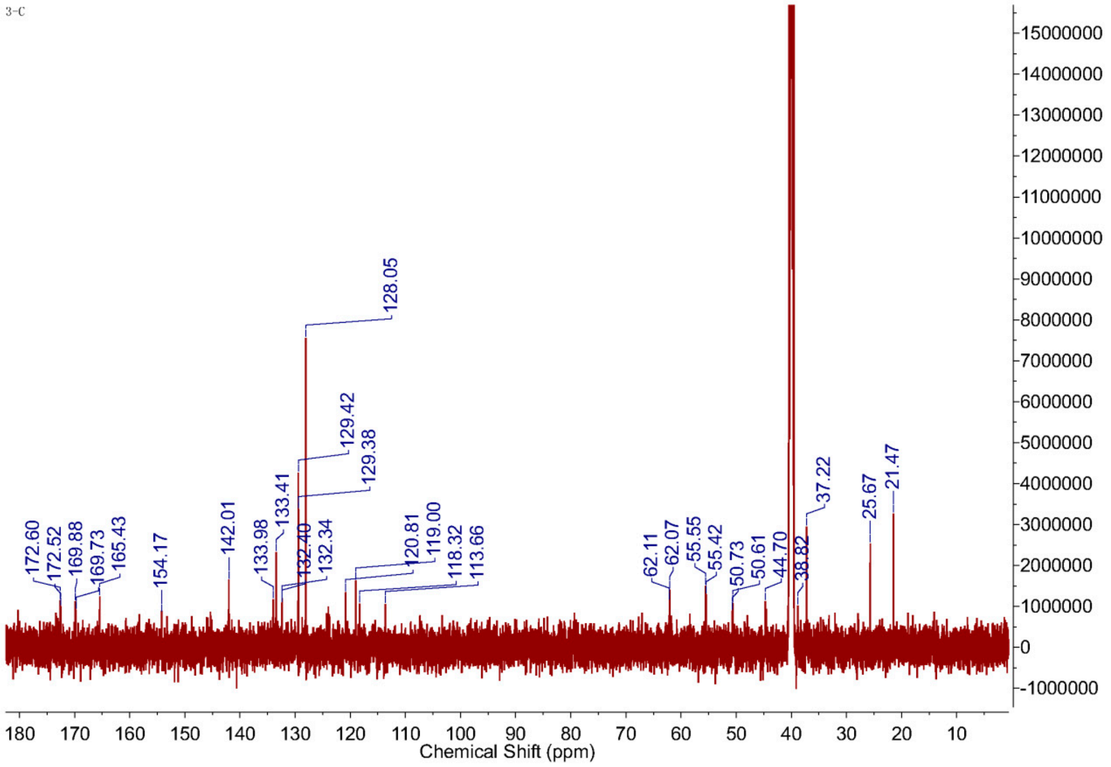

(CA1-d)·TFA

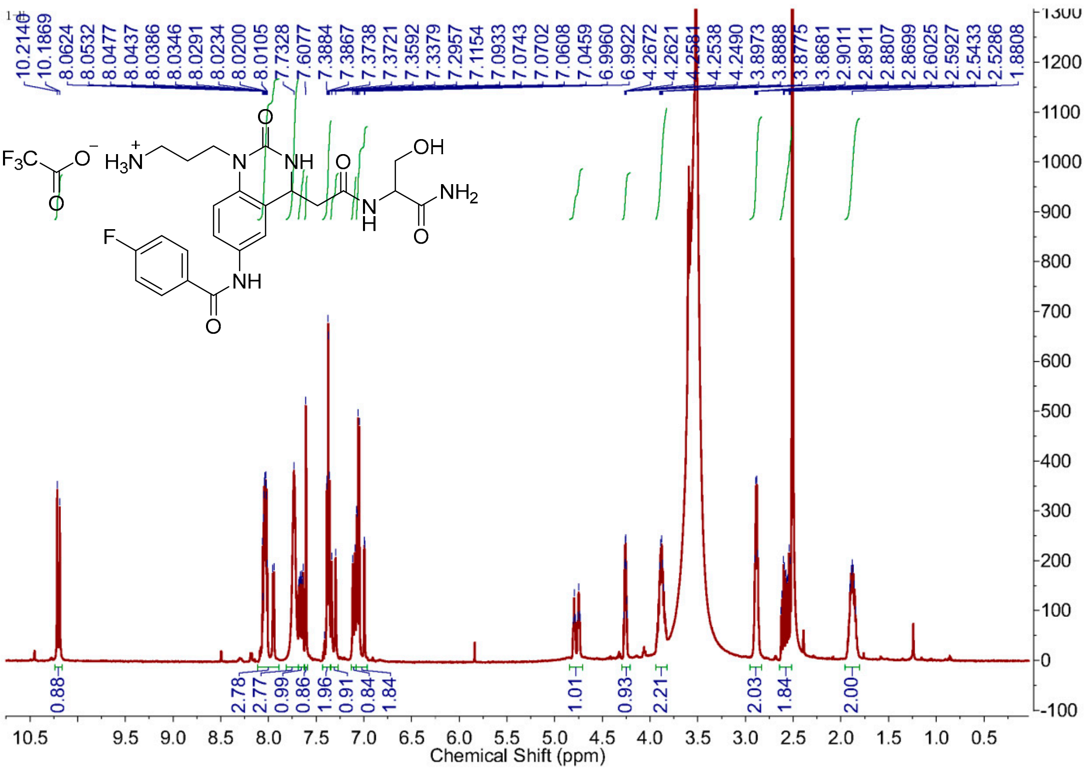
10.2140
10.1869
8.0624
8.0532
8.0477
8.0437
8.0386
8.0346
8.0291
8.0234
8.0200
8.0105
7.7328
7.6077
7.3884
7.3867
7.3738
7.3721
7.3592
7.3379
7.2957
7.1154
7.0933
7.0743
7.0702
7.0608
7.0459
6.9960
6.9922
4.2672
4.2621
4.2581
4.2538
4.2490
3.8973
3.8888
3.8775
3.8681
2.9011
2.8911
2.8807
2.8699
2.6025
2.5927
2.5433
2.5286
1.8808
0.88
2.78
2.77
0.99
0.86
1.96
0.91
0.84
1.84
1.01
0.93
2.21
2.03
1.84
2.00
Chemical Shift (ppm)

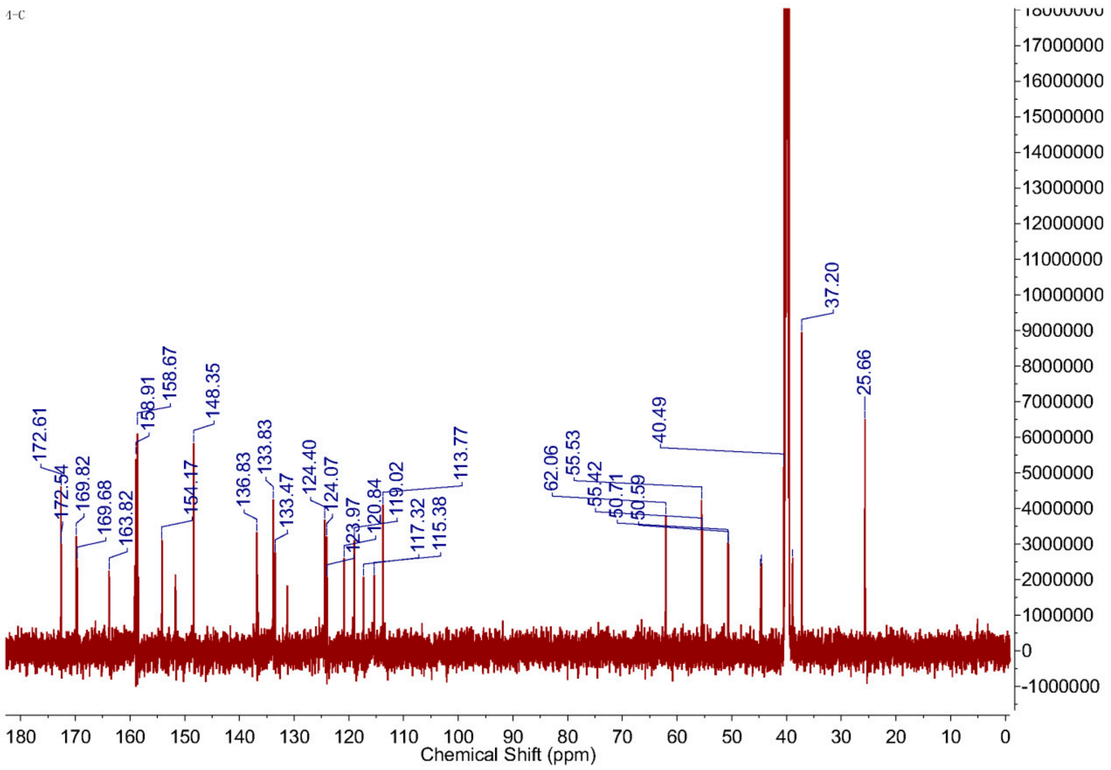
172.61
172.54
169.82
169.68
163.82
158.91
158.67
154.17
148.35
136.83
133.83
133.47
124.40
124.07
123.97
120.84
119.02
117.32
115.38
113.77
62.06
55.53
55.42
50.71
50.59
40.49
37.20
25.66
Chemical Shift (ppm)

(CA1-e)·TFA

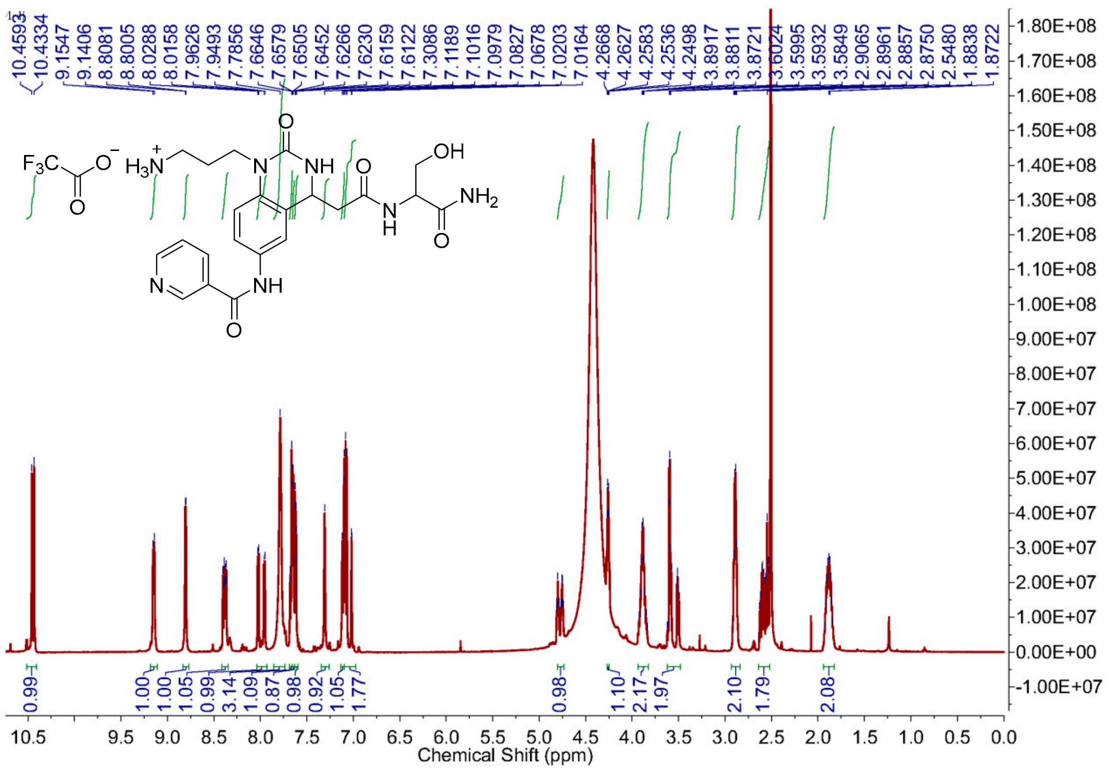

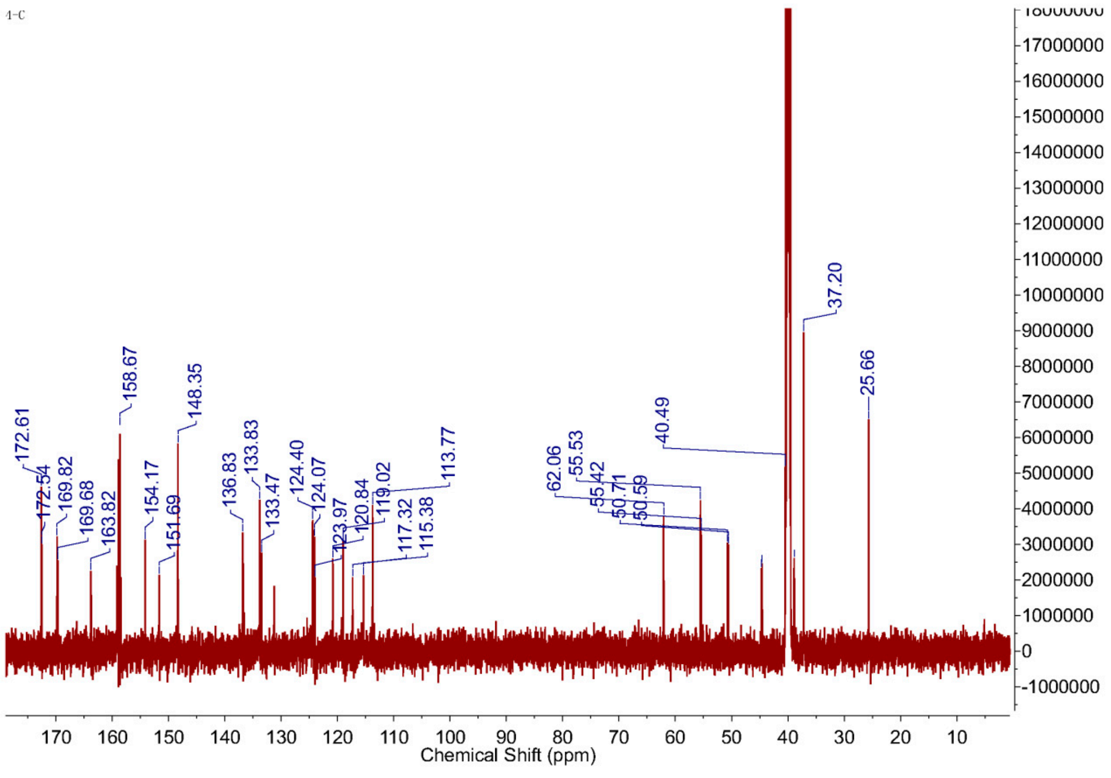

(CA1-f)·TFA

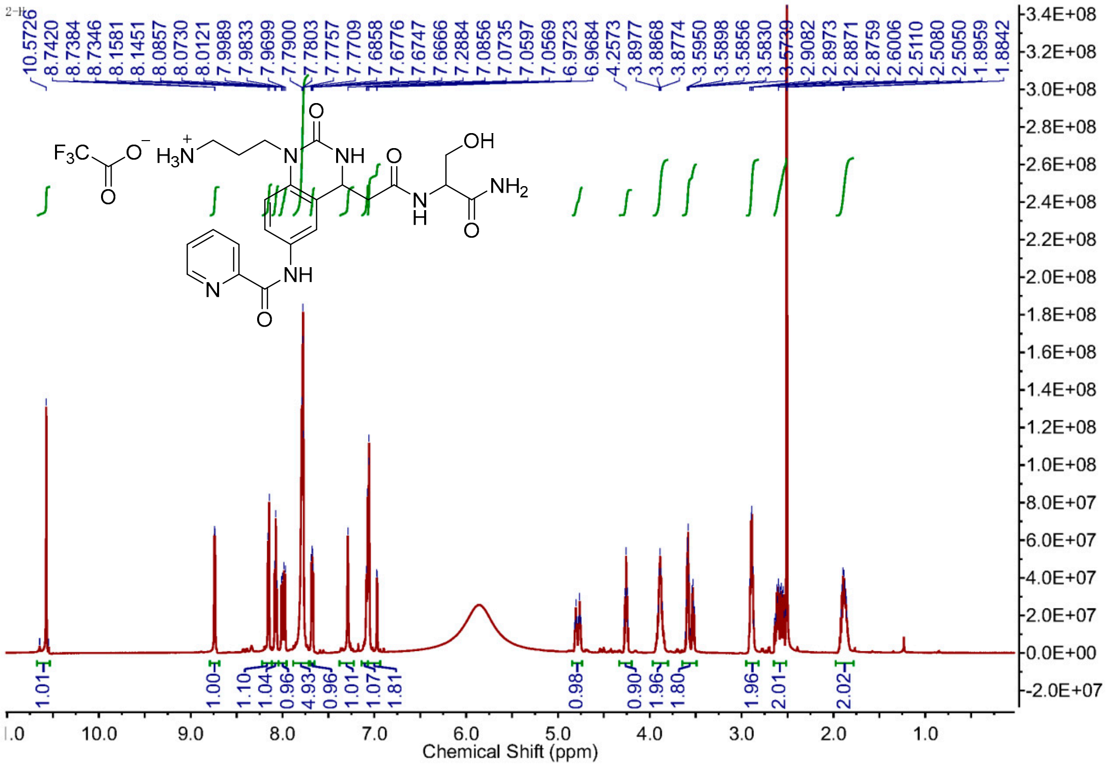
2-H
10.5726
8.7420
8.7384
8.7346
8.1581
8.1451
8.0857
8.0730
8.0121
7.9989
7.9833
7.9699
7.7900
7.7803
7.7757
7.7709
7.6858
7.6776
7.6747
7.6666
7.2884
7.0856
7.0735
7.0597
7.0569
6.9723
6.9684
4.2573
3.8977
3.8868
3.8774
3.5950
3.5898
3.5856
3.5830
2.9082
2.8973
2.8871
2.8759
2.6006
2.5110
2.5080
2.5050
1.8959
1.8842
F_3C
O
H_3N
N
NH
OH
NH2
N
H
NH
N
O
1.01
1.00
1.10
1.04
0.96
4.93
0.96
1.01
1.07
1.81
0.98
0.90
1.96
1.80
1.96
2.01
2.02
Chemical Shift (ppm)

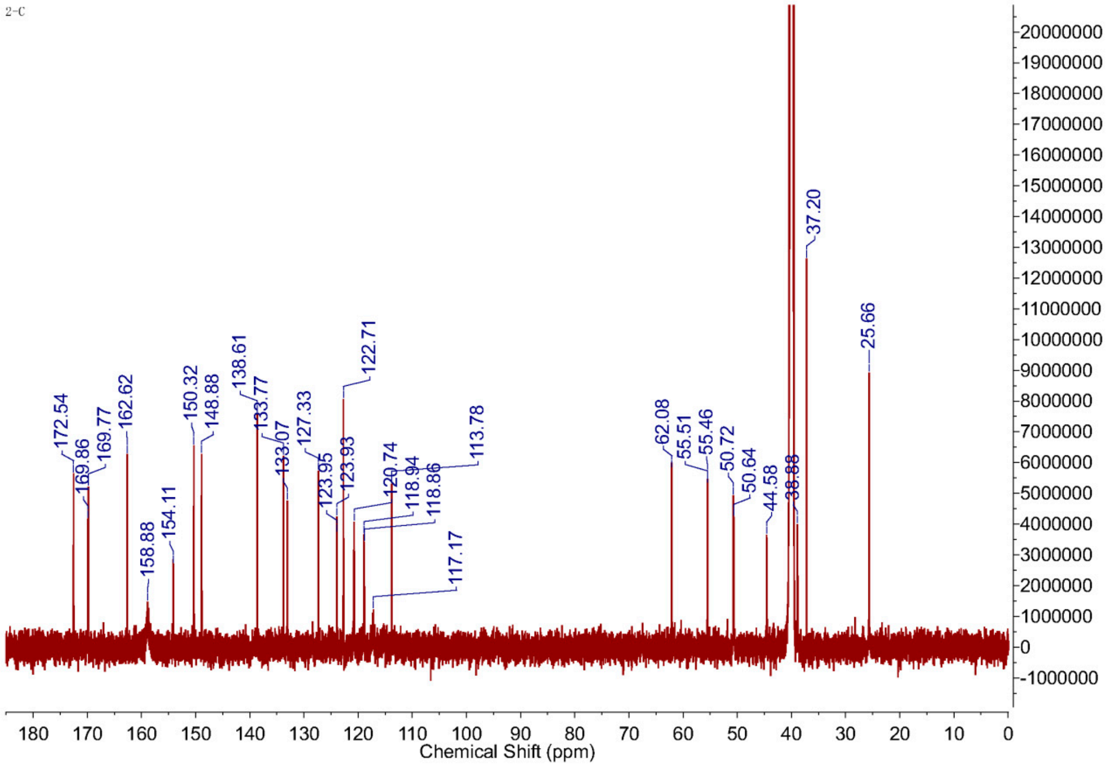
2-C
172.54
169.86
169.77
162.62
158.88
154.11
150.32
148.88
138.61
133.77
133.07
127.33
123.95
123.93
122.71
120.74
118.94
118.86
117.17
113.78
62.08
55.51
55.46
50.72
50.64
44.58
38.88
37.20
25.66
Chemical Shift (ppm)

(CA1-g)·TFA

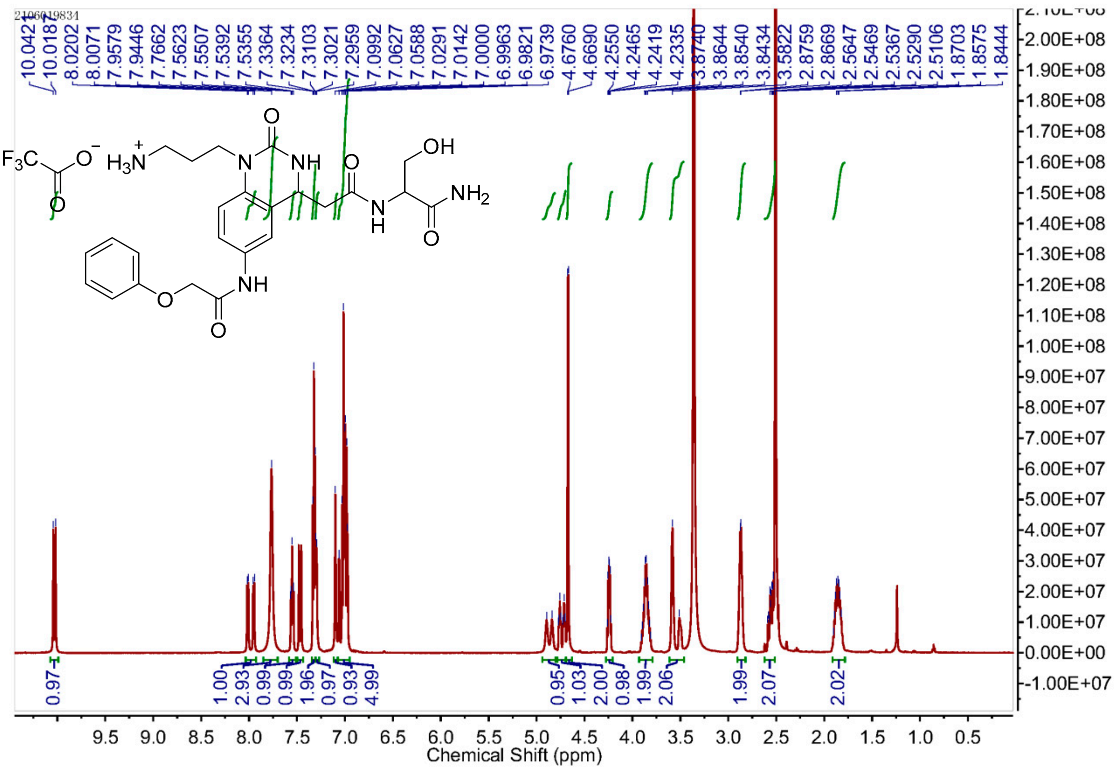

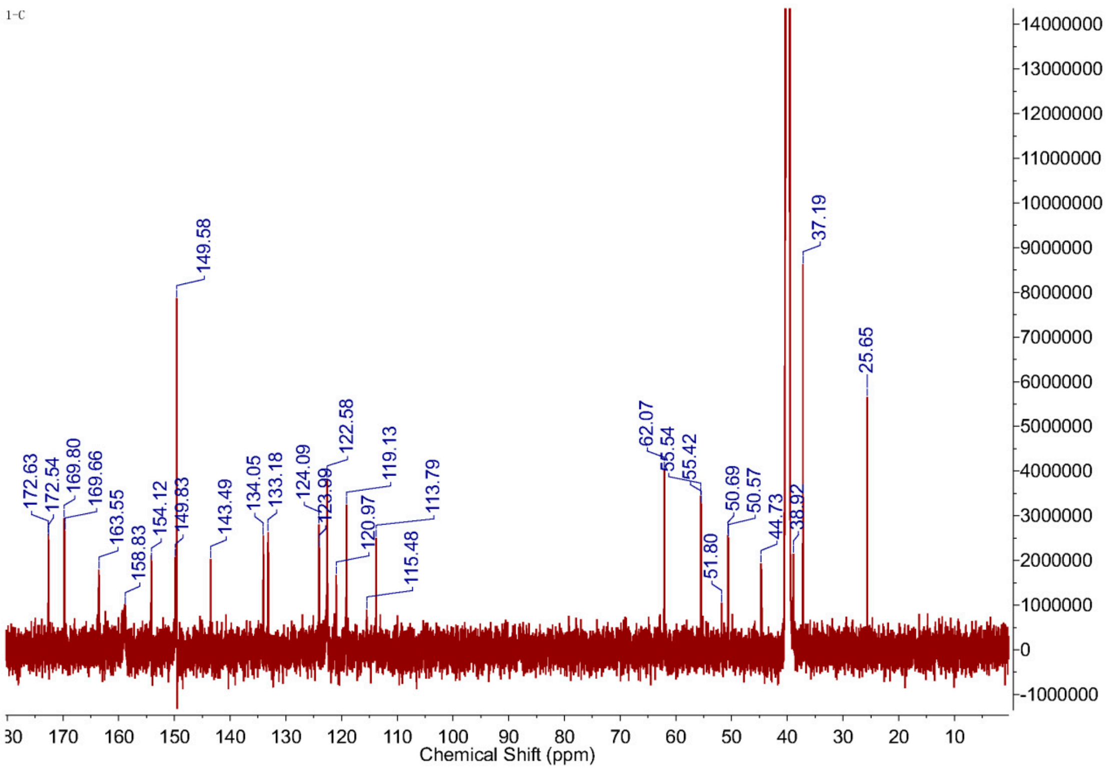

(CA1-h)·TFA

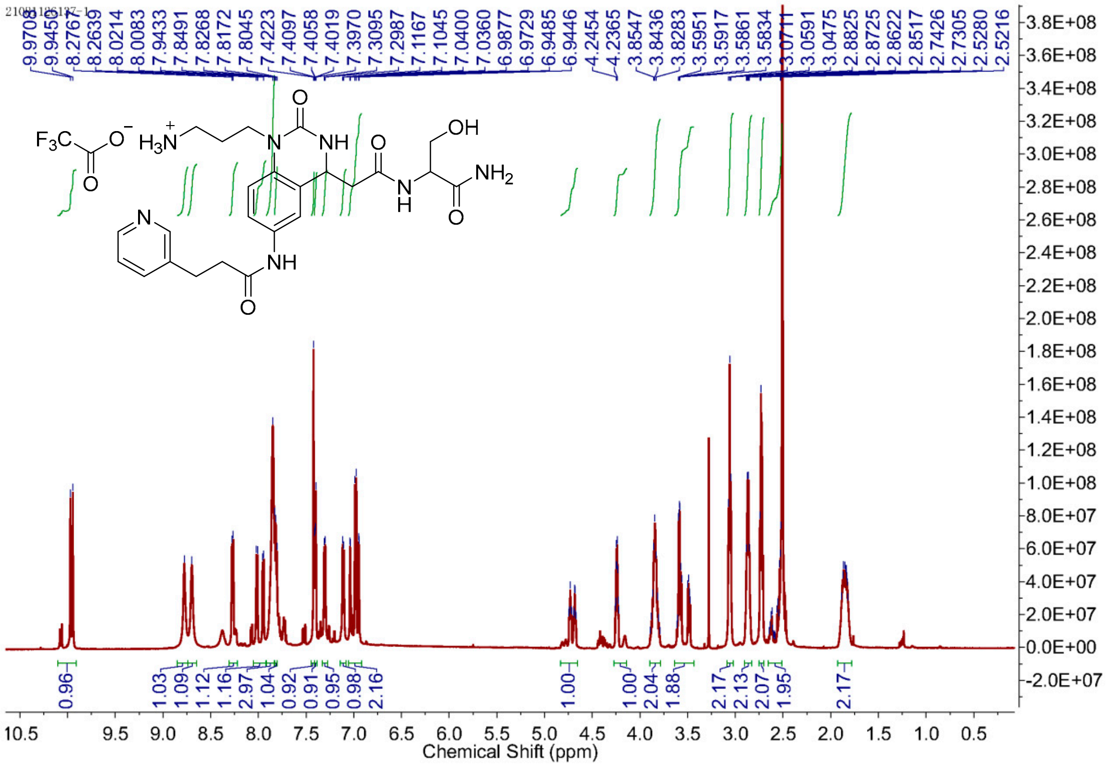
21081126137-1
9.9708
9.9450
8.2767
8.2639
8.0214
8.0083
7.9433
7.8491
7.8268
7.8172
7.8045
7.4223
7.4097
7.4058
7.4019
7.3970
7.3095
7.2987
7.1167
7.1045
7.0400
7.0360
6.9877
6.9729
6.9485
6.9446
4.2454
4.2365
3.8547
3.8436
3.8283
3.5951
3.5917
3.5861
3.5834
3.0711
3.0591
3.0475
2.8825
2.8725
2.8622
2.8517
2.7426
2.7305
2.5280
2.5216
0.96
1.03
1.09
1.12
1.16
2.97
1.04
0.92
0.91
0.95
0.98
2.16
1.00
1.00
2.04
1.88
2.17
2.13
2.07
1.95
2.17
Chemical Shift (ppm)

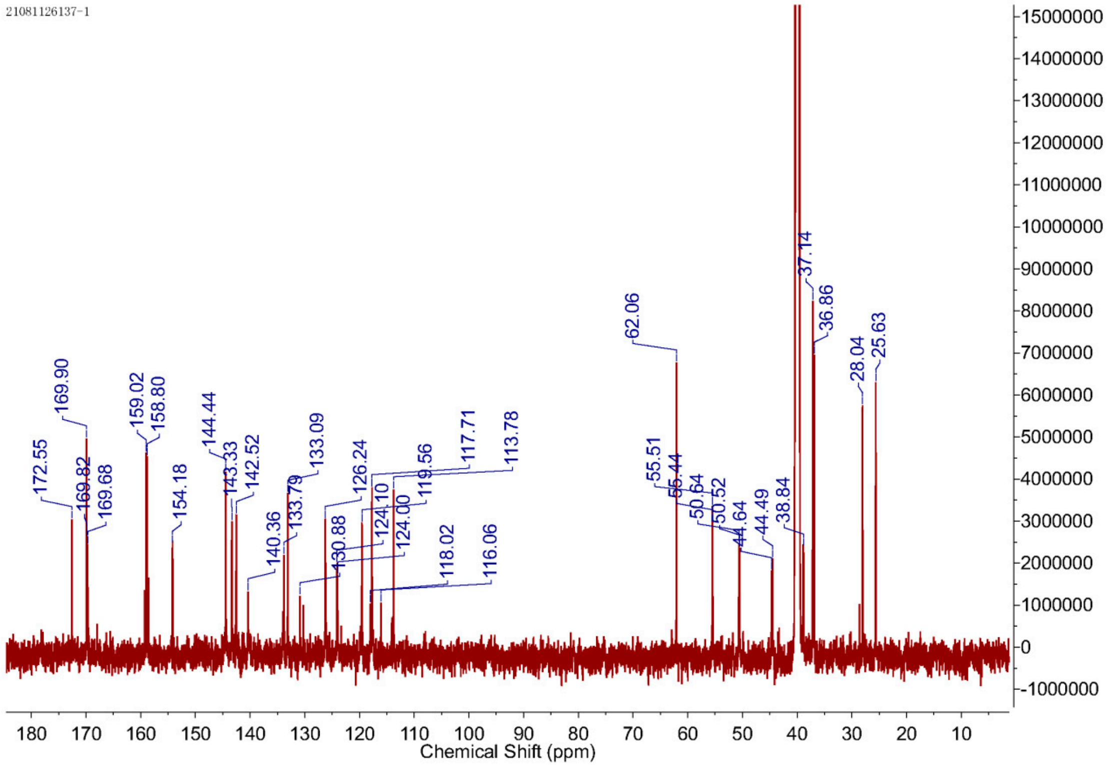
21081126137-1
172.55
169.90
169.82
169.68
159.02
158.80
154.18
144.44
143.33
142.52
140.36
133.79
133.09
130.88
126.24
124.10
124.00
119.56
118.02
117.71
116.06
113.78
62.06
55.51
55.44
50.64
50.52
44.64
44.49
38.84
37.14
36.86
28.04
25.63
Chemical Shift (ppm)

(CA1-i)·TFA

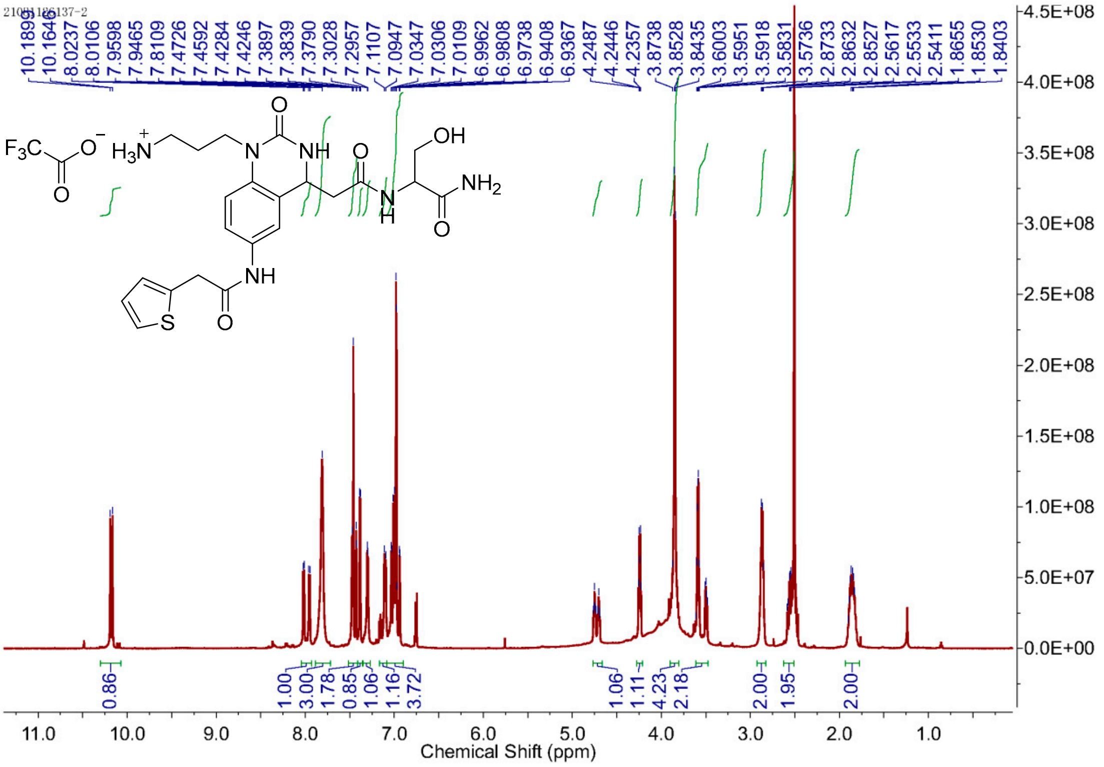
21081126137-2
10.1899
10.1646
8.0237
8.0106
7.9598
7.9465
7.8109
7.4726
7.4592
7.4284
7.4246
7.3897
7.3839
7.3790
7.3028
7.2957
7.1107
7.0947
7.0347
7.0306
7.0109
6.9962
6.9808
6.9738
6.9408
6.9367
4.2487
4.2446
4.2357
3.8738
3.8528
3.8435
3.6003
3.5951
3.5918
3.5831
3.5736
2.8733
2.8632
2.8527
2.5617
2.5533
2.5411
1.8655
1.8530
1.8403
F3C
O−
O
H3N+
N
NH
O
O
N
H
OH
NH2
O
NH
S
O
0.86
1.00
3.00
1.78
0.85
1.06
1.16
3.72
1.06
1.11
4.23
2.18
2.00
1.95
2.00
Chemical Shift (ppm)

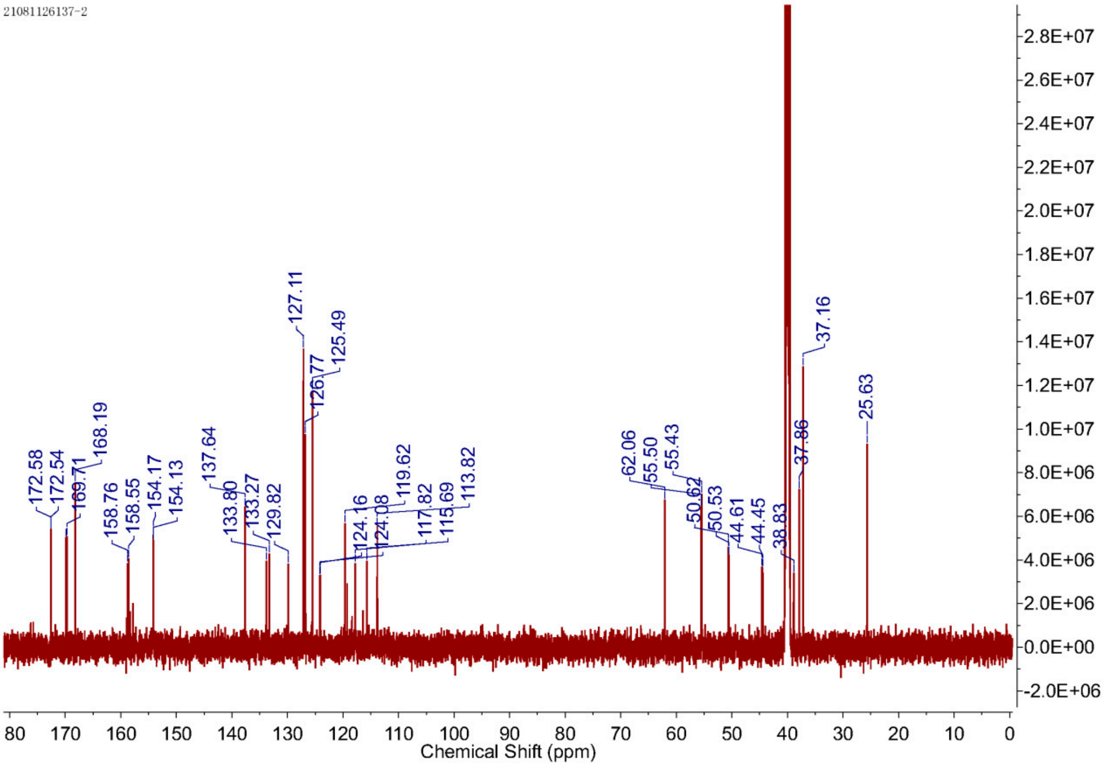
21081126137-2
172.58
172.54
169.71
168.19
158.76
158.55
154.17
154.13
137.64
133.80
133.27
129.82
127.11
126.77
125.49
124.16
124.08
119.62
117.82
115.69
113.82
62.06
55.50
55.43
50.62
50.53
44.61
44.45
38.83
37.86
37.16
25.63
Chemical Shift (ppm)

(CA1-j)·TFA

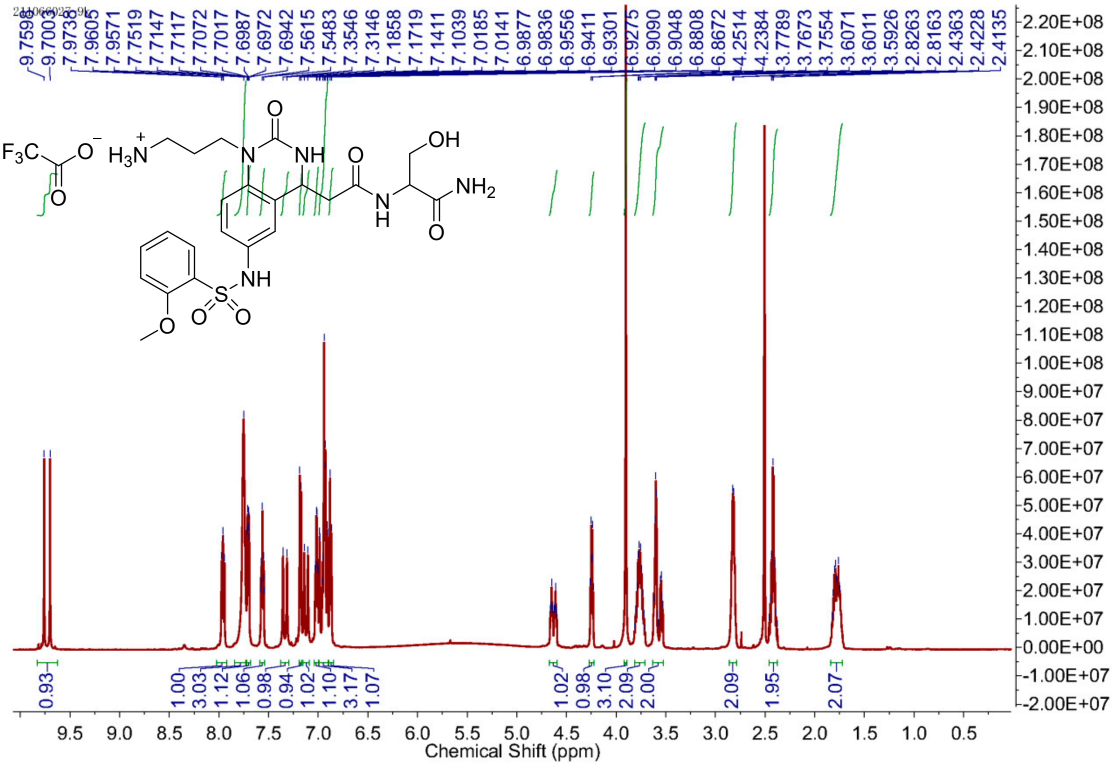
211066027-9k
9.7593
9.7003
7.9738
7.9605
7.9571
7.7519
7.7147
7.7117
7.7072
7.7017
7.6987
7.6972
7.6942
7.5615
7.5483
7.3546
7.3146
7.1858
7.1719
7.1411
7.1039
7.0185
7.0141
6.9877
6.9836
6.9556
6.9411
6.9301
6.9275
6.9090
6.9048
6.8808
6.8672
4.2514
4.2384
3.7789
3.7673
3.7554
3.6071
3.6011
3.5926
2.8263
2.8163
2.4363
2.4228
2.4135
0.93
1.00
3.03
1.12
1.06
0.98
0.94
1.02
1.10
3.17
1.07
1.02
0.98
3.10
2.09
2.00
2.09
1.95
2.07
Chemical Shift (ppm)

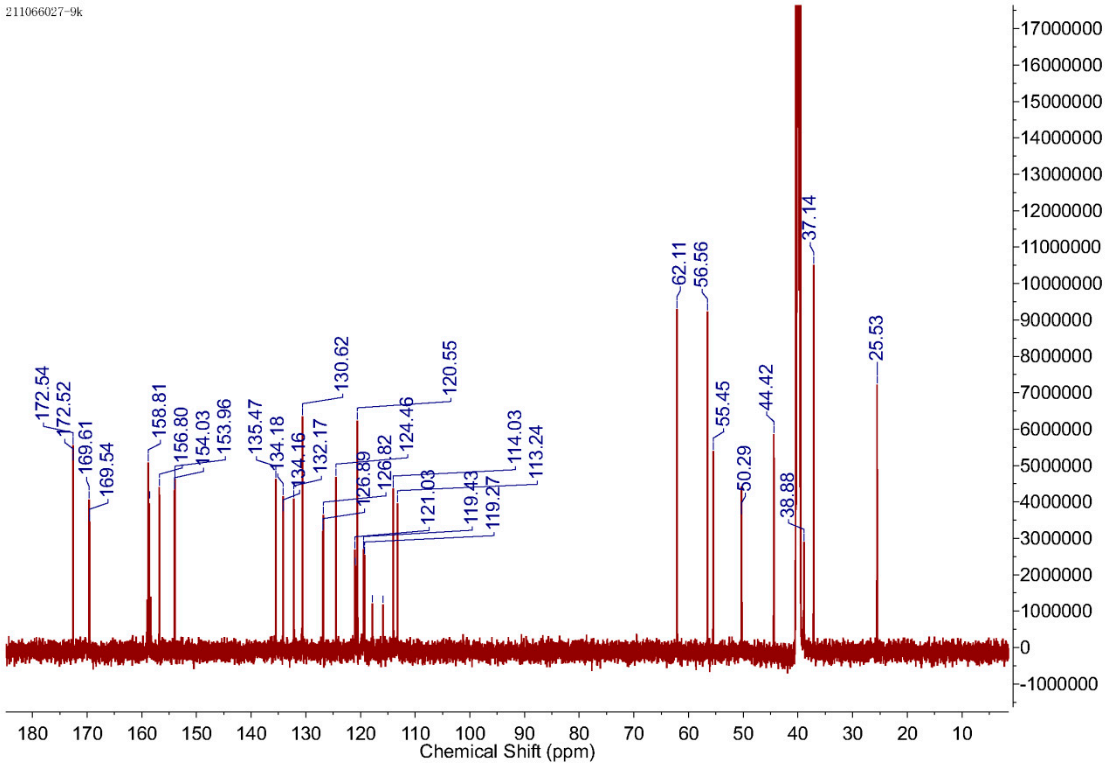
211066027-9k
172.54
172.52
169.61
169.54
158.81
156.80
154.03
153.96
135.47
134.18
134.16
132.17
130.62
126.89
126.82
124.46
121.03
120.55
119.43
119.27
114.03
113.24
62.11
56.56
55.45
50.29
44.42
38.88
37.14
25.53
Chemical Shift (ppm)

(CA3)·TFA

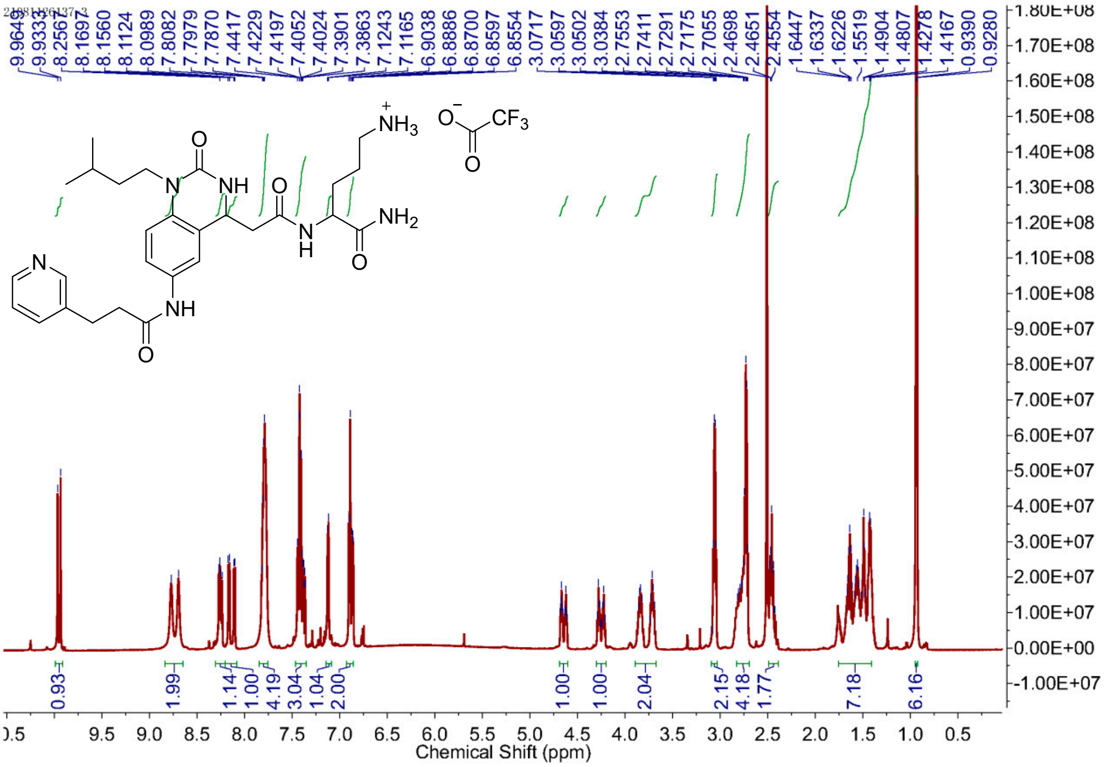

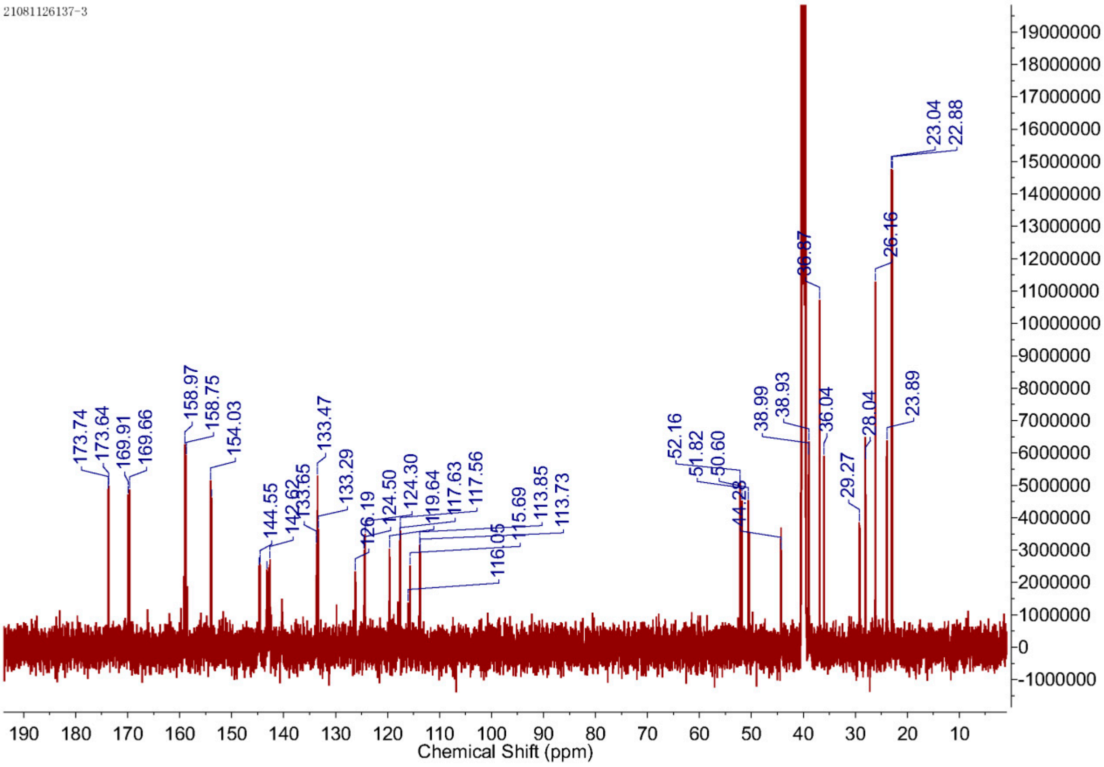

CA4

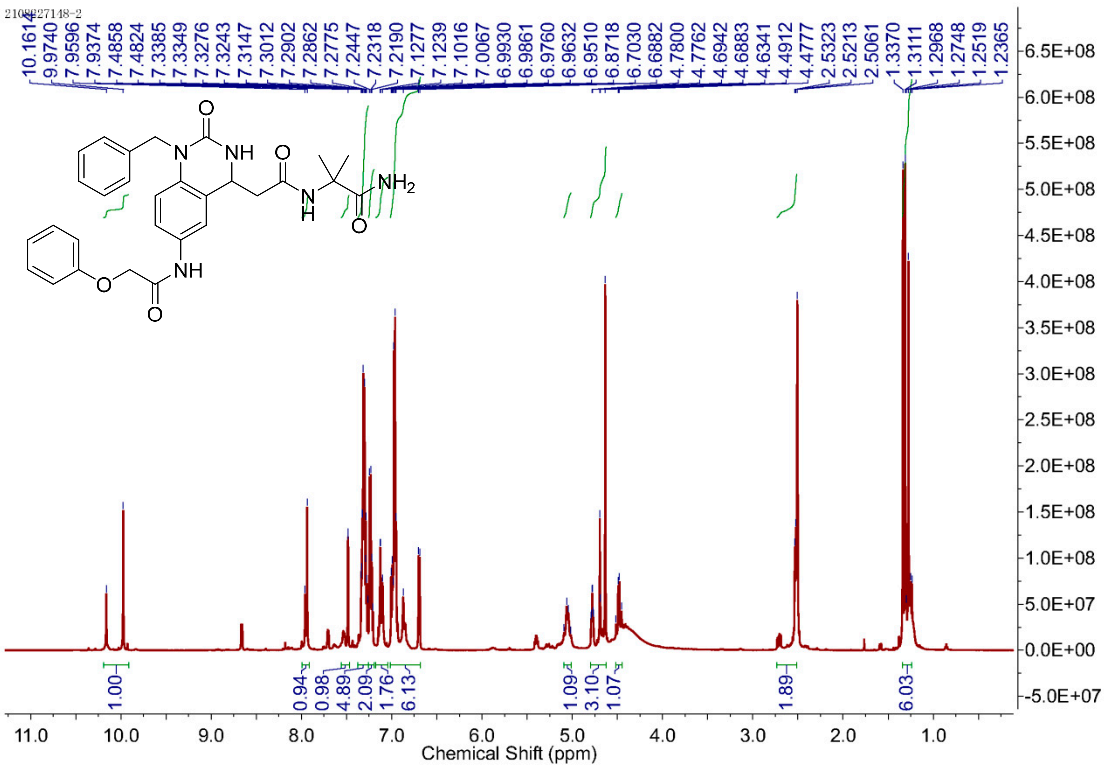
2108227148-2
10.1614
9.9740
7.9596
7.9374
7.4858
7.4824
7.3385
7.3349
7.3276
7.3243
7.3147
7.3012
7.2902
7.2862
7.2775
7.2447
7.2318
7.2190
7.1277
7.1239
7.1016
7.0067
6.9930
6.9861
6.9760
6.9632
6.9510
6.8718
6.7030
6.6882
4.7800
4.7762
4.6942
4.6883
4.6341
4.4912
4.4777
2.5323
2.5213
2.5061
1.3370
1.3111
1.2968
1.2748
1.2519
1.2365
NH
NH
NH2
N
H
O
O
O
O
N
1.00
0.94
0.98
4.89
2.09
1.76
6.13
1.09
3.10
1.07
1.89
6.03
Chemical Shift (ppm)

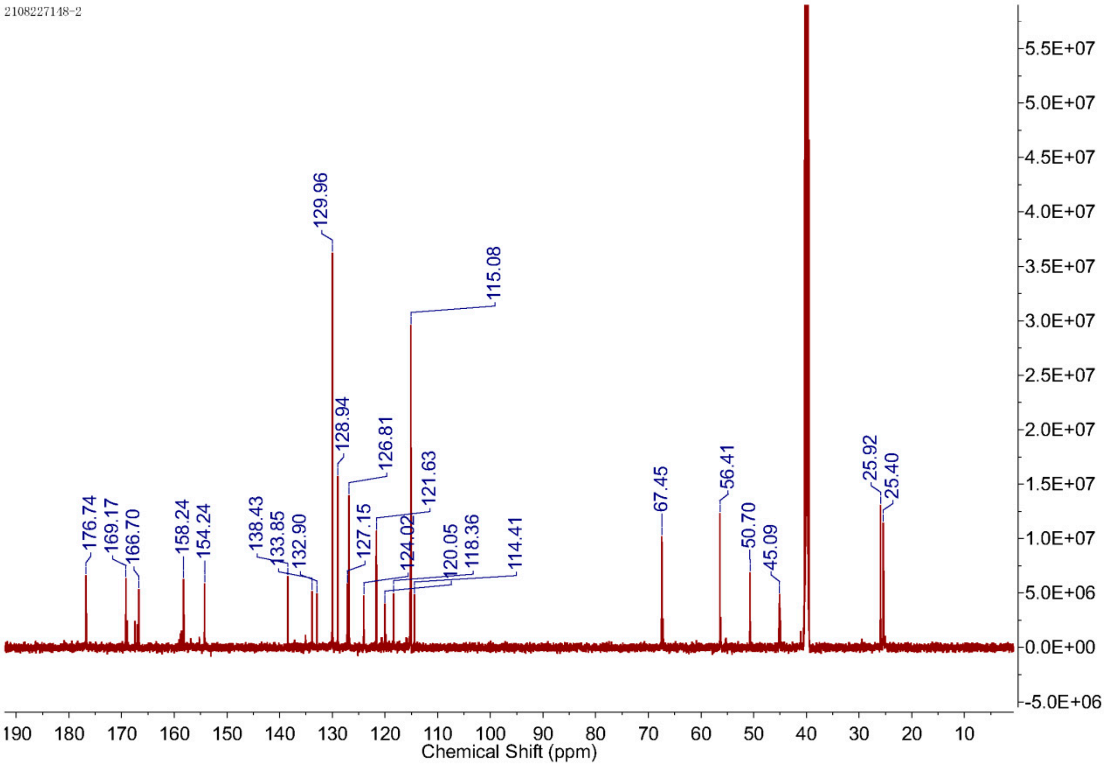
2108227148-2
176.74
169.17
166.70
158.24
154.24
138.43
133.85
132.90
129.96
128.94
127.15
126.81
124.02
121.63
120.05
118.36
115.08
114.41
67.45
56.41
50.70
45.09
25.92
25.40
Chemical Shift (ppm)

(CA5)·TFA

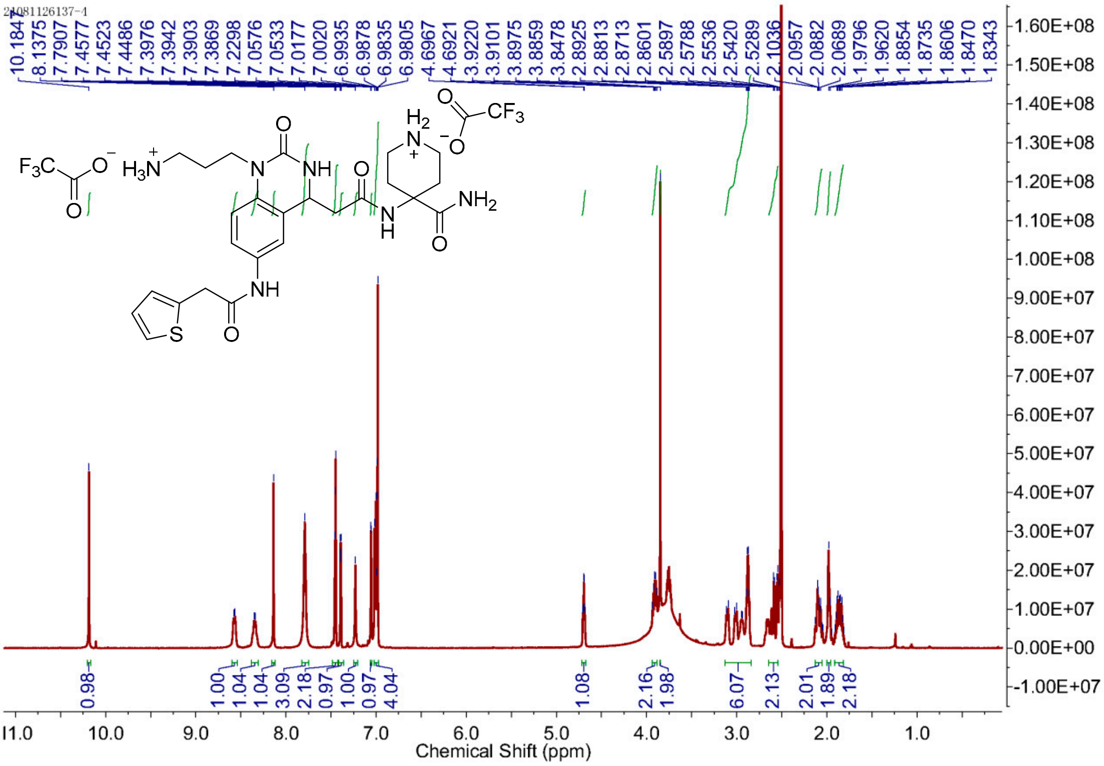

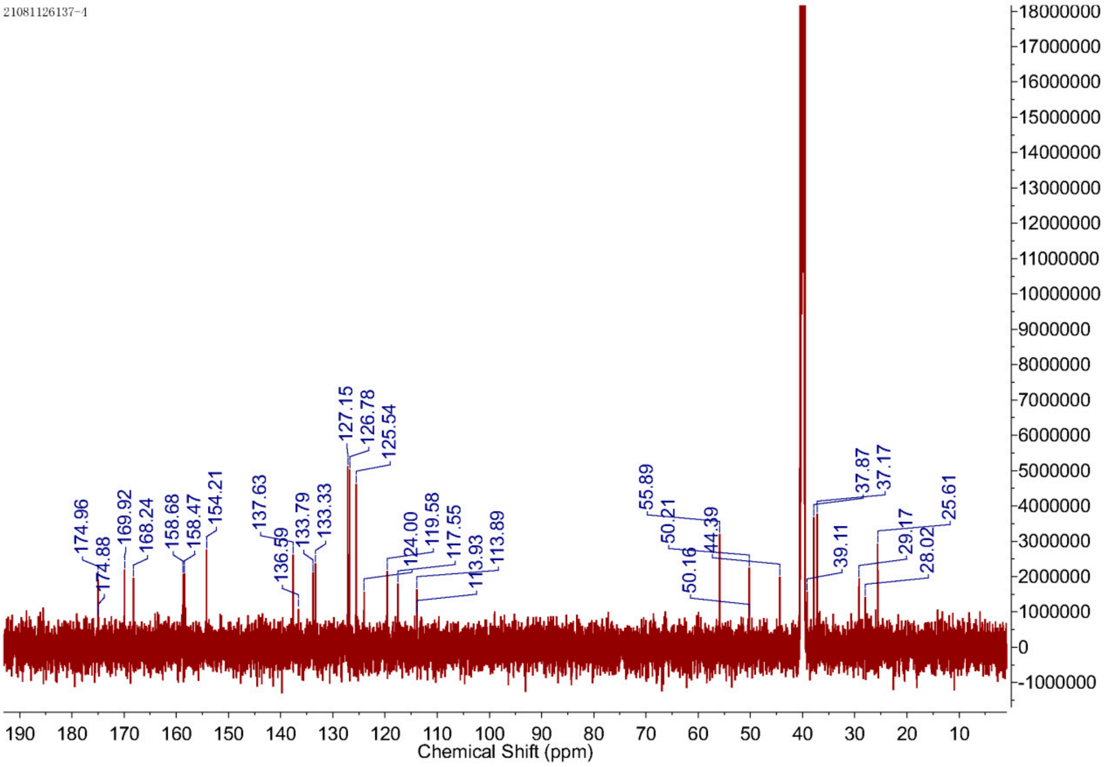

CA6-a

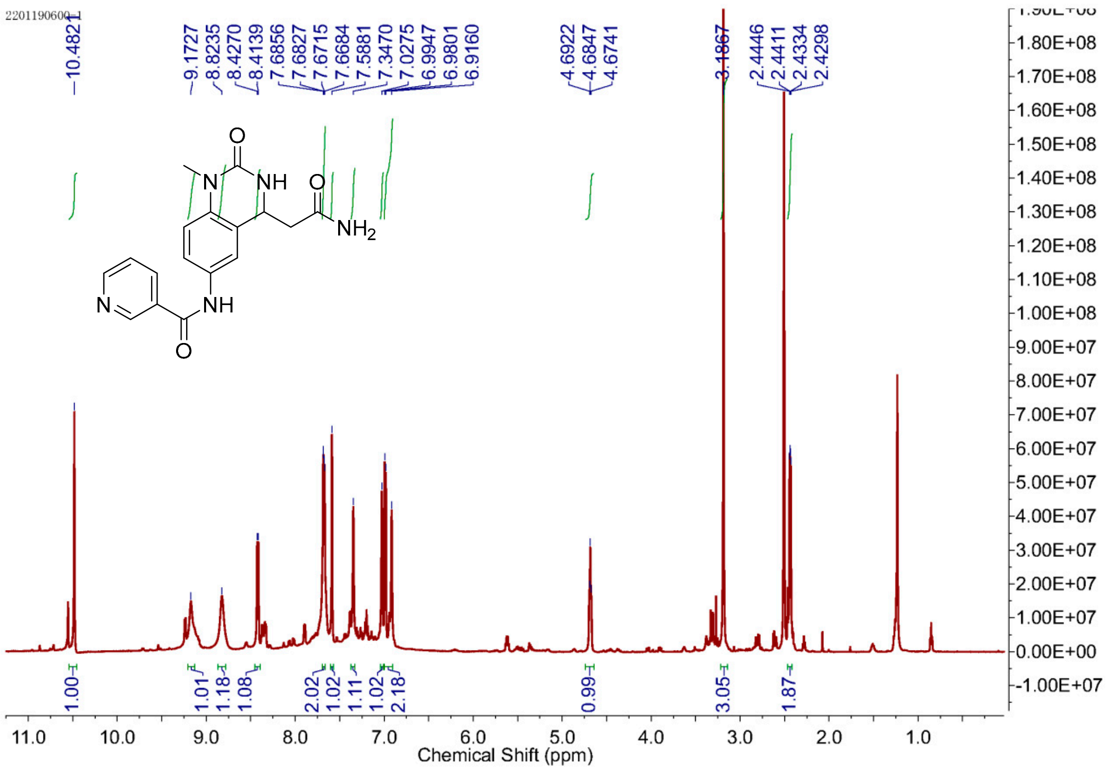
2201190600-1
10.4821
9.1727
8.8235
8.4270
8.4139
7.6856
7.6827
7.6715
7.6684
7.5881
7.3470
7.0275
6.9947
6.9801
6.9160
4.6922
4.6847
4.6741
3.1867
2.4446
2.4411
2.4334
2.4298
O
N
NH
O
NH2
N
NH
O
1.00
1.01
1.18
1.08
2.02
1.02
1.11
1.02
2.18
0.99
3.05
1.87
1.80E+08
1.70E+08
1.60E+08
1.50E+08
1.40E+08
1.30E+08
1.20E+08
1.10E+08
1.00E+08
9.00E+07
8.00E+07
7.00E+07
6.00E+07
5.00E+07
4.00E+07
3.00E+07
2.00E+07
1.00E+07
0.00E+00
-1.00E+07
11.0 10.0 9.0 8.0 7.0 6.0 5.0 4.0 3.0 2.0 1.0
Chemical Shift (ppm)

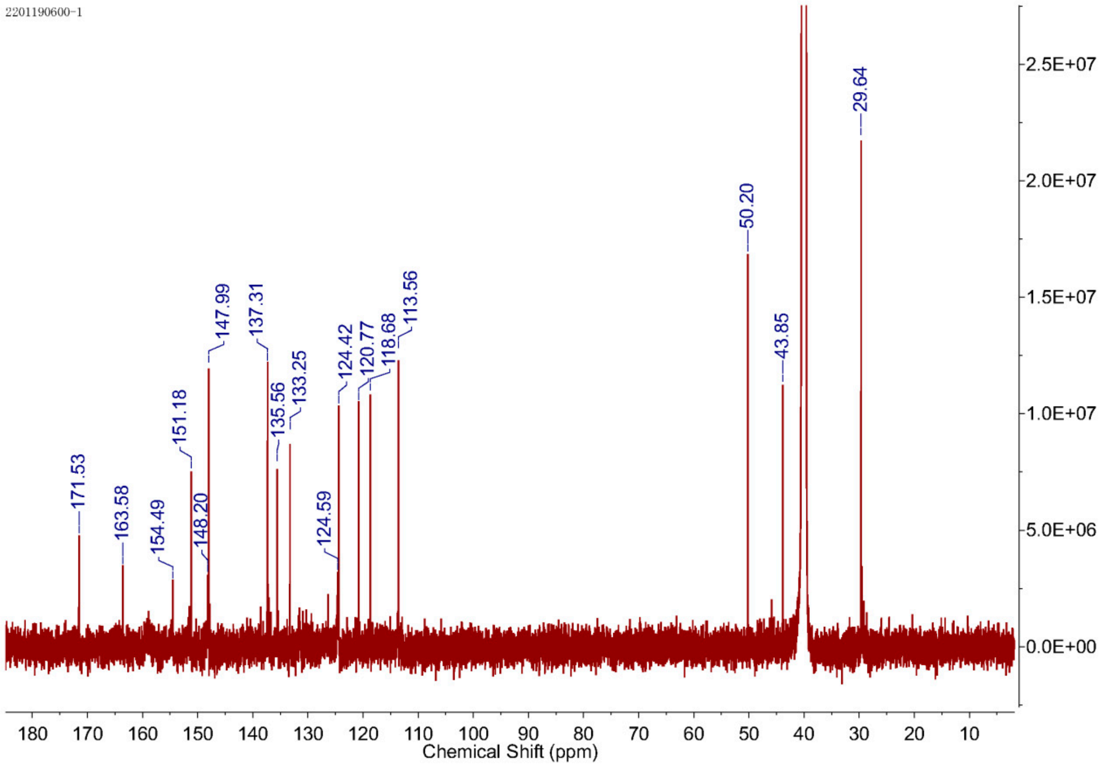
2201190600-1
171.53
163.58
154.49
151.18
148.20
147.99
137.31
135.56
133.25
124.59
124.42
120.77
118.68
113.56
50.20
43.85
29.64
2.5E+07
2.0E+07
1.5E+07
1.0E+07
5.0E+06
0.0E+00
180 170 160 150 140 130 120 110 100 90 80 70 60 50 40 30 20 10
Chemical Shift (ppm)

CA6-b

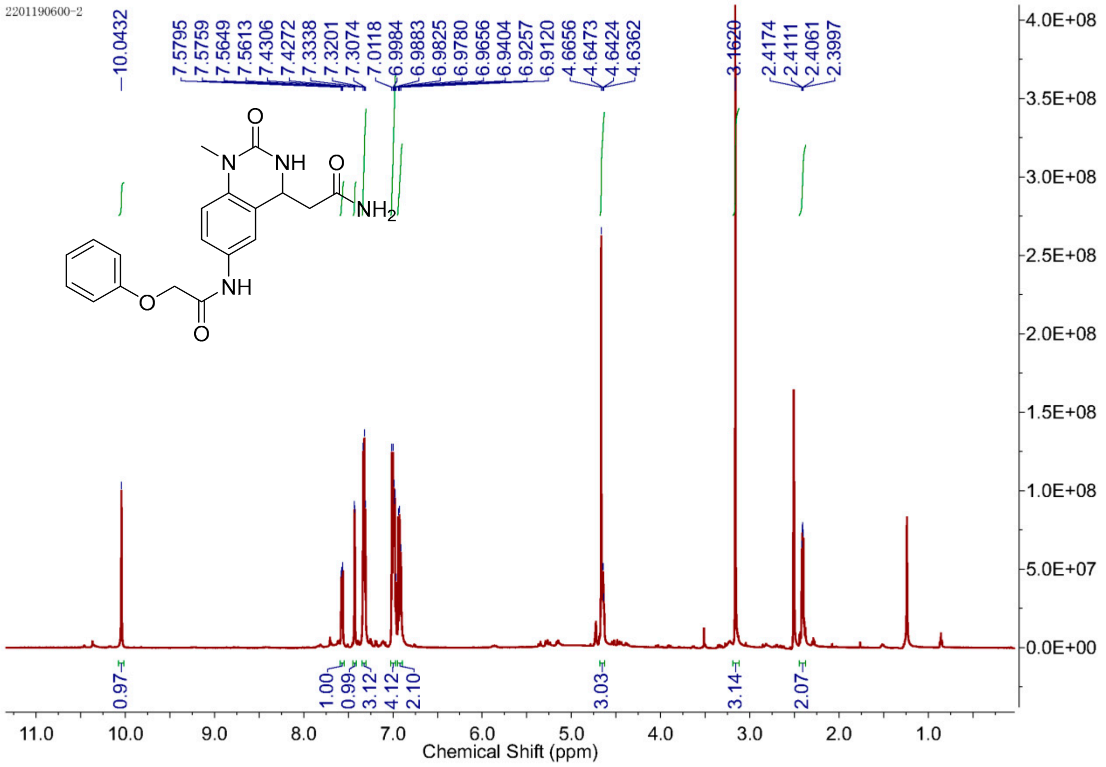

2201190600-2
10.0432
7.5795
7.5759
7.5649
7.5613
7.4306
7.4272
7.3338
7.3201
7.3074
7.0118
6.9984
6.9883
6.9825
6.9780
6.9656
6.9404
6.9257
6.9120
4.6656
4.6473
4.6424
4.6362
3.1620
2.4174
2.4111
2.4061
2.3997
O
N
NH
O
NH2
NH
O
O
0.97
1.00
0.99
3.12
4.12
2.10
3.03
3.14
2.07
Chemical Shift (ppm)

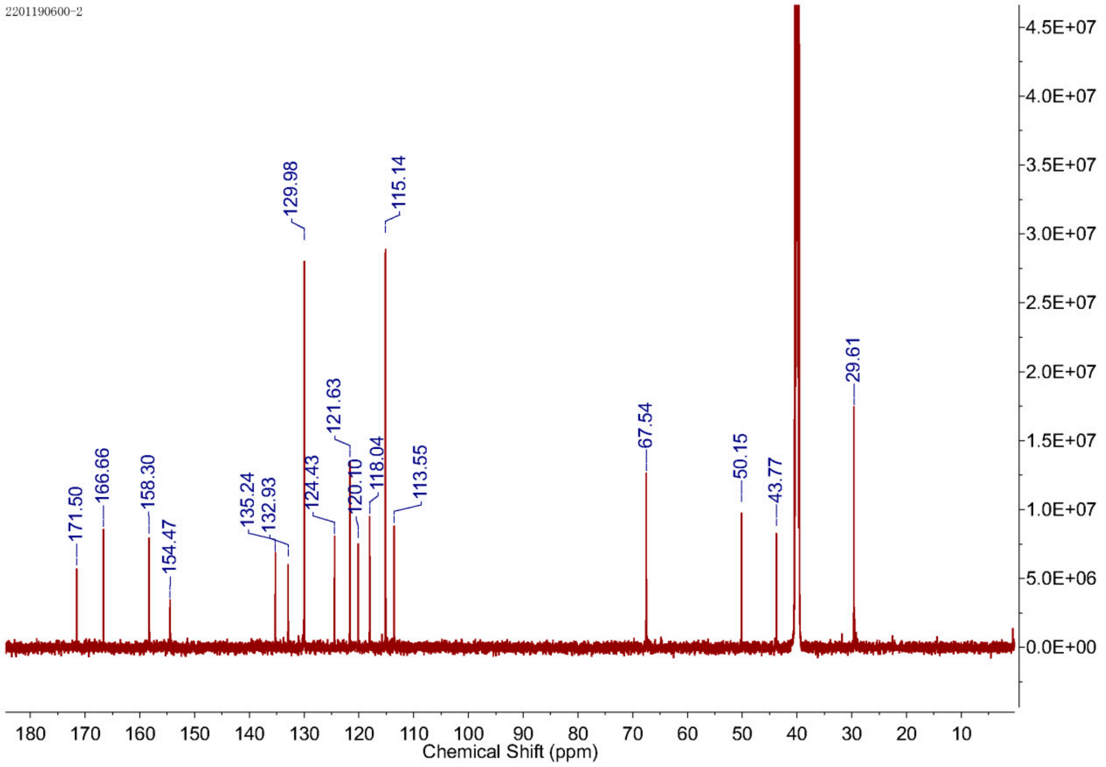

2201190600-2
171.50
166.66
158.30
154.47
135.24
132.93
129.98
124.43
121.63
120.10
118.04
115.14
113.55
67.54
50.15
43.77
29.61
Chemical Shift (ppm)

CA7-a

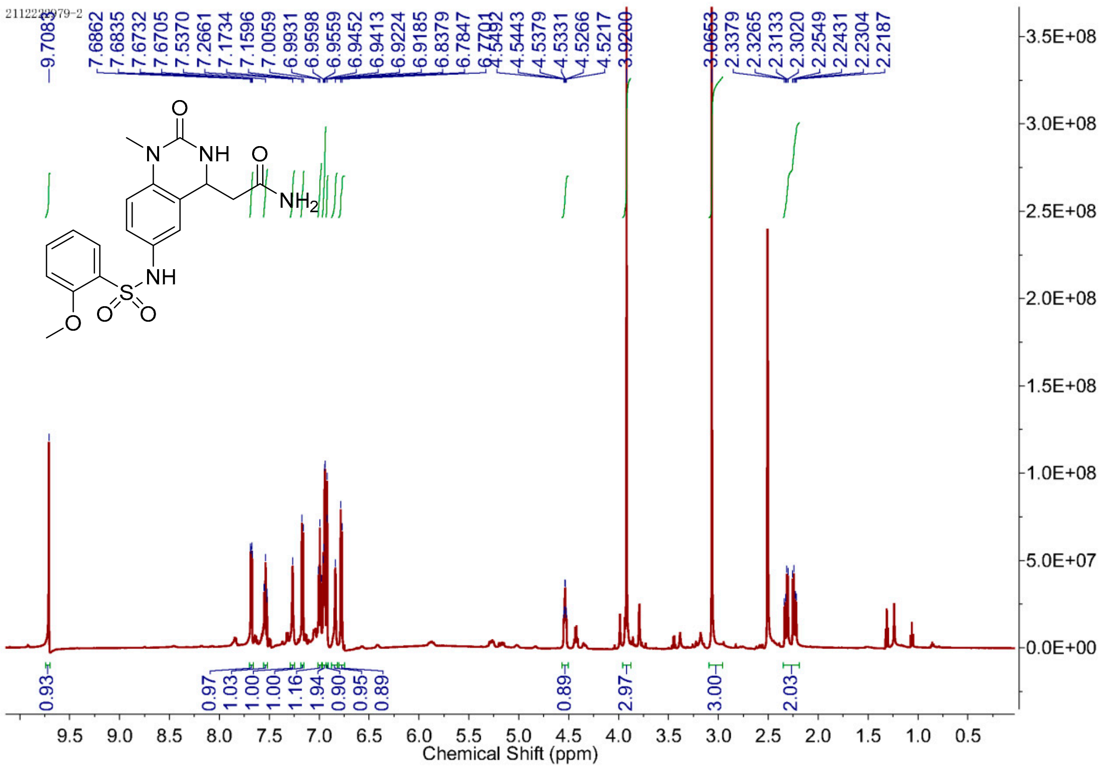
2112222979-2
9.7083
7.6862
7.6835
7.6732
7.6705
7.5370
7.2661
7.1734
7.1596
7.0059
6.9931
6.9598
6.9559
6.9452
6.9413
6.9224
6.9185
6.8379
6.7847
4.5443
4.5379
4.5331
4.5266
4.5217
3.9200
2.3379
2.3265
2.3133
2.3020
2.2549
2.2431
2.2304
2.2187
NH
O
NH
O
NH₂
NH
S
O
O
O
N
0.93
0.97
1.03
1.00
1.00
1.16
1.94
0.90
0.95
0.89
0.89
2.97
3.00
2.03
Chemical Shift (ppm)

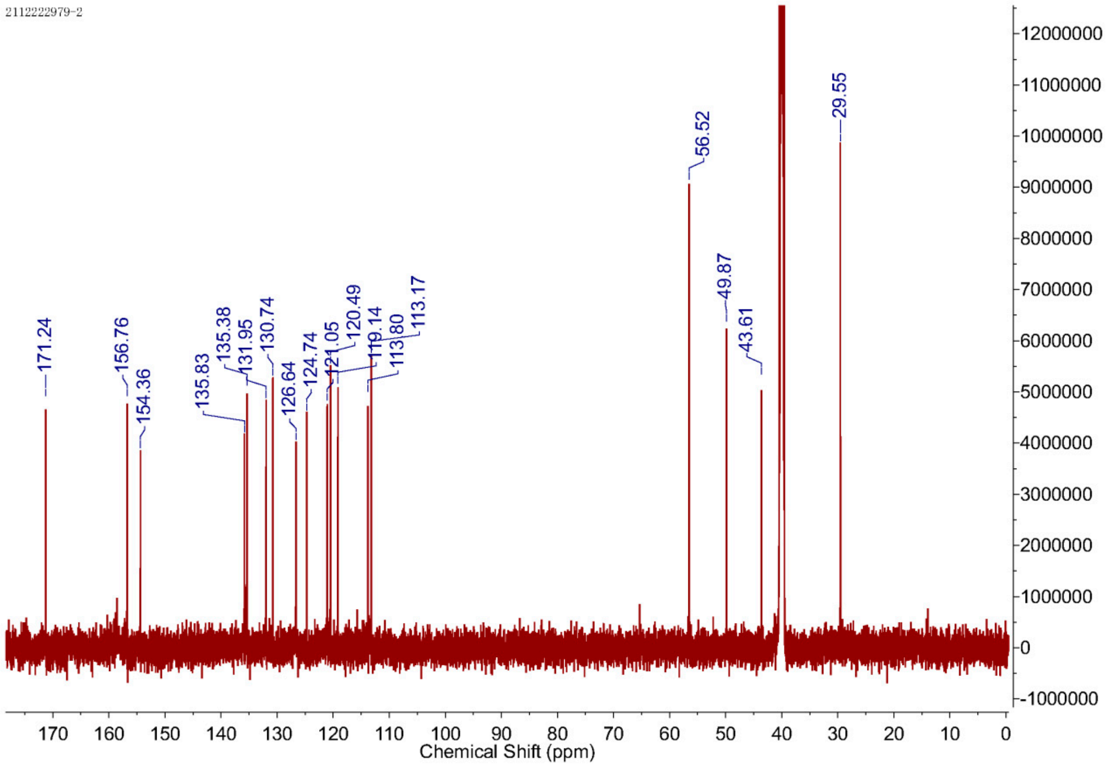
2112222979-2
171.24
156.76
154.36
135.83
135.38
131.95
130.74
126.64
124.74
121.05
120.49
119.14
113.80
113.17
56.52
49.87
43.61
29.55
Chemical Shift (ppm)

CA7-b'

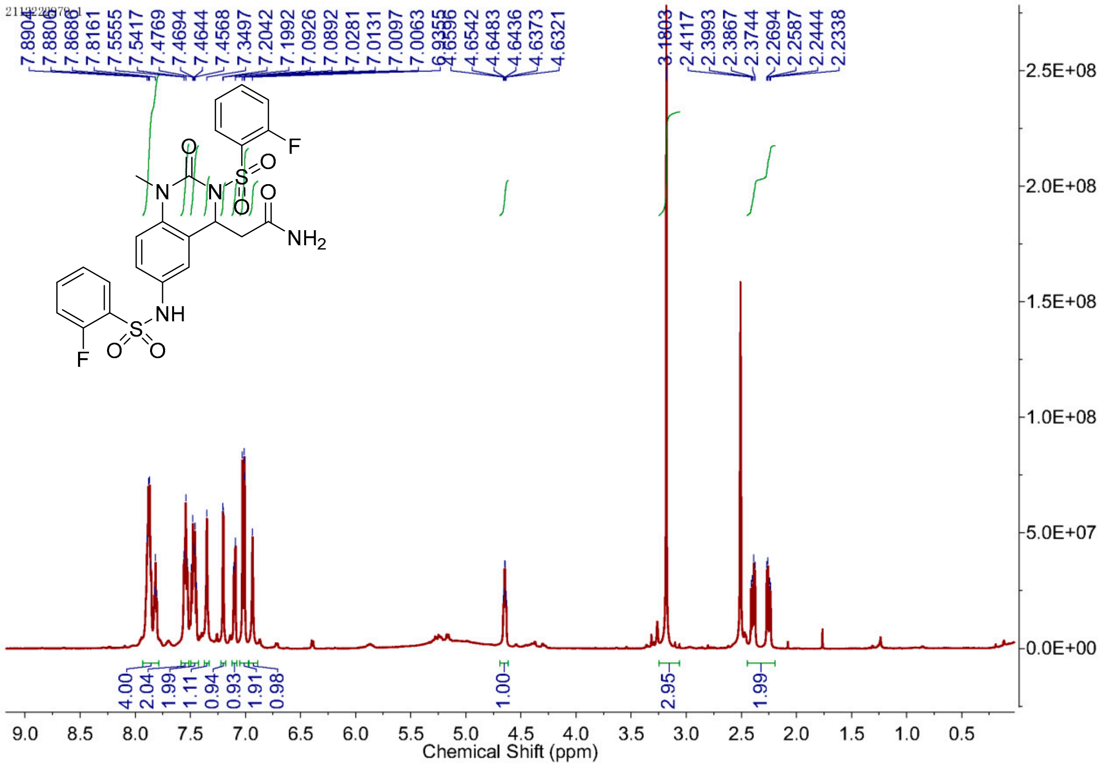
7.8904
7.8806
7.8686
7.8161
7.5555
7.5417
7.4769
7.4694
7.4644
7.4568
7.3497
7.2042
7.1992
7.0926
7.0892
7.0281
7.0131
7.0097
7.0063
4.6542
4.6483
4.6436
4.6373
4.6321
3.1803
2.4117
2.3993
2.3867
2.3744
2.2694
2.2587
2.2444
2.2338
F
O
N
N
S
O
O
O
NH2
NH
S
O
O
F
4.00
2.04
1.99
1.11
0.94
0.93
1.91
0.98
1.00
2.95
1.99
2.5E+08
2.0E+08
1.5E+08
1.0E+08
5.0E+07
0.0E+00
9.0 8.5 8.0 7.5 7.0 6.5 6.0 5.5 5.0 4.5 4.0 3.5 3.0 2.5 2.0 1.5 1.0 0.5
Chemical Shift (ppm)

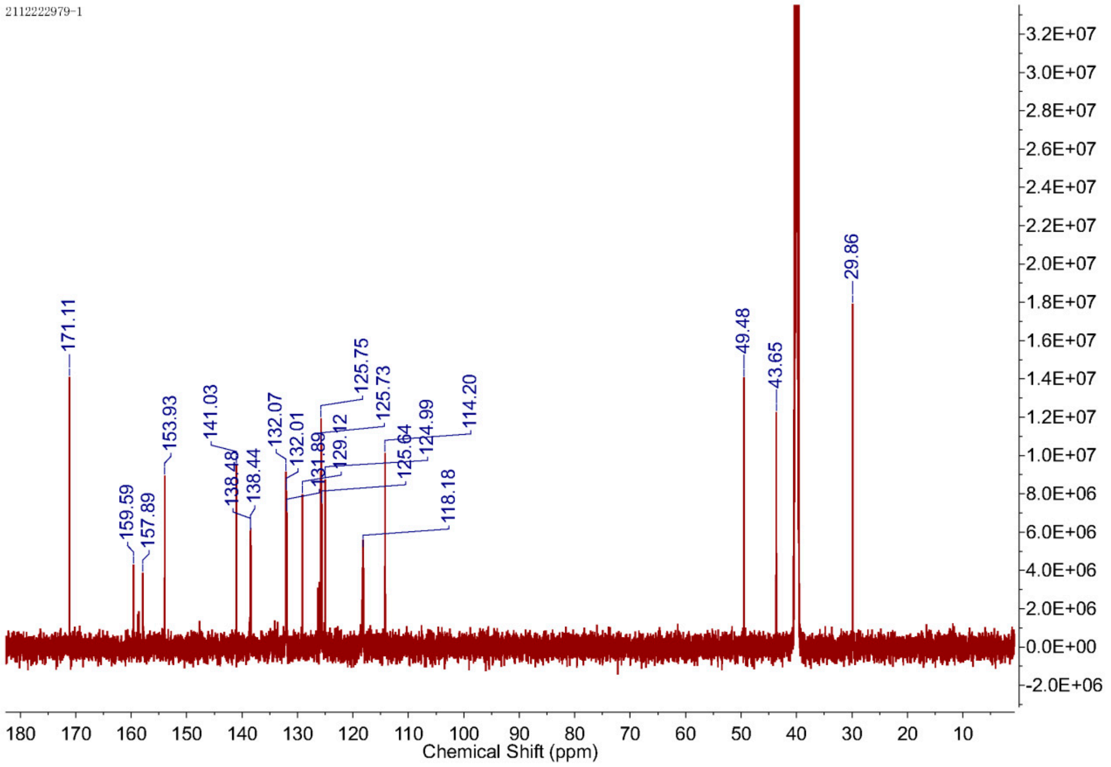
2112222979-1
171.11
159.59
157.89
153.93
141.03
138.48
138.44
132.07
132.01
131.89
129.12
125.75
125.73
125.64
124.99
118.18
114.20
49.48
43.65
29.86
3.2E+07
3.0E+07
2.8E+07
2.6E+07
2.4E+07
2.2E+07
2.0E+07
1.8E+07
1.6E+07
1.4E+07
1.2E+07
1.0E+07
8.0E+06
6.0E+06
4.0E+06
2.0E+06
0.0E+00
-2.0E+06
180 170 160 150 140 130 120 110 100 90 80 70 60 50 40 30 20 10
Chemical Shift (ppm)

LC/MS Spectrometry

CA1-6

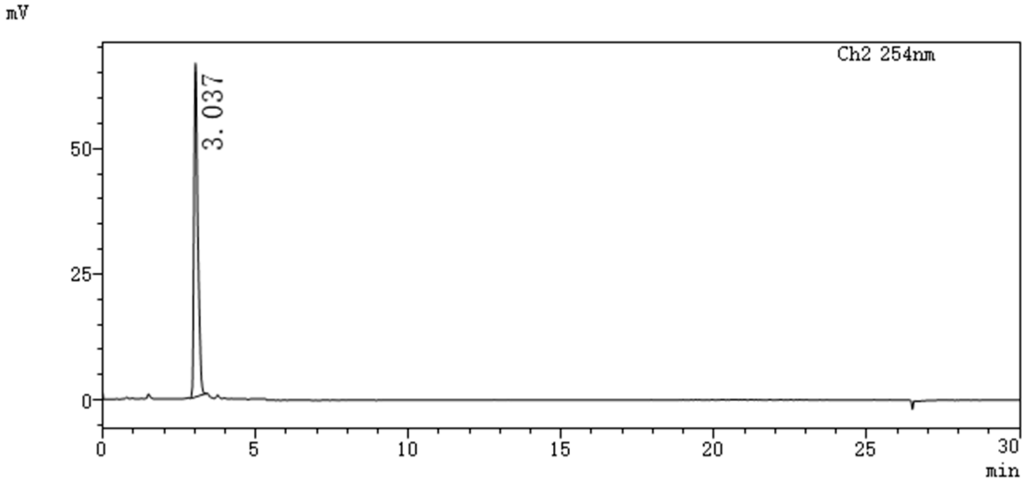

RT: 2.983 min (S#: 180)

Base peak: 395.15 (225870)

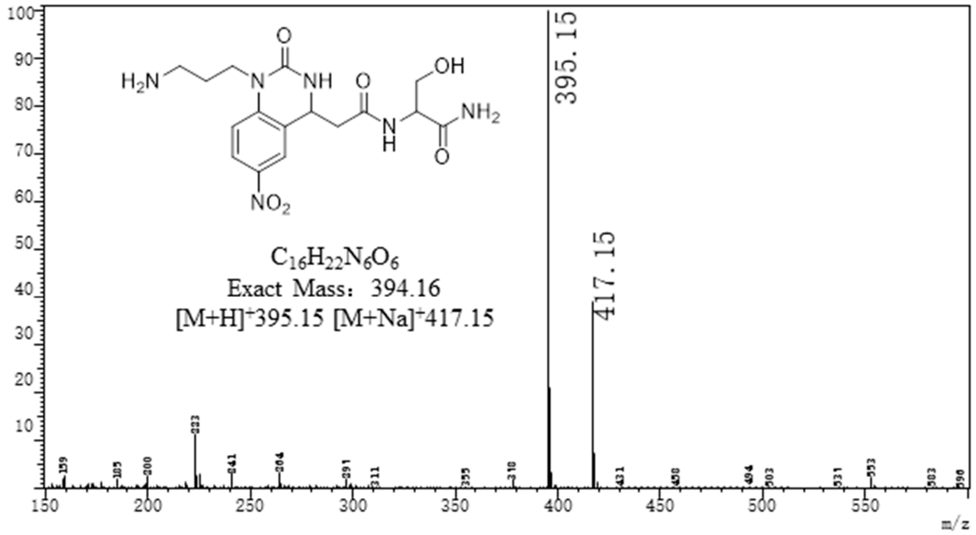

CA1-7

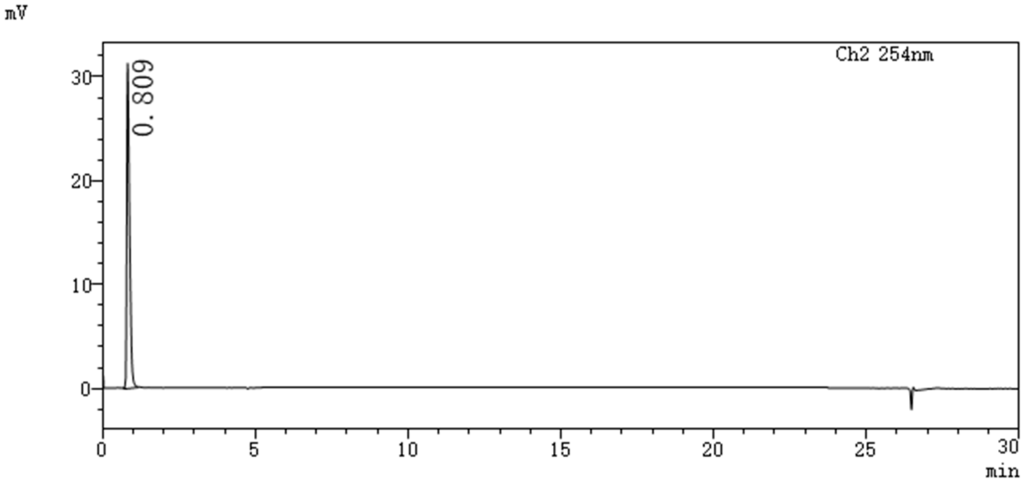
mV
Ch2 254nm
0.809
min

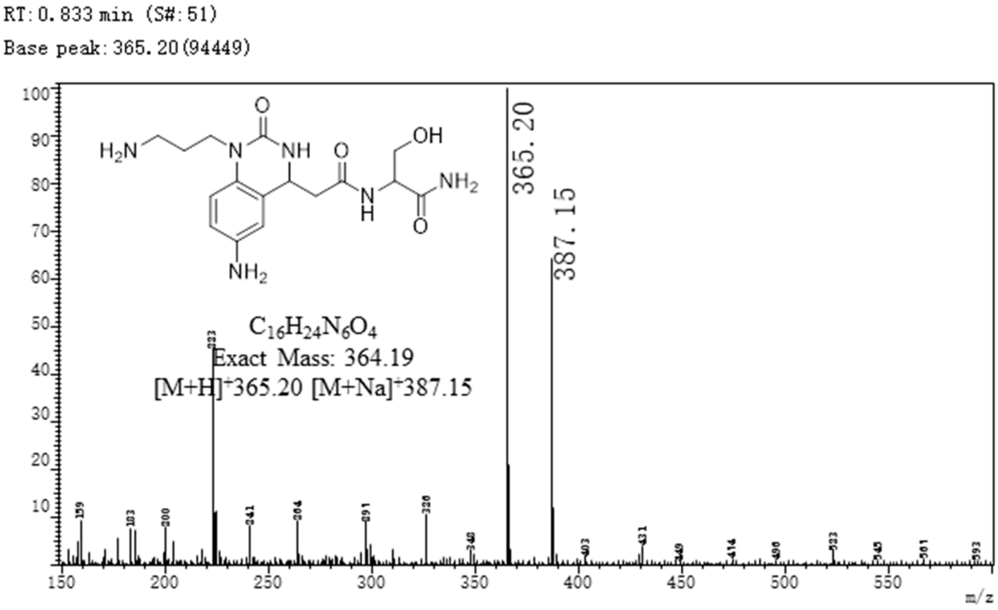
RT: 0.833 min (S#: 51)
Base peak: 365.20 (94449)
365.20
387.15
$C_{16}H_{24}N_6O_4$
Exact Mass: 364.19
$[M+H]^+$365.20 $[M+Na]^+$387.15
m/z

CA1-a

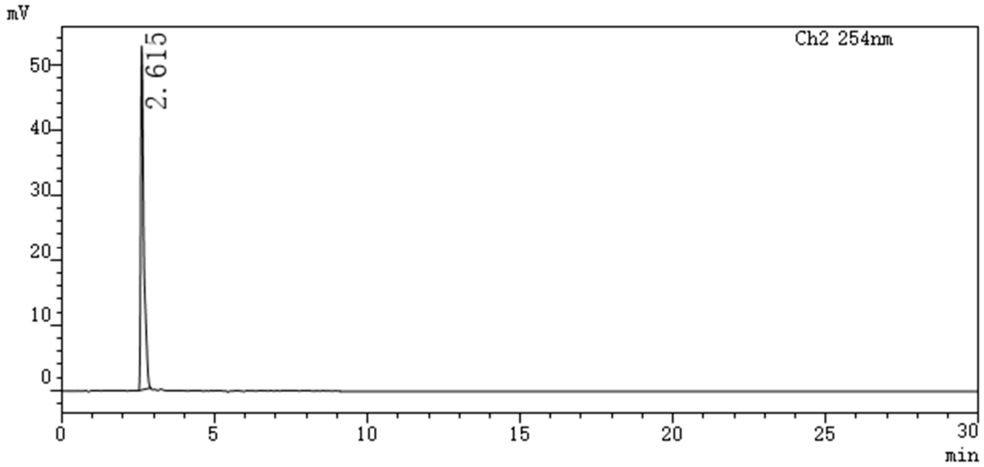
mV
Ch2 254nm
2.615
min

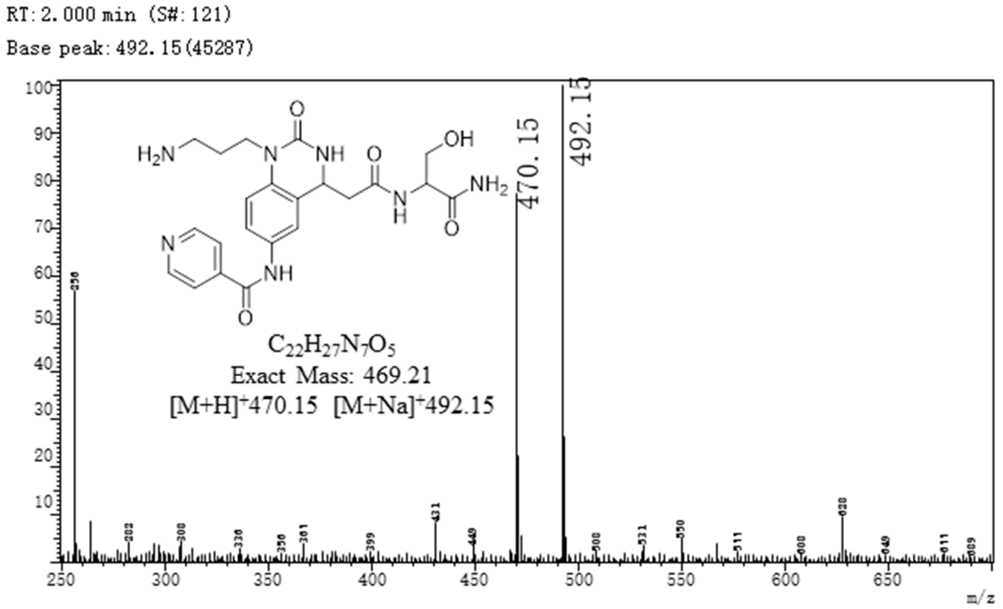
RT: 2.000 min (S#: 121)
Base peak: 492.15 (45287)
470.15
492.15
$C_{22}H_{27}N_7O_5$
Exact Mass: 469.21
[M+H]+470.15 [M+Na]+492.15
m/z

CA1-b

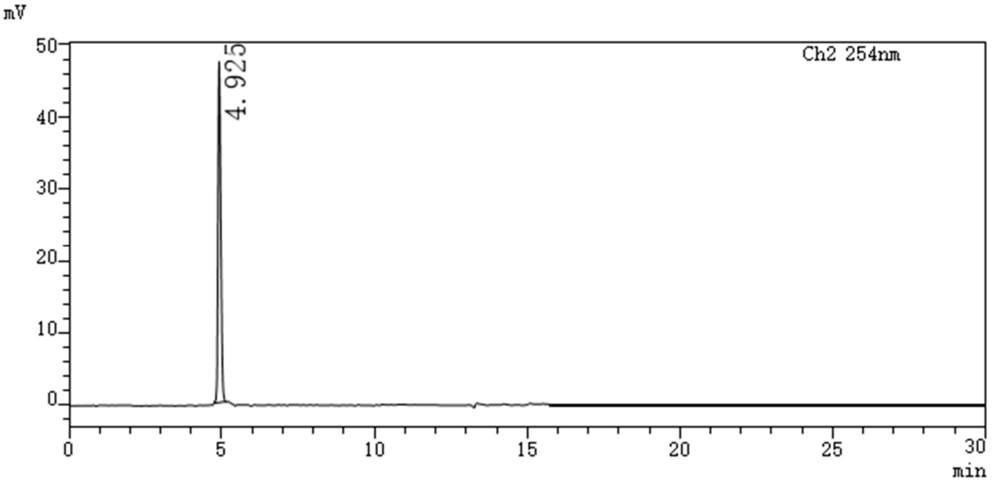

RT: 4.733 min (S#: 285)
Base peak: 469.20(350588)

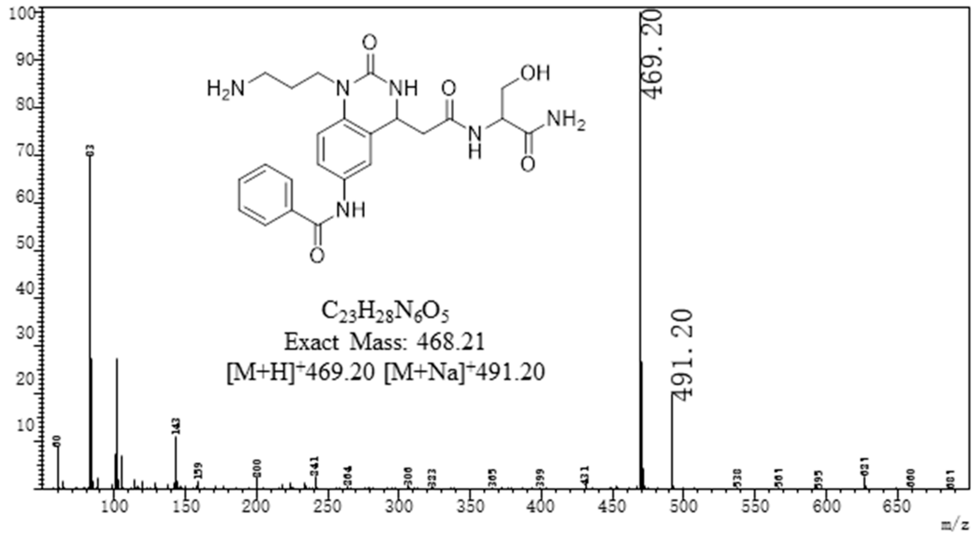

CA1-c

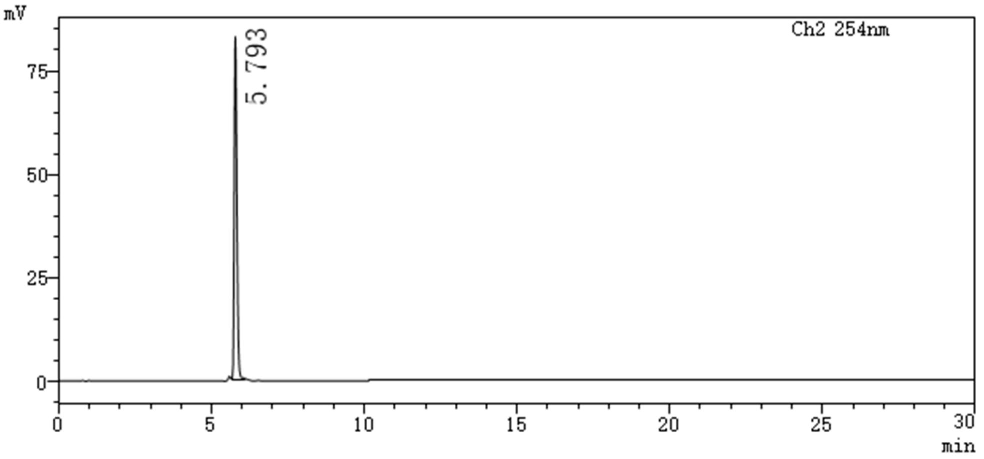

RT: 5.600 min (S#: 337)

Base peak: 483.20 (532953)

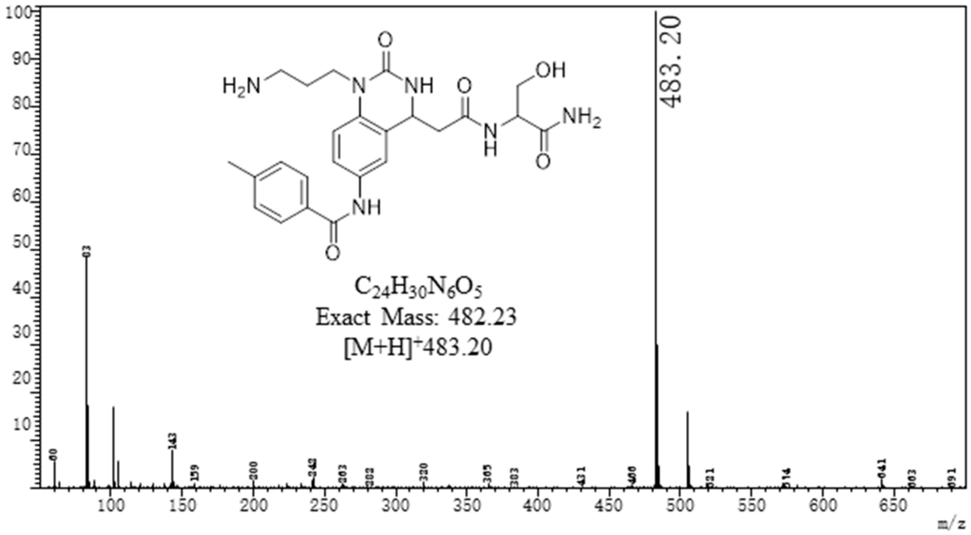

CA1-d

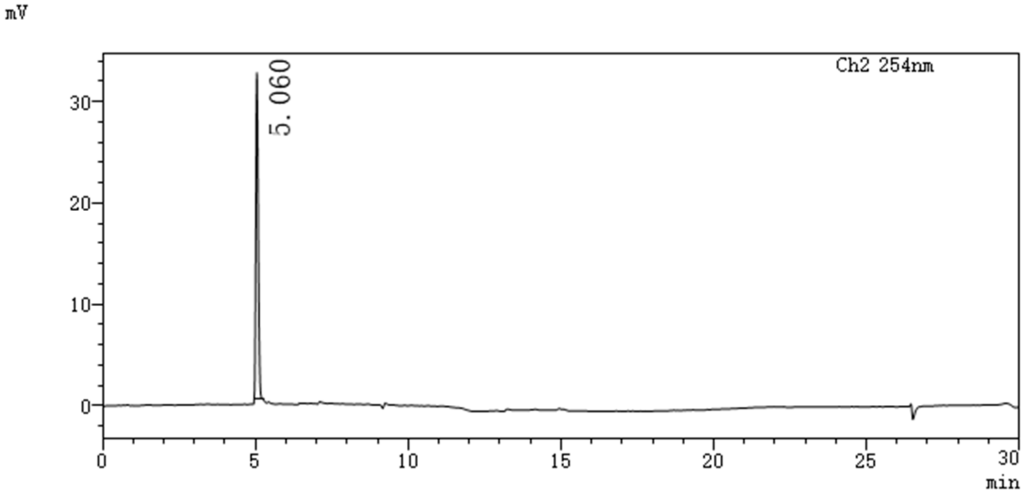

RT: 5.150 min (S#: 310)
Base peak: 487.20(233021)

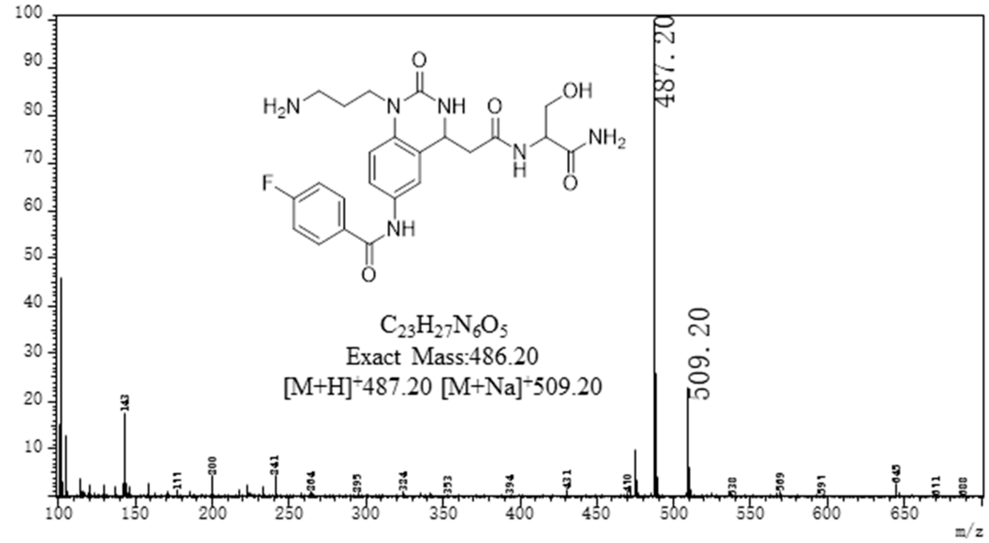

CA1-e

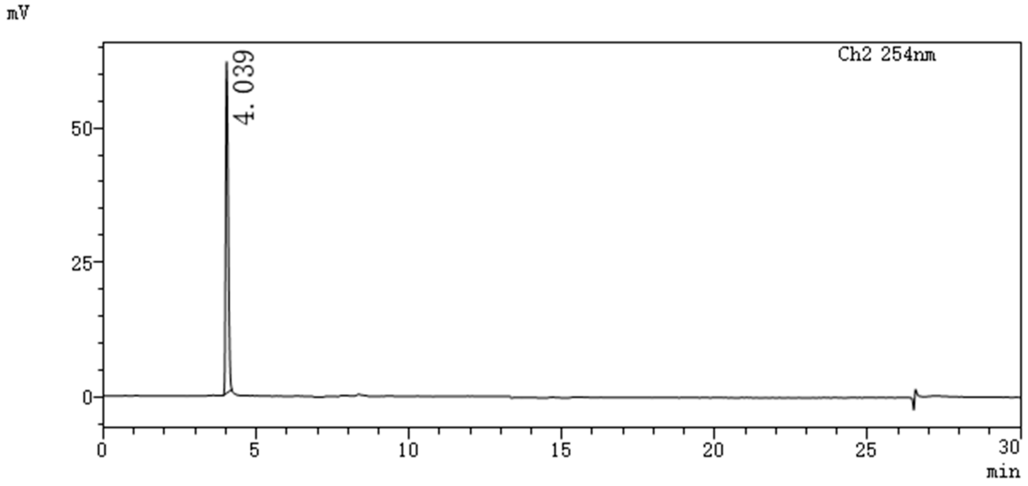

RT: 2.233 min (S#: 135)
Base peak: 470.20 (87027)

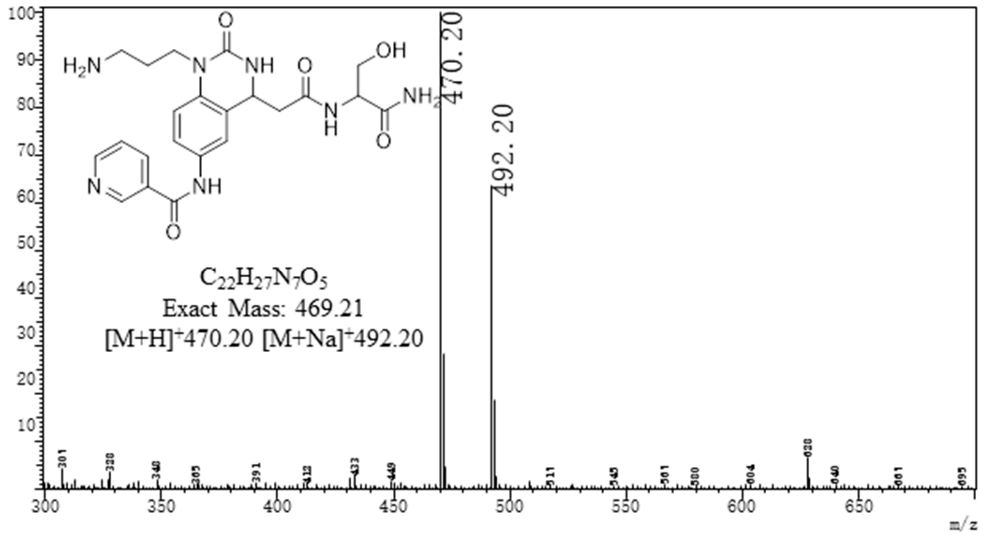

CA1-f

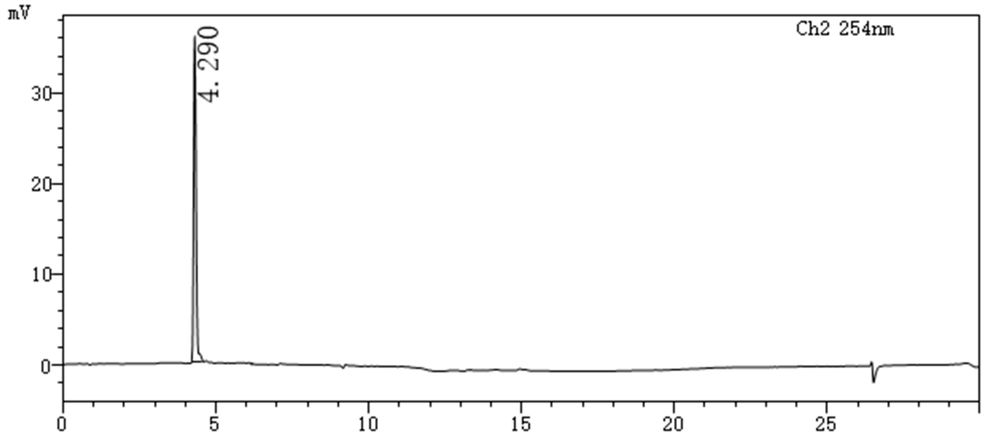

RT: 4.383 min (S#: 264)

Base peak: 470.15 (76198)

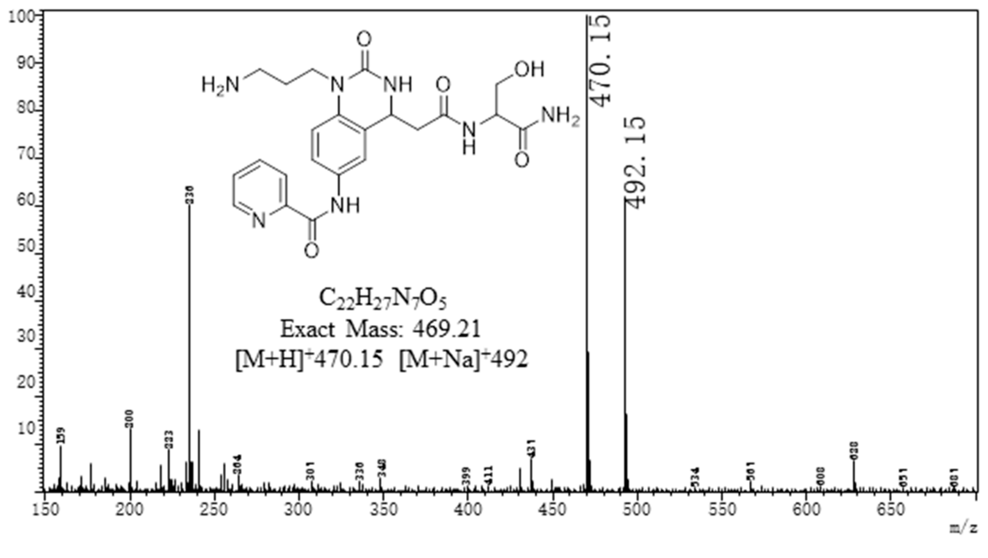

CA1-g

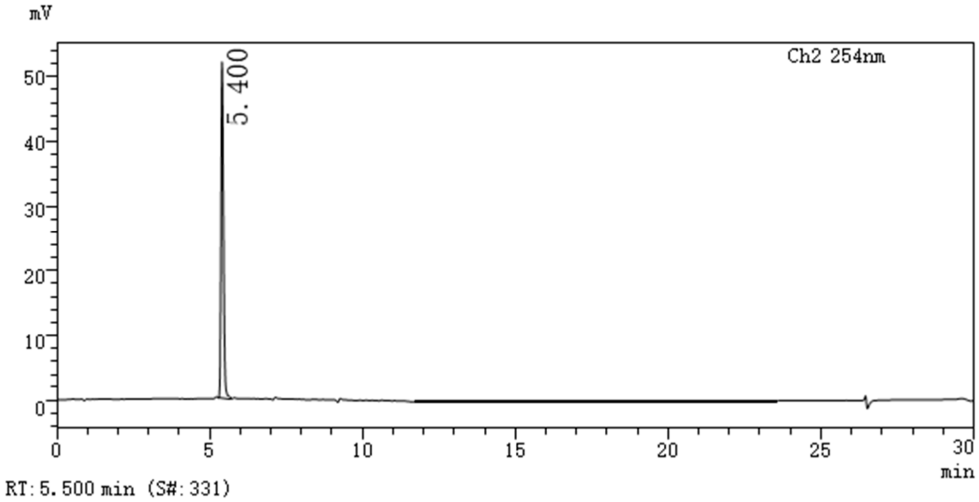

RT: 5.500 min (S#: 331)

Base peak: 499∘20(322733)

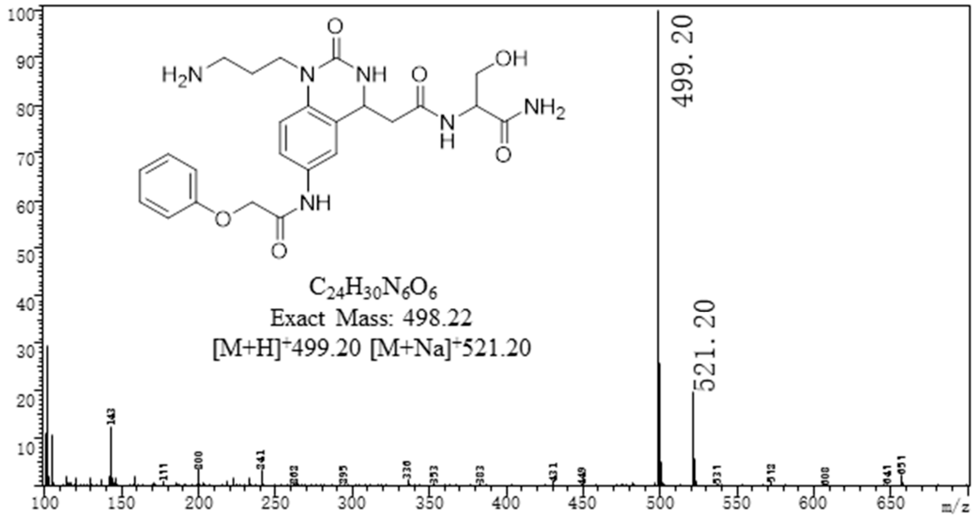

CA1-h

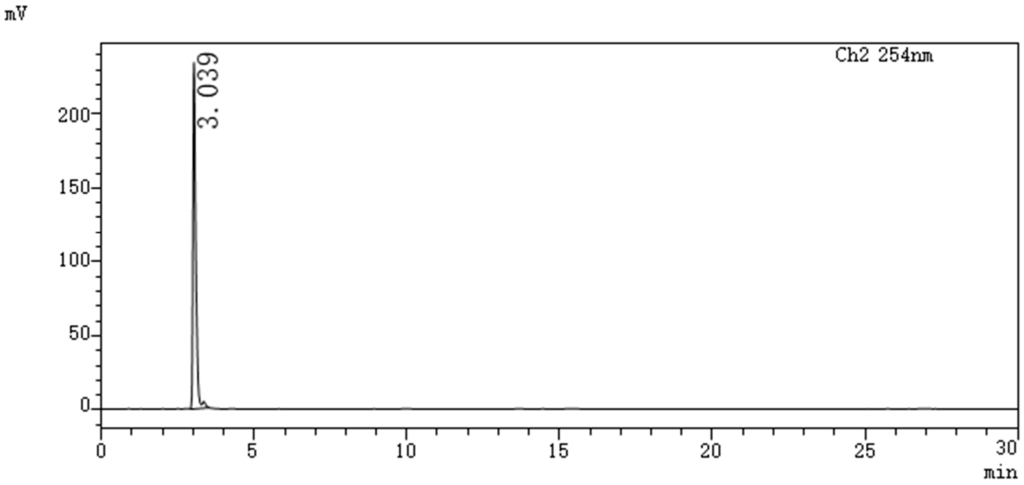

mV
Ch2 254nm
3.039
200
150
100
50
0
0
5
10
15
20
25
30
min

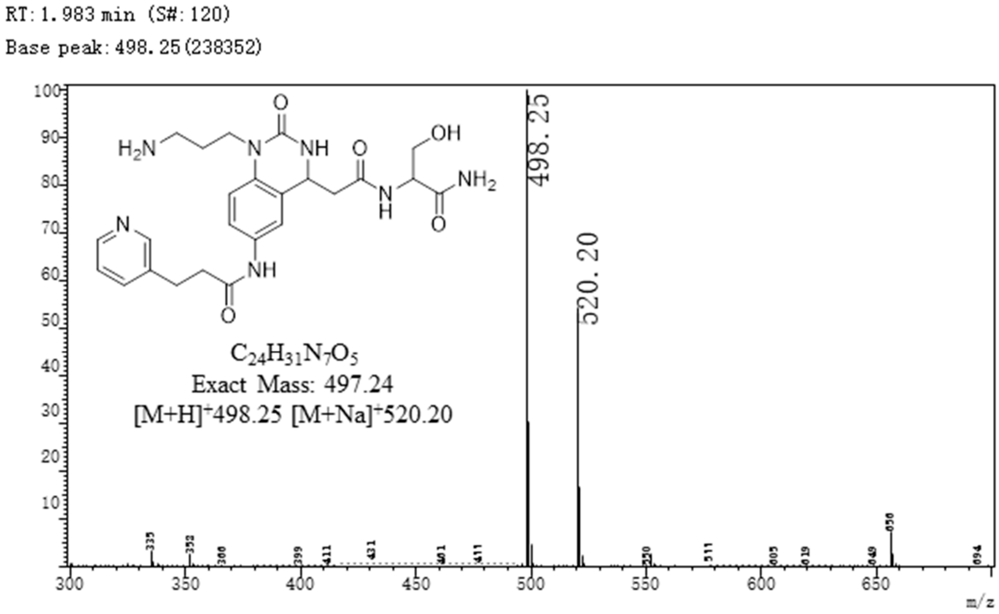

RT: 1.983 min (S#: 120)
Base peak: 498.25 (238352)
498.25
520.20
C24H31N7O5
Exact Mass: 497.24
[M+H]+498.25 [M+Na]+520.20
m/z

CA1-i

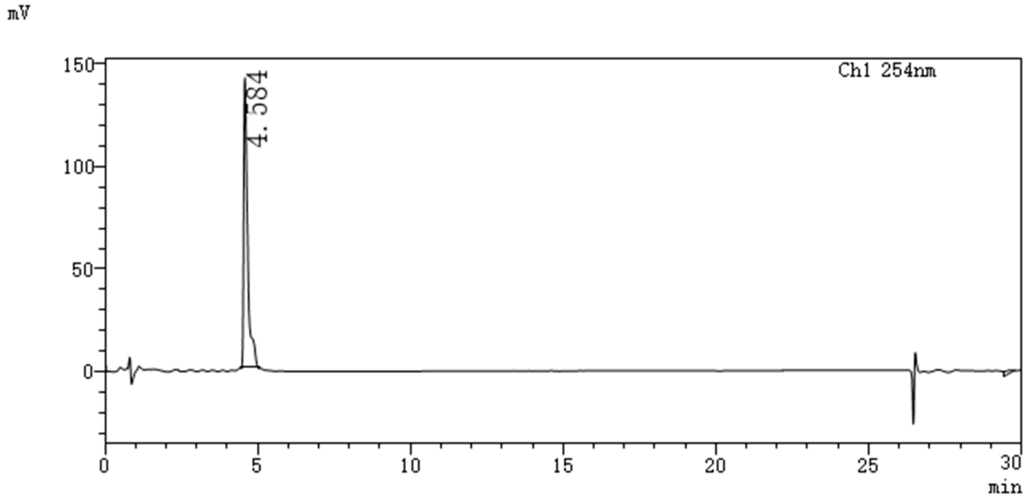

RT: 4.800 min (S#: 289)
Base peak: 489.20(161523)

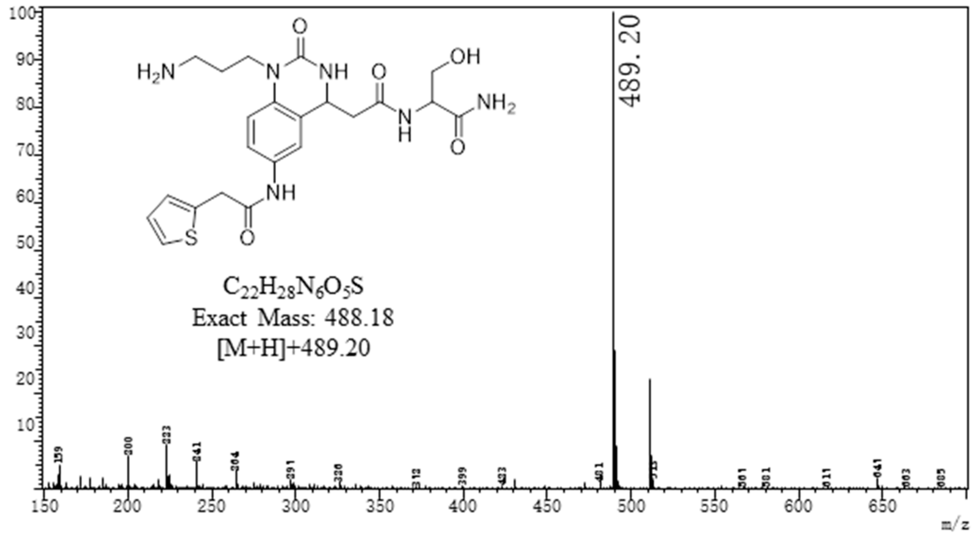

CA1-j

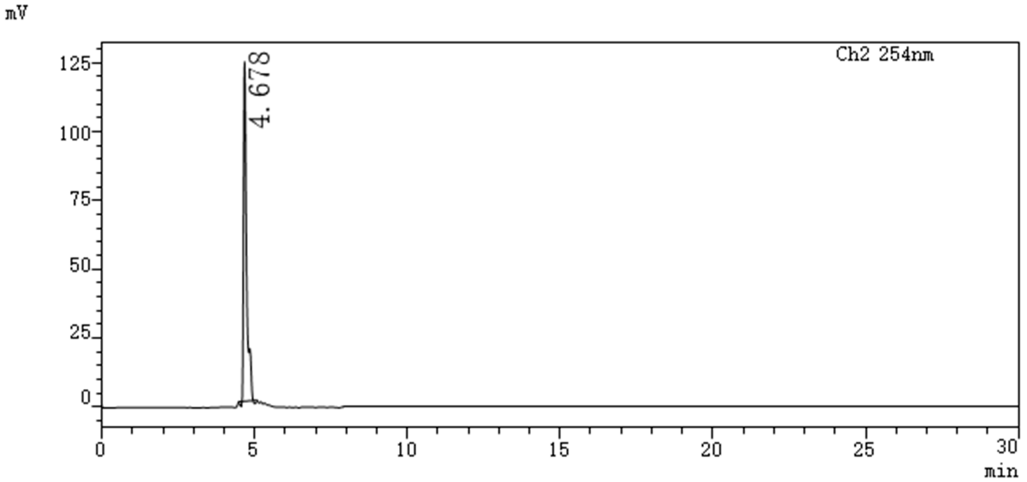

RT: 4.733 min (S#: 285)
Base peak: 535.20 (125733)

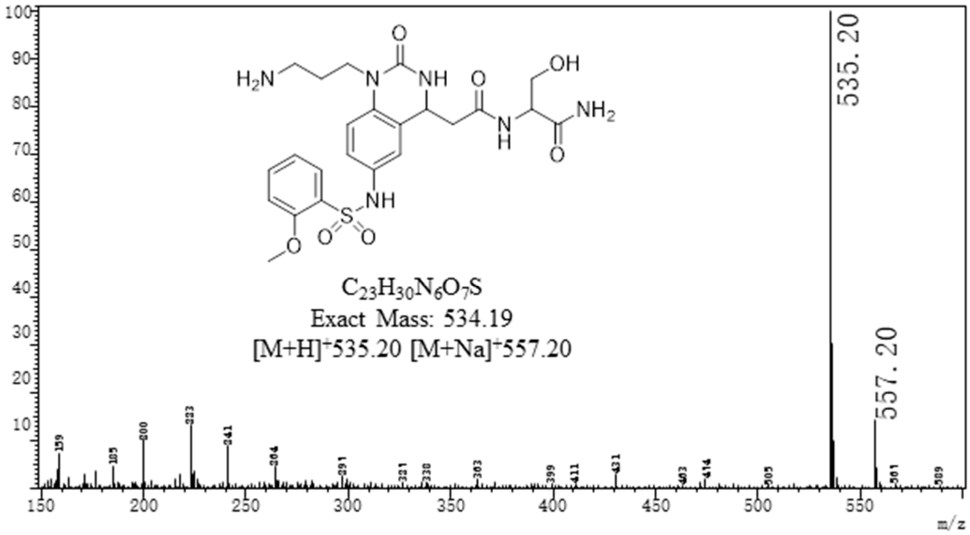

CA3

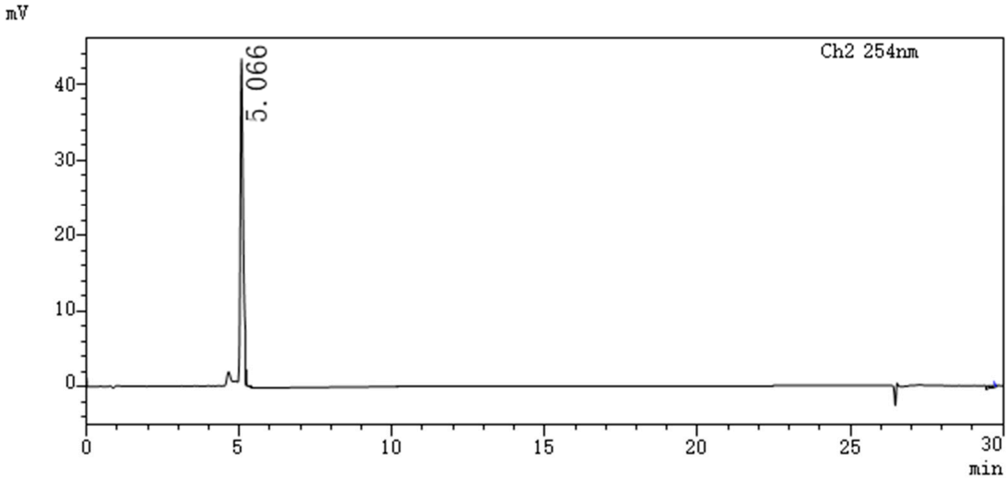

RT: 6.583 min (S#: 396)
Base peak: 538.30(3551384)

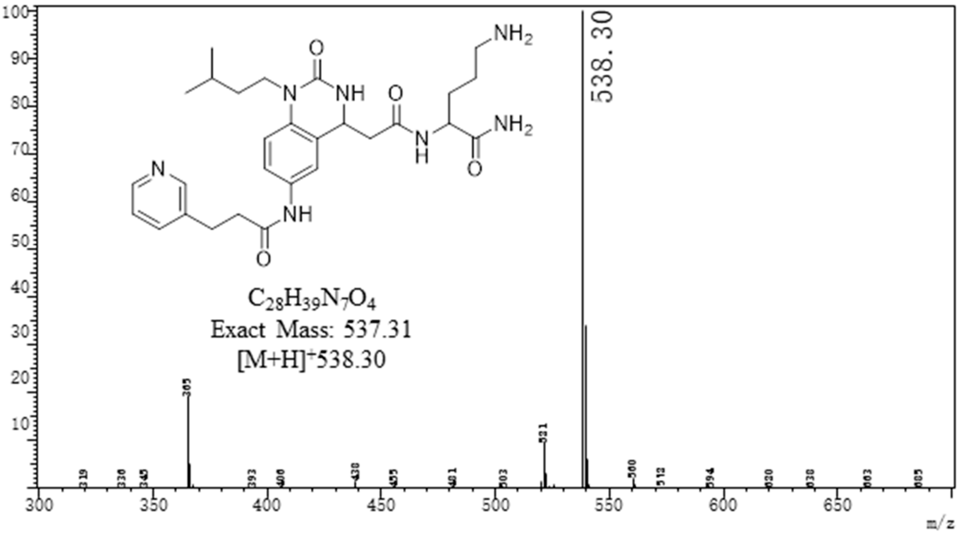

CA4

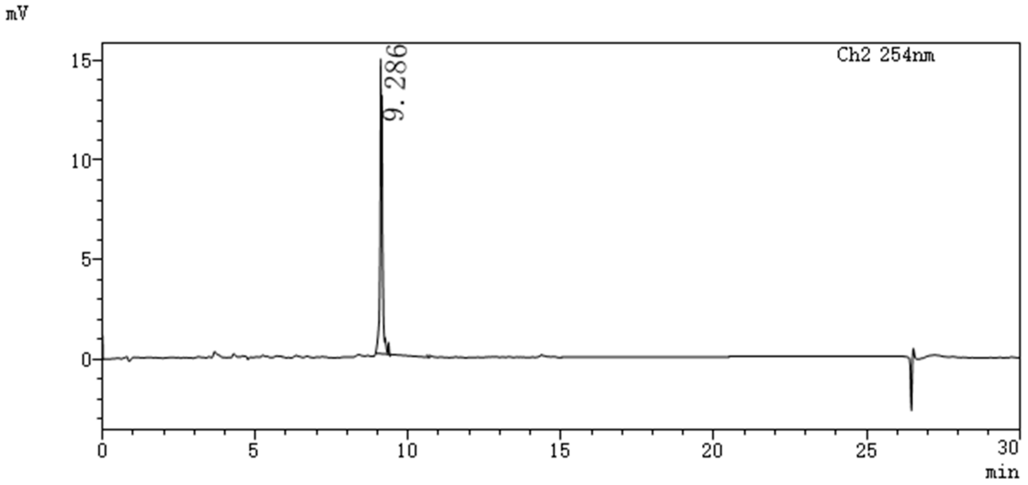

RT: 9.483 min (S#: 570)
Base peak: 530.25(28473)

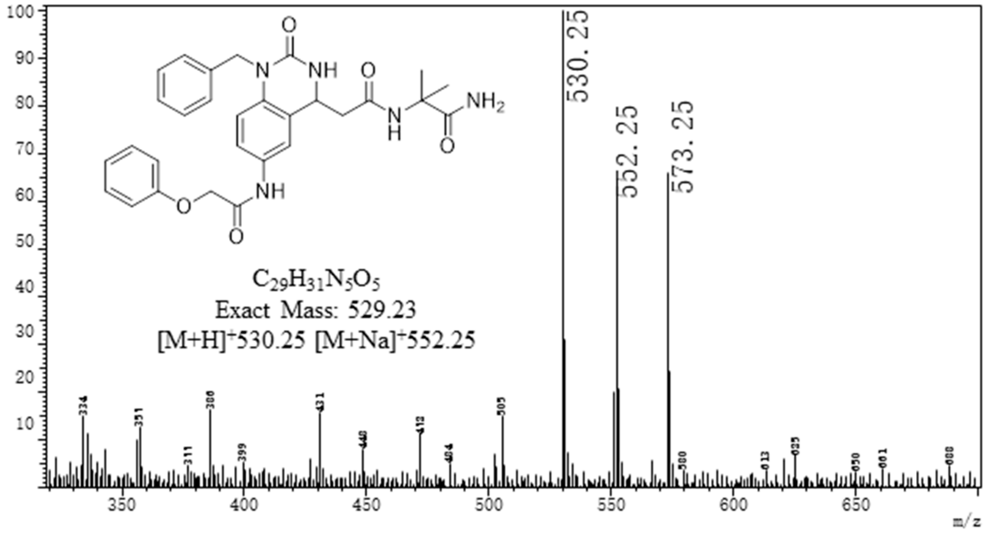

CA5

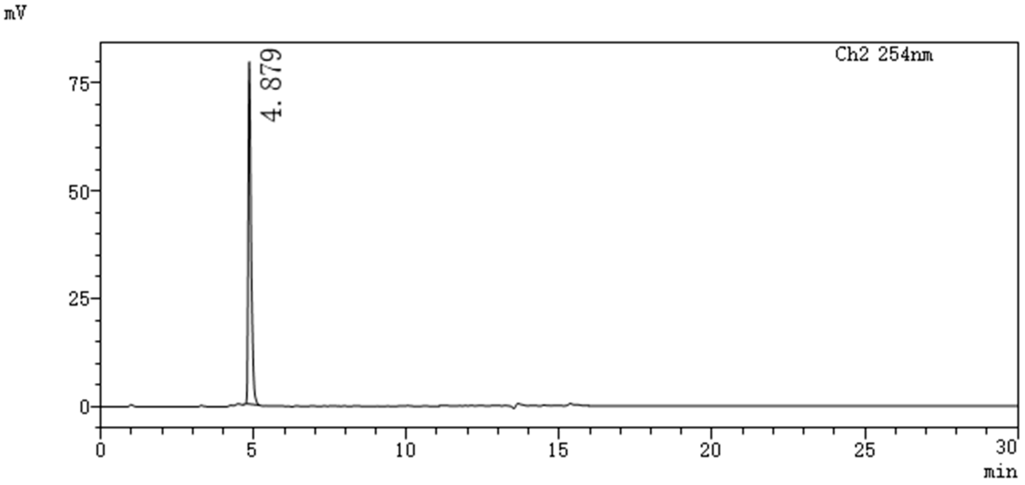

RT: 4.367 min (S#: 263)
Base peak: 528.20 (138703)

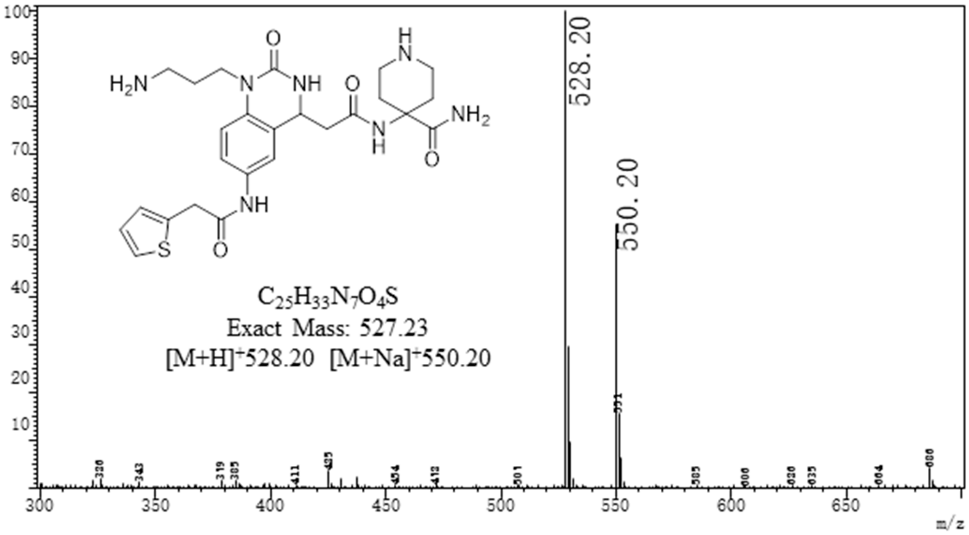

CA6-a

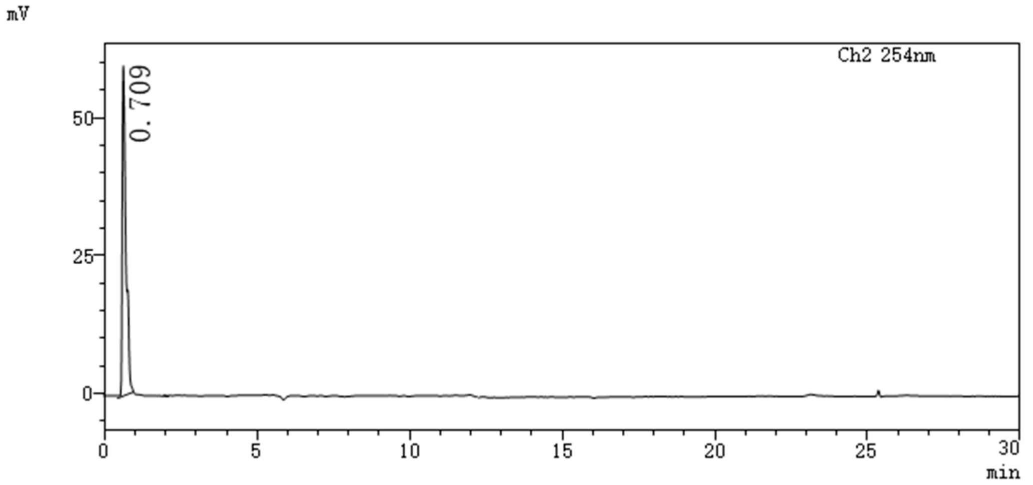

RT: 0.667 min (S#: 41)
Base peak: 340.15 (82566)

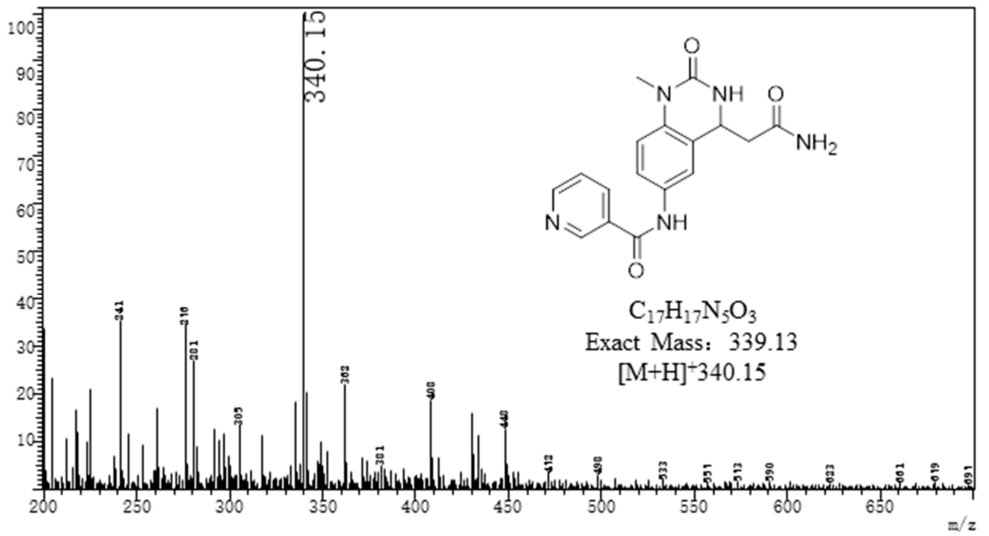

CA6-b

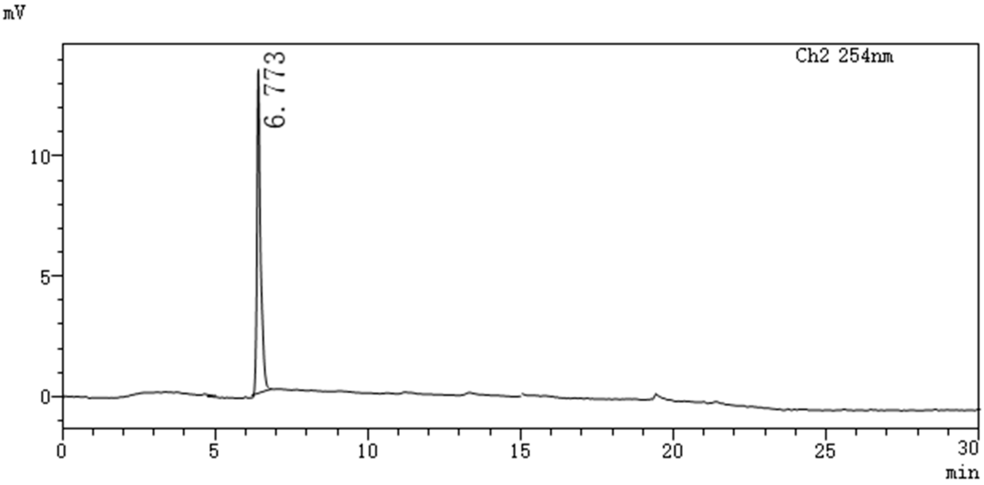

RT: 6.867 min (S#: 413)
Base peak: 369.15(71969)

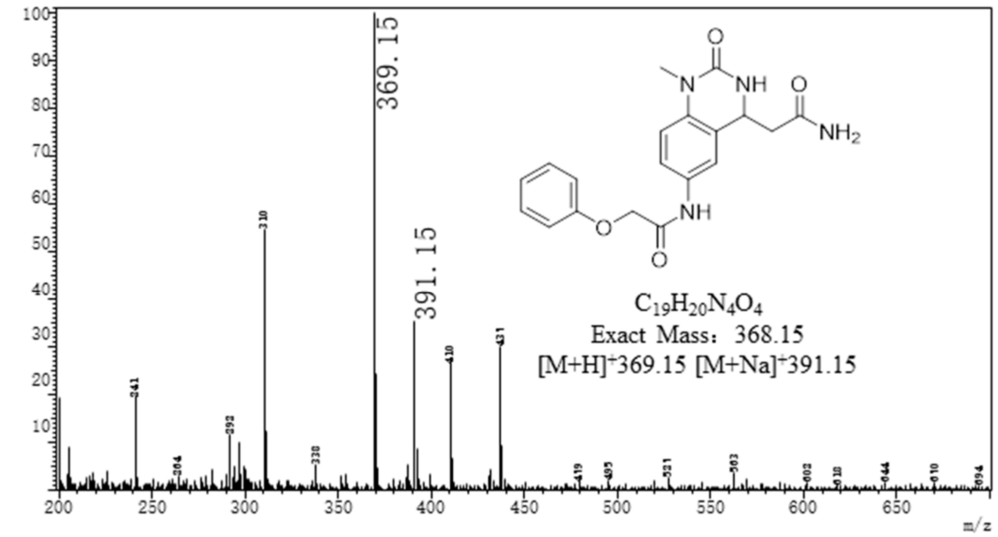

CA7-a

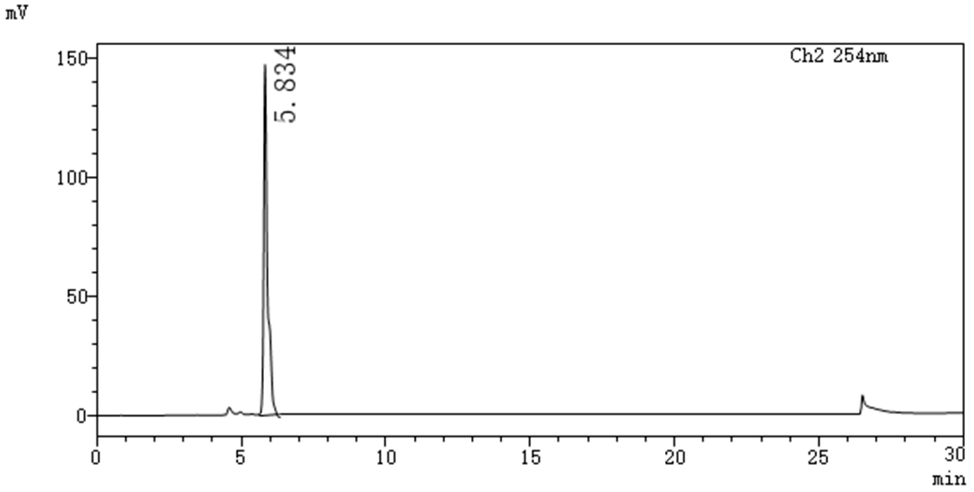

RT: 6.000 min (S#: 361)

Base peak: 405.10(204349)

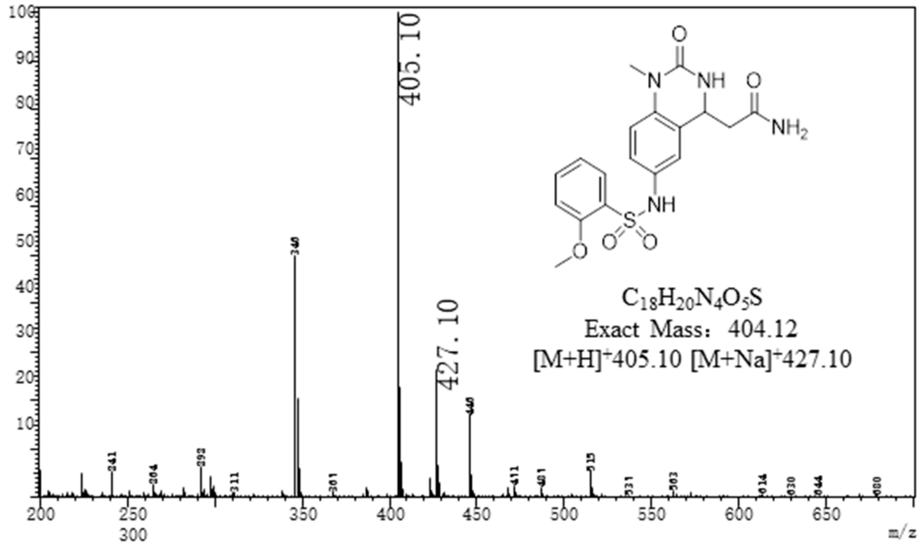

CA7-b'

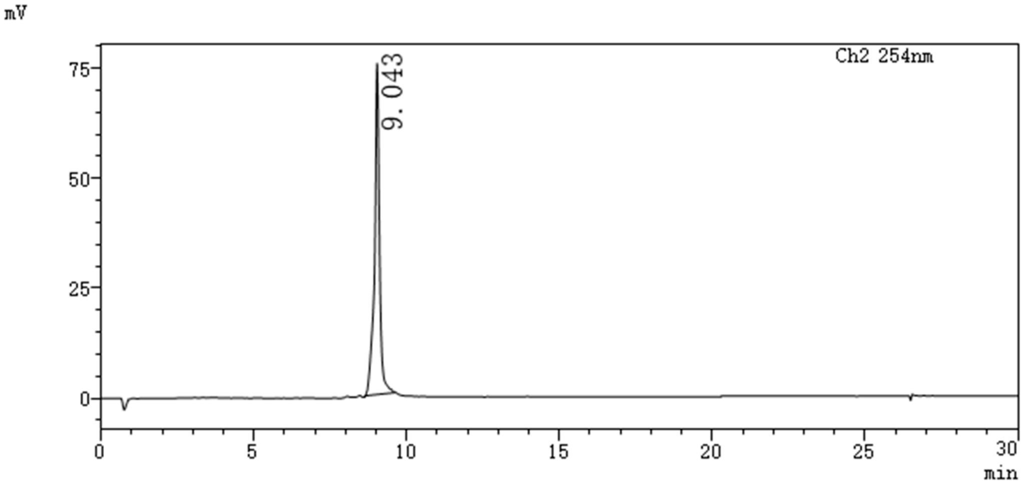
mV
Ch2 254nm
9.043
min

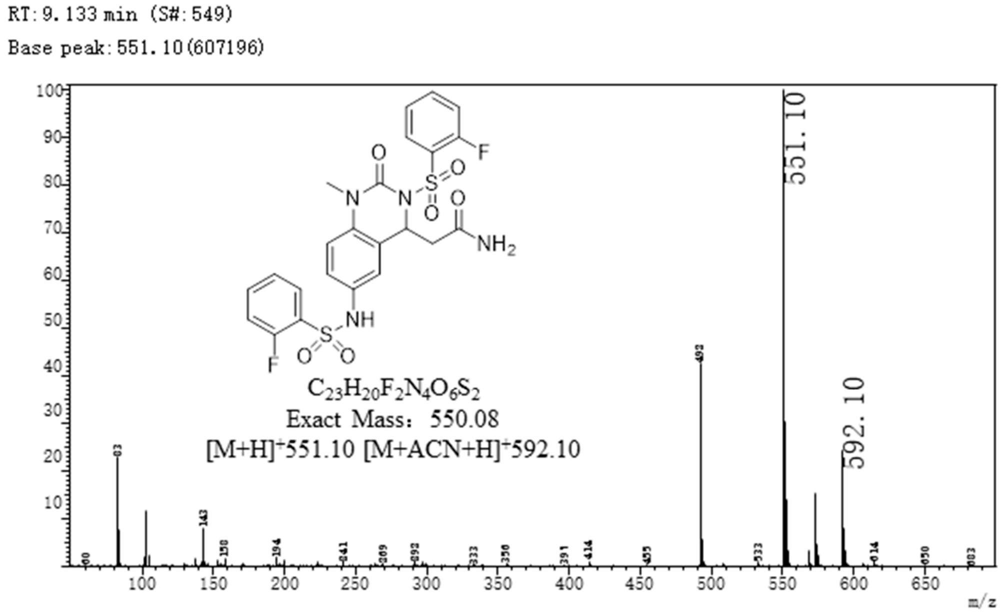
RT: 9.133 min (S#: 549)
Base peak: 551.10(607196)
551.10
592.10
$C_{23}H_{20}F_2N_4O_6S_2$
Exact Mass：550.08
$[M+H]^+$551.10 $[M+ACN+H]^+$592.10
m/z
